# Assessing geographic variation in women’s decision-making power across 720 districts in India, 2016–2021

**DOI:** 10.1186/s12905-025-04194-0

**Published:** 2025-12-18

**Authors:** Yun-Jung Eom, Shalem Balla, Sunil Rajpal, Rockli Kim, S. V. Subramanian

**Affiliations:** 1 Interdisciplinary Program in Precision Public Health, Department of Public Health Sciences, Graduate School of Korea University, Seoul, South Korea; 2 School of Humanities and Social Sciences, Indian Institute of Technology, Mandi, Himachal Pradesh India; 3 Department of Economics, FLAME University, Pune, India; 4 Division of Health Policy and Management, College of Health Science, Korea University, Seoul, South Korea; 5 Harvard Center for Population and Development Studies, Cambridge, MA USA; 6 Department of Social and Behavioral Sciences, Harvard T. H. Chan School of Public Health, Boston, MA USA

**Keywords:** Women's decision-making, Women's empowerment, Geographic disparities, District-level variation, India

## Abstract

**Background:**

Women’s ability to make decisions in personal and family matters is a crucial component of their agency, often shaped by geographically clustered and hierarchically structured social and cultural factors. Despite its importance, district-level research on women’s decision-making in India is scarce, especially considering the country’s diverse socio-cultural contexts. This study examines women’s participation in decision-making across 720 districts in India, with a focus on exploring the geographical patterns of variation.

**Methods:**

Using data from the 2016 and 2021 rounds of the National Family Health Survey, we analyzed data from currently married women aged 15–49 years in India. The final analytical sample included 86,694 women from 2016 to 76,910 women from 2021. We employed a four-level logistic regression model using Markov Chain Monte Carlo estimation to examine geographic patterns of women’s decision-making across 720 districts in India.

**Results:**

Women’s participation in household decision-making increased between 2016 and 2021, with the median district prevalence rising from 85.38% to 89.87%. In 2016, relatively low levels of participation in decision-making (< 80%) were geographically widespread across districts. By 2021, women’s decision-making participation increased in 312 districts (≥ 5.00%), whereas declines were observed in 100 districts based on a more conservative threshold (≤–2.5%). Both improvements and declines were geographically dispersed, indicating that changes in women’s decision-making power did not follow a clear regional pattern.

**Conclusion:**

While women’s decision-making power in India has improved overall, the direction and magnitude of change continue to vary widely from district to district across the country. This observed geographic heterogeneity underscores persistent structural and normative barriers to women’s agency. Addressing these geographic disparities requires context-specific policies that are responsive to local socio-cultural dynamics, including family structures and gender norms.

**Supplementary Information:**

The online version contains supplementary material available at 10.1186/s12905-025-04194-0.

## Introduction

Women’s ability to make decisions on critical matters such as their own health, household purchases and visits to family and friends, is central to their empowerment and essential for improving not only their own health but also that of their children and the broader community [1, 2]. Women’s decision-making power is also a core component of the United Nations Sustainable Development Goal (SDG) 5 [3], which seeks to achieve gender equality and empower all women and girls. Recognizing decision-making as a key indicator of women’s agency underscores its cross-cutting influence on health, education, and economic participation, making it a global priority for gender-equitable development. Women with greater decision-making power demonstrate higher utilization of maternal health services, improved fertility control, and better health outcomes [4–9]. Recent evidence further suggests that decision-making autonomy can serve as a protective factor against intimate partner violence, enabling women to negotiate or exit abusive relationships [10]. Empirical studies have also established links between women’s empowerment and key reproductive outcomes, including fertility preferences, family planning, contraceptive use, and fertility rates [11–13]. Given this, the government of India has made heavy investments to foster enabling conditions that strengthen women’s ability to make independent decisions. For the fiscal year 2024–2025, the government earmarked three trillion INR for initiatives aimed at empowerment of women [14]. Such initiatives have largely focused on enhancing women’s education, paid employment, and financial inclusion [15, 16]. Despite these efforts, improvements in women’s ability to make their own decisions remain limited [17], as education and employment alone have been identified as insufficient to strengthen women’s empowerment [18].

Policy approaches aimed at enhancing women’s decision-making power have often underestimated the significant influence of cultural and social structures. These structures, such as family networks, kinship ties, religious groups, and broader social norms, exert significant control over women’s roles through the values, expectations, and laws they impose [8]. Deeply rooted hierarchical systems, particularly within families, often allocate authority based on age and gender, with men typically holding decision-making power and women occupying subordinate positions [19]. These dynamics are further reinforced by practices such as traditional inheritance laws and restrictive gender norms, which dictate what is considered acceptable behavior for women and limit their autonomy. Importantly, the influence of these cultural and social forces varies substantially across smaller geographic areas. As a result, centrally designed, one-size-fits-all policy solutions may interact differently with local realities, leading to heterogeneous outcomes.

While women’s decision-making power is restricted by structural and institutional constraints such as patriarchal family systems, traditional norms, and unequal access to resources, it is also shaped by the dynamic spread of ideas through cultural diffusion and interpersonal interaction [20]. Ideas, attitudes, and practices circulate through social contact, as individuals and communities exchange information and influence one another [21]. The expansion of digital platforms, including social media, telecommunications, and online entertainment along with improved transportation, has accelerated the flow of ideas across geographic and social boundaries. However, exposure to these influences is neither universal nor uniform. Individuals vary in their access to and adoption of new ideas, which tend to take root through repeated interactions among geographically proximate people. This results in clustering of shared norms and behaviors, which then aggregate at higher spatial levels such as communities, villages, or regions, ultimately shaping the local context in which women’s decision-making power is either constrained or enabled.

Given that cultural practices, development, and social structures are geographically clustered, women’s decision-making power appears to have a pronounced geographical dimension. This study draws on Kishor’s (2000) conceptual framework, which identifies three distinct stages: “settings” as the social and cultural environment, “sources” as enabling resources and “evidence” as empowerment outcomes such as women’s participation in decision-making [22]. Particularly, the concept of “settings” aligns with the geographical notion of place, defined as the cultural, historical, and institutional character of a location that shapes gender norms and expectations. These place-based influences often operate hierarchically across geographical levels, from communities to districts and states [23, 24], contributing to intra- and inter-district variation in women’s decision-making power. For example, while some districts may offer expanded employment schemes for women, others remain constrained by traditional kinship-based systems. Even within the same administrative unit, women’s ability to exercise agency may vary across villages or between urban and rural settings.

Thus, there is significant value in examining women’s decision-making power at geographically disaggregated levels. While similar approaches have been previously undertaken in India, most studies have focused on the sub-national level of states or have been limited to specific aspects of women’s empowerment, such as intimate partner violence [25–27]. States often mask substantial intra-state heterogeneity, as programmatic and social realities vary greatly across districts. Given that districts are the primary administrative units for policy implementation and planning in India, district-level estimation of women’s decision-making provides a valuable basis for assessing policy effectiveness. It offers a more actionable understanding of where progress is concentrated or lagging, thereby enabling targeted policy design and local implementation. To fill this gap, we employ small-area estimation techniques and a unique methodology to provide district-level representative estimates of women’s decision-making across 720 districts in India, using the latest geographical delineation of the country.

## Methods

### Data and sampling strategy

This analysis used data from the fourth and fifth rounds of India’s National Family Health Survey (NFHS) conducted in 2015–2016 and 2019–2021, respectively [30, 31]. For simplicity, from this point forward, we will refer exclusively to the terminal year of each survey. Both surveys are part of the Demographic and Health Surveys Program (DHS) and collect data on population health, nutrition, and well-being. Among the diverse topics covered, the surveys include indicators related to women’s participation in decision-making. Both surveys were structured to identify clusters – rural villages and urban wards – using probability proportionate to size from districts within states. Households were then randomly selected from these clusters. Detailed information regarding the sampling methodology is provided in the latest NFHS (2021) report [17].

### District geometry

This study aims to examine district-level variations in women’s participation in decision-making across India and track changes between 2016 and 2021. Due to administrative reorganizations, the number of districts in India has increased from 640 in 2016 to 707 in 2021. To ensure comparability, we used a 2022 configuration of 720 districts, which incorporates the 13 new districts created in Andhra Pradesh (AP) in April 2022. The methodological process for updating and harmonizing district boundaries has been described in detail elsewhere [28]. Incorporating these new districts is essential as the 2021 districts from AP do not align with the updated district boundaries, preventing accurate geographic interpretation within the state.

### Study population

We analyzed data from women aged 15–49 years in 2021 and 2016 surveys. In both datasets, only currently married women in part of the state module were eligible to respond to questions related to participation in household decision-making.

In the 2016 dataset, 122,351 women were initially included in the state module. Of these, 35,540 women who were not currently married at the time of the survey were excluded. An additional 117 women were dropped due to unmatched cluster identifiers following the restructuring into 720 districts, resulting in a final analytical sample of 86,694 women. In the 2021 dataset, a total of 108,785 women were initially included in the state module. After excluding 31,875 women who were not currently married, the final sample comprised 76,910 women. In both survey rounds, there were no missing values for the outcome variables.

### Outcome variable

The outcome of this study was women’s participation in decision-making, operationalized through a composite outcome derived from three variables. Questionnaires asked who usually decides on three domains: *women’s healthcare*, *large household purchases*, and *visits to family or relatives*. Each question had five response options: ‘women alone’, ‘with husband/partner’, ‘husband/partner alone’, ‘someone else’, and ‘other’. Women were classified as participating in decision-making if they reported making decisions either alone or jointly with their husband/partner in any of the three domains. This approach follows the academic definition of decision-making power as women’s ability to take strategic actions to attain their self-defined goals [29], and is also consistent with prior studies [30, 31] that treated both independent and joint decision-making as valid indicators of women’s participation in household decisions.

#### Statistical analysis

The NFHS data are structured hierarchically with level 1: women (*i*) nested within level 2: clusters (*j*), level 3: districts (*k*), and level 4: states (*l*). Leveraging this nested framework, we applied a four-level logistic regression model with Markov Chain Monte Carlo (MCMC) methodology to estimate the prevalence of women’s participation in decision-making across each district in India for the years 2016 and 2021. MCMC methods operate within a Bayesian framework, allowing posterior distributions for all model parameters and robust uncertainty quantification via 95% credible intervals [32, 33]. We first used first-order marginal quasi-likelihood (MQL1) to obtain starting values, followed by MCMC for final inference. The model is specified as below:

$$\:logit\:\left({Y}_{ijkl}\right)=\:{\beta\:}_{0}+\left({u}_{0jkl}+\:{v}_{0kl}+\:{f}_{0l}\right)$$

For the outcome in each survey round, $\:{\beta\:}_{0}$ represents the constant, and $\:{u}_{0jkl}\:,\:\:{v}_{0kl}\:and\:{f}_{0l}$ are the residual differentials for clusters *j*, districts *k*, and states *l*, respectively. Residuals were assumed to follow a normal distribution with a mean of 0 and a variance of $\:{u}_{0jkl}$ *~ N*(0, $\:{{\sigma\:}^{2}}_{u0}$), $\:{v}_{0kl}$ *~ N*(0, $\:{{\sigma\:}^{2}}_{v0}$), and $\:{f}_{0l}$ *~ N*(0, $\:{{\sigma\:}^{2}}_{f0}$). Specifically, the term $\:{{\sigma\:}^{2}}_{u0}$ captures the cluster-level variance within districts; $\:{{\sigma\:}^{2}}_{v0}$ represents the district-level variance within states, and $\:{{\sigma\:}^{2}}_{f0}$ denotes the state-level variance. To assess the relative contribution of each geographic level, we calculated the variance partitioning coefficient (VPC) by dividing the variance at each level by the sum of total geographic variance: $\:(\frac{{{{\upsigma\:}}^{2}}_{z}}{{{{\upsigma\:}}^{2}}_{u0}\:+\:{{{\upsigma\:}}^{2}}_{v0}\:+\:{{{\upsigma\:}}^{2}}_{f0}}$) × 100. Due to the binary nature of the outcome, the individual-level variance was fixed and not estimated.

To ensure convergence and sufficient mixing, we set a burn-in period of 1,000 cycles and monitored 25,000 MCMC iterations [32, 34]. Convergence was assessed by inspecting MCMC chain diagnostics and plots as well as by monitoring the effective sample size (ESS). Nearly all parameters across models achieved ESS values above 250, indicating reliable estimation. The MCMC estimates of residuals were obtained using the *runmlwin* command in Stata 18 [35]. These residuals were then used in the equation to generate precision-weighted cluster-level predicted prevalence:

$$\exp\left[\beta_0+\left(u_{\mathit0\mathit j\mathit k\mathit l}+v_{\mathit0kl}+f_{\mathit0l}+\right)\right]/\left[1+\exp\left[\beta_{\mathit0}+\left(u_{\mathit0jkl}+v_{\mathit0kl}+f_{\mathit0l}+\right)\right]\right]$$

Cluster-level prevalence was then averaged to derive district-level prevalence.

For the maps, we utilized the district averages from 2016 to establish decile thresholds. These thresholds were employed for the 2021 district averages to illustrate the variation in the prevalence of the outcome over time for each district. We utilized absolute increase values to establish seven categories of temporal change: substantial decrease (≤−10.00%); moderate decrease (−9.99% to −5.00%), small decrease (−4.99% to −2.50); no change (−2.49% to 2.49%); small increase (2.50% to 4.99%); moderate increase (5.00% to 9.99%); substantial increase (≥ 10.00%).

### Ethics statement

The Institutional Review Board of the International Institute for Population Studies approved the data collection for both rounds of the NFHS included in this study. This project does not meet the regulatory criteria for human subjects research as defined by the Harvard Longwood campus, so rendering it exempt from review.

### Role of the funding source

This work was supported by the Bill & Melinda Gates Foundation, INV-002992. The funder had no role in design and conduct of the study; collection, management, analysis, and interpretation of the data; preparation, review, or approval of the manuscript; and decision to submit the manuscript for publication.

## Results

### Women’s participation in decision-making

When sampling weights provided in the NFHS datasets were applied, 83.96% of women in 2016 reported participating in decision-making in at least one domain, increasing to 88.70% by 2021 **(**Table 1**)**. Similar levels of prevalence and upward trends were observed across all three domains. Given this consistency and the primary objective of capturing women’s overall agency within the household we focus on participation in any of the three domains as the main outcome throughout the results. However, for comparative purposes, domain-specific analyses are additionally presented in Supplementary Materials. Among both urban and rural women, the patterns of women’s participation in decision-making remained similar (Supplementary Tables 1–2).

**Table 1:** Sample size (N) and weighted percentage of women’s participation in household decision-making in India, 2016–2021

Variables	2021		2016	
	*N*	%	*N*	%
Total	76,910	100.00	86,694	100.00
Women’s participation in decision-making ^a^	68,333	88.70	73,344	83.96
Women’s healthcare				
Women alone or with husband/partner	62,711	81.09	65,580	74.52
Husband/partner alone	12,900	17.03	18,637	22.61
Someone else	861	1.25	1529	1.74
Other	438	0.63	948	1.14
Large household purchases				
Women alone or with husband/partner	61,257	79.51	64,184	73.37
Husband/partner alone	12,872	16.56	17,970	21.41
Someone else	2069	2.91	3182	3.59
Other	712	1.01	1358	1.63
Visits to family or relatives				
Women alone or with husband/partner	62,853	81.07	65,280	74.61
Husband/partner alone	12,094	16.15	17,719	21.20
Someone else	1452	2.05	2581	2.88
Other	511	0.74	1114	1.32

^a^Women were classified as participating in household decision-making if they reported making decisions either alone or jointly with their husband/partner in any of the three domains

In 2016, clusters accounted for the largest proportion of the total geographic variance in women’s participation in decision-making, followed by states and districts in India **(**Table 2**)**. This pattern persisted in 2021, with a slight increase in the proportion of variance attributed to clusters and a decrease in the contribution of states. A similar distribution was observed in urban and rural India (Supplementary Tables 3–4).

**Table 2:** Four-level variance component model for women’s participation in household decision-making in India, 2016–2021

	2021		2016	
	Variance estimate (95% CI)	VPC (%)	Variance estimate (95% CI)	VPC (%)
State	0.52 (0.29–0.90)	25.87	0.54 (0.30–0.89)	32.80
District	0.20 (0.15–0.25)	9.98	0.16 (0.13–0.19)	9.78
Cluster	1.28 (1.18–1.39)	64.14	0.94 (0.87–1.01)	57.41

*VPC * Variance Partitioning Coefficient, *CI * Confidence Intervals

### District-level variation in women’s participation in decision-making

**Fig. 1:**
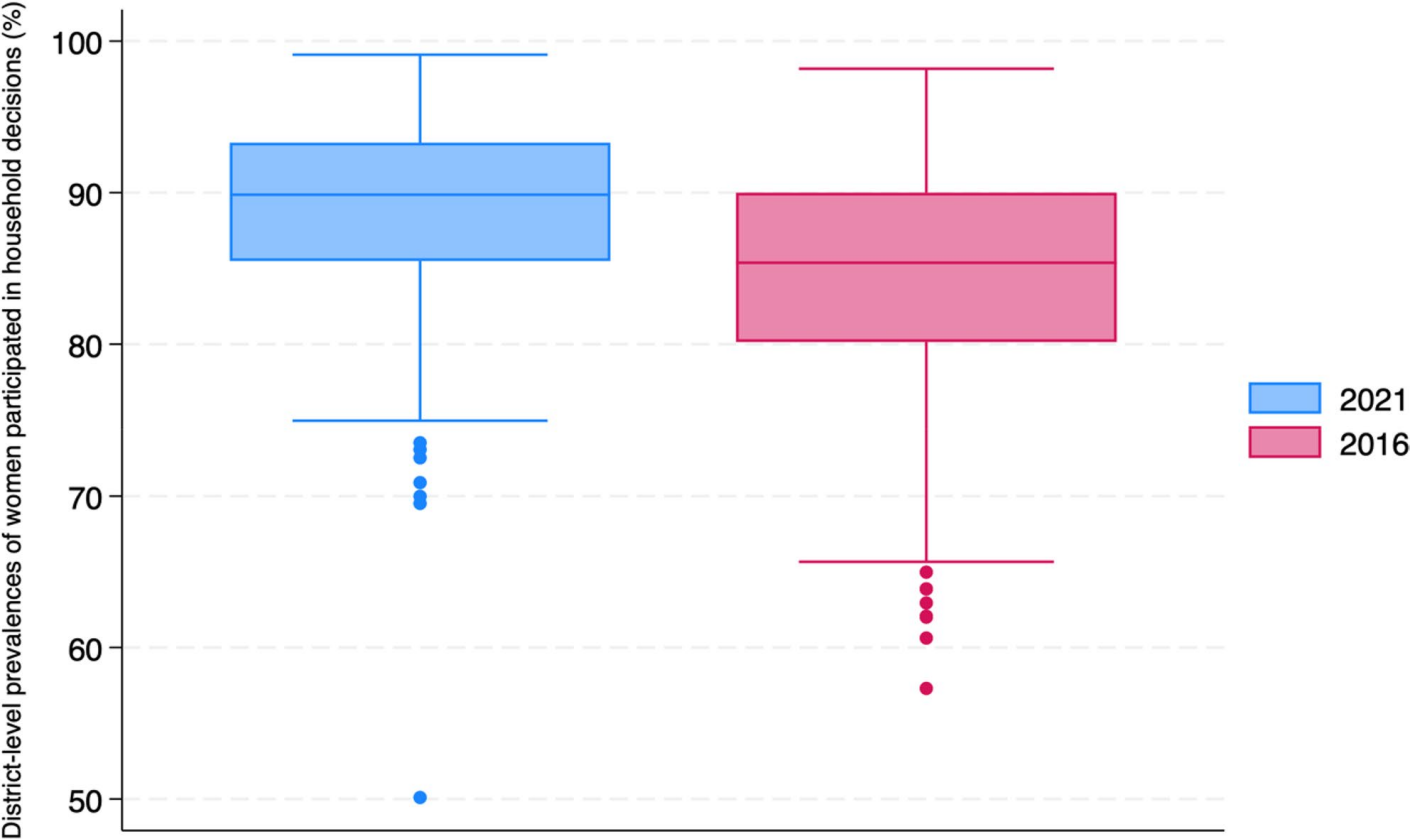
District-level prevalence of women’s participation in household decision-making in India, 2016–2021. Note. The upper and lower whiskers represent minimum and maximum values, respectively. The upper outline of the box depicts the 75th percentile and the lower outline the 25th percentile. The solid line within the box shows the median (50th percentile)

**Fig. 2:**
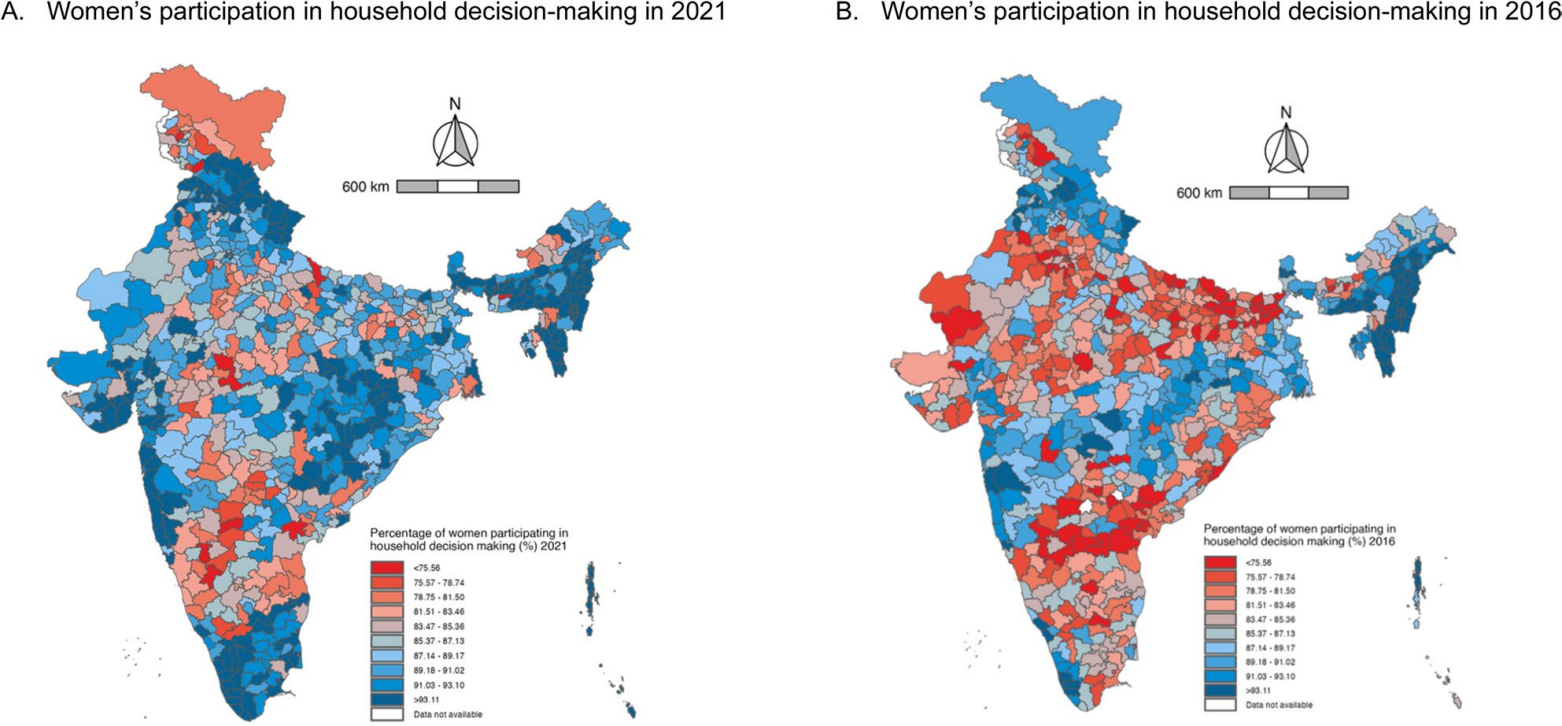
Maps of India illustrating the district-level prevalence of women’s participation in household decision-making, 2016–2021. Note. The decile thresholds were based on the 2016 district prevalence to illustrate the variation in the prevalence of the outcome over time for each district

Using cluster-level prevalence estimated through MCMC methods, the median district prevalence of women’s participation in decision-making was 85.38% (95% CI 84.79–85.95) in 2016 **(**Fig. 1**)**. District-level prevalence ranged from 57.30% (95% CI 49.43–64.61) to 98.17% (95% CI 96.59–99.22), with an interquartile range (IQR) of 9.85% points. Districts with relatively low levels of participation in decision-making (< 80%) were distributed across a broad geographic range, including northern states such as Uttar Pradesh (28 districts) and Haryana (12 districts), eastern states such as Bihar (26 districts), and southern states such as Karnataka (14 districts) and Telangana (12 districts) in 2016 (Fig. 2, Supplementary Table 5).

By 2021, the median increased to 89.87% (95% CI 89.31–90.36), with district-level prevalence ranging from 50.10% (95% CI 41.32–58.65) to 99.10% (95% CI 97.73–99.77) and an IQR of 7.81% points **(**Fig. 1**)**. Districts with relatively low levels of participation in decision-making (< 80%) were also dispersed across the country in 2021, including southern states such as Karnataka (9 districts) and Andhra Pradesh (4 districts), northern states such as Jammu & Kashmir (5 districts), and central states such as Madhya Pradesh (5 districts) in 2021 (Fig. 2, Supplementary Table 5). Similar geographic patterns were observed in both urban and rural areas (Supplementary Figs. 3–4, Supplementary Tables 6–7).

Domain-specific analyses of women’s decision-making revealed geographic patterns broadly consistent with those observed for participation in any domain (Supplementary Figs. 5–7). The only notable deviation was observed in the healthcare domain, where northern regions already demonstrated higher participation rates in 2016 compared to the south, and this advantage persisted through 2021.

### District-level change in women’s participation in decision-making

**Fig. 3:**
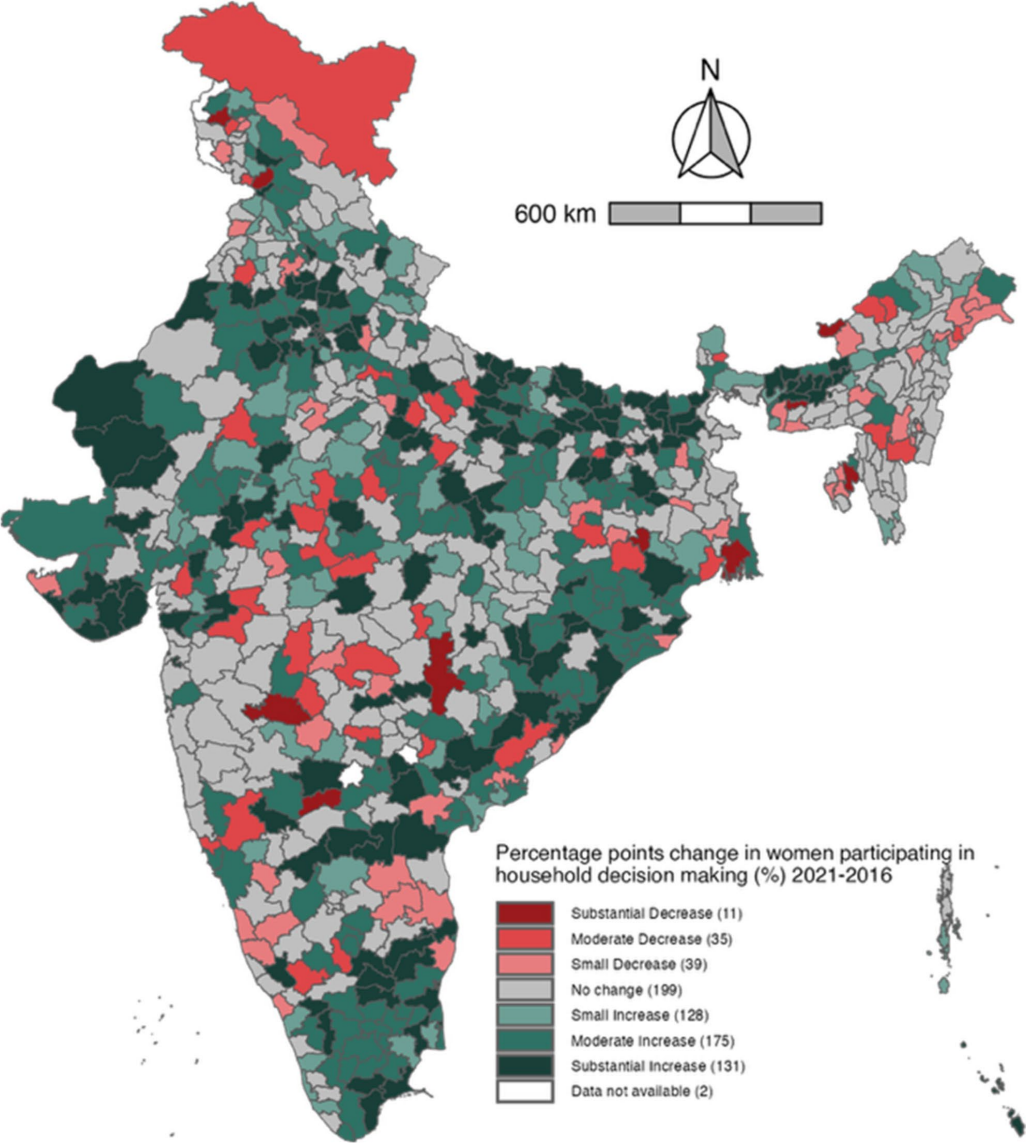
Map of India illustrating the absolute change in district-level prevalence of women’s participation in household decision-making, 2016–2021. Note. Absolute increase values were utilized to establish seven categories of temporal change: substantial decrease (≤−10.00%); moderate decrease (−9.99% to −5.00%), small decrease (−4.99% to −2.50); no change (−2.49% to 2.49%); small increase (2.50% to 4.99%); moderate increase (5.00% to 9.99%); substantial increase (≥10.00%)

Compared to 2016, there was a notable overall improvement in the prevalence of women’s participation in decision-making by 2021 in India **(**Fig. 3**)**. Specifically, 312 districts recorded an increase of more than 5% points, with 154 districts showing a moderate increase (5.00%–9.99%) and 158 districts demonstrating a substantial increase (≥ 10.00%). However, declines in participation (≤−2.5%) were observed in 100 districts, including 10 that experienced a substantial decrease (≤–10.00%). These declines occurred across a wide geographic range, such as in Maharashtra (9 districts), Arunachal Pradesh (9 districts), Uttar Pradesh (8 districts), Karnataka (8 districts), Madhya Pradesh (7 districts), Jammu & Kashmir (7 districts), and Andhra Pradesh (7 districts).

Similar spatial patterns were evident in both urban and rural areas. A moderate or substantial increase (≥ 5.00%) was observed in 283 districts in urban areas and 291 districts in rural areas by 2021. Conversely, declines in prevalence were observed in 112 districts in urban areas and 111 districts in rural areas (Supplementary Figs. 3 and 4).

In domain-specific analyses, district-level changes between 2016 and 2021 were broadly consistent with the overall pattern observed for participation in any domain, with increases and declines scattered across a wide geographic range. (Supplementary Figs. 5–7).

## Discussion

Our findings demonstrate a clear upward trend in women’s participation in household decision-making across India between 2016 and 2021, with notable improvements observed across all three decision-making domains: women’s healthcare, large household purchases, and visits to family or relatives. The increase was evident in both urban and rural areas, with urban areas exhibiting slightly higher levels of participation. While this overall trajectory signals progress, our district-level analysis reveals considerable geographic heterogeneity that has remained largely overlooked in prior state-level research conducted in India [25–27].In 2016, districts with relatively low decision-making participation (< 80%) were geographically dispersed across the country, and by 2021, both improvements and declines were dispersed rather than concentrated in specific regions. Drawing on Kishor’s (2000) framework which emphasizes the role of *settings* – the social and cultural environment in which women live – our findings underscore the importance of place-based influences in shaping women’s agency. Districts varied not only in baseline prevalence but also in the direction and magnitude of change over time, suggesting that structural and normative contexts, often clustered geographically, continue to condition women’s empowerment outcomes. This spatial variation points to the need of tailoring interventions to local social-cultural conditions rather than applying uniform national solutions.

Our findings also reveal that higher levels of women’s decision-making power do not necessarily correspond with higher levels of economic development, suggesting that broader regional and cultural contexts may exert a stronger influence than development alone. For instance, districts in northern India with over 90% of women reporting participation in decision-making do not uniformly exhibit the same level of economic development. For example, Serchhip and Aizawl in Mizoram both demonstrate high levels of women’s decision-making participation (>90%), despite marked differences in their economic foundations – Serchhip being primarily agriculture-oriented, while Aizawl’s economy is more urbanized and driven by tourism and services [36]. Conversely, districts such as Guntur and Srikakulam in Andhra Pradesh demonstrate that higher levels of economic development do not necessarily translate into greater women’s agency. Despite Guntur having a significantly higher Net District Domestic Product in 2020–2021 (Rs 7,881,600 lakh) [37] compared to Srikakulam (Rs 3,532,600 lakh) [38], women’s participation in decision-making in 2021 was lower (79%) than in Srikakulam (89%). This contrast underscores that cultural and geographic contexts can outweigh economic development in shaping women’s decision-making power.

While overall levels of women’s decision-making participation have improved in 2021 compared to 2016, considerable inter-district disparities persist, with many districts continuing to lag behind despite national progress. Notably, approximately 14% of districts (100 out of 718) experienced a decline in women’s participation in decision-making by 2021, suggesting a concerning trend of stagnation or regression in women’s empowerment progress. Moreover, many districts that were already underperforming in 2016 did not show accelerated growth, indicating a failure to bridge the gap. These patterns underscore limitations in the current policy efforts aimed at enhancing women’s empowerment. Furthermore, because the districts experiencing declines were geographically widespread, future research should move beyond state-level analyses and focus on district-specific investigations to uncover the underlying mechanisms and contextual drivers of this variation.

There is a need for more critical evaluation of these policies, particularly in terms of implementation and targeting, to identify areas where they may be falling short. Recent evidence on policy interventions, such as reforms to inheritance laws and the dowry system, has shown positive impacts on women’s labor force participation and educational attainment [39–41]. Mandated political representation of women has also been linked to improvements in girls’ education, reductions in child marriage, and increased participation in entrepreneurship [42, 43]. However, several studies highlight that resources intended for women are often appropriated by their husbands or redirected to other household enterprises, limiting the effectiveness of such interventions [44]. Integrating theoretical frameworks that elucidate the complex familial, cultural, and structural factors shaping women’s agency could enhance the design and evaluation of future interventions.

At the same time, our study underlines the potential to identify exceptional districts – those that outperform their surrounding areas despite being situated in regions that generally perform poorly in women’s decision-making. For example, Warangal district in Telangana includes Darmaram and Gangadevi Palli, two villages widely recognized for their successful local governance model that fosters women’s participation in civic life and decision-making [45]. These districts can serve as valuable cases for examining successful policy implementation or innovative strategies that may be driving progress. Conversely, underperforming districts located in otherwise better-performing regions can help reveal missing enablers or persistent barriers. Together, these contrasting cases can offer critical insights into the contextual drivers of women’s decision-making and inform the design of more targeted and effective interventions.

While our analysis highlights important district-level geographic patterns, it is important to note that the greatest variation in women’s participation in decision-making was observed at the cluster level, as indicated by our variance partitioning coefficients. This suggests that decision-making power is shaped by highly localized dynamics, possibly operating at the household or community level [18]. Unlike other empowerment factors, such as women’s educational attainment, that may be influenced more heavily by state-level infrastructural factors, decision-making often reflects the influence of localized household dynamics [18]. For example, spousal relationships and family arrangements, including co-residence with parents-in-law, may largely shape the household decision-making process [46, 47]. Recognizing this, many empowerment initiatives in India have been implemented through community-based mobilization efforts. For instance, local Self-Help Groups (SHGs) [48] and microfinance programs [49] have been shown to strengthen women’s decision-making power by fostering peer support, financial autonomy, and collective action within communities. Although our study focused on district-level prevalence and trends, the largest variation at the cluster level underscores the need for future qualitative investigations and case studies to explore how decision-making power is negotiated within households and smaller communities. Such localized investigations could illuminate the household-level and micro-social mechanisms that aggregate to shape broader patterns of women’s decision-making.

The findings of this study should be interpreted in light of several limitations. First, our analysis is limited to currently married women in India, restricting the generalizability of the findings to the broader population of women, particularly those outside marital unions who may experience different forms of agency and household dynamics. Moreover, caution is warranted in extrapolating these findings to sociocultural contexts outside of India, where marriage patterns, gender norms, and decision-making structures may differ substantially. Second, the study relies on self-reported data, which introduces a degree of subjectivity. In contexts where women are socioeconomically disempowered or culturally marginalized, even minimal involvement may be perceived as meaningful participation. Moreover, responses may be influenced by social desirability bias, whereby women overreport participation in decision-making to align with perceived normative expectations. Thus, the perceived meaning and reliability of self-reported decision-making measures should be interpreted in light of local social and cultural contexts, as emphasized in prior work [6]. Third, while our primary outcome combined both independent and joint decision-making into a single measure, we acknowledge that these two types of decision-making may operate through distinct pathways and carry different implications. For instance, women’s independent decision-making may sometimes reflect a gendered division of labor rather than genuine agency, whereas joint decision-making can signal more equitable spousal collaboration and shared power. Future studies should disaggregate these forms to examine their unique patterns and consequences, thereby advancing a more nuanced understanding of women’s agency within households. Finally, as our study aimed to map the crude geographic variation in women’s decision-making, we intentionally did not adjust for individual-level factors such as women’s education or employment. Future research should consider how these characteristics shape women’s decision-making patterns across regions.

Despite these limitations, our study offers valuable insights into the spatial heterogeneity of women’s decision-making power in India. While national trends indicate overall improvements, notable disparities across and within districts highlight the limitations of one-size-fits-all policy approaches. These patterns imply that localized dynamics, including household negotiation patterns, community norms, and region-specific cultural contexts, may contribute to shaping women’s agency. Future research should further explore the micro-level dynamics of household negotiation and community norms, as understanding these granular drivers is essential to advancing women’s decision-making power.

## Conclusion

In conclusion, this study highlights the substantial geographic variation in women’s participation in household decision-making across India, even amidst national progress between 2016 and 2021. Our findings reveal that regional and cultural contexts, rather than economic development alone, may play a decisive role in shaping women’s decision-making. The persistence of inter-district inequality, coupled with a decline in participation in 100 districts, points to the need for more nuanced and locally responsive policy strategies. Notably, districts that outperform their regional peers offer promising opportunities for evidence-based modeling of effective intervention. Achieving meaningful and sustained progress in women’s empowerment will require policies that account for the social norms, family structures, and local power dynamics shaping women’s household decision-making.

## Supplementary Information

Supplementary Material 1

## Data Availability

The dataset analysed during the current study are available in the DHS website: [https://dhsprogram.com/data/available-datasets.cfm](https://dhsprogram.com/data/available-datasets.cfm).

## Abbreviations

AP: Andhra Pradesh
DHS: Demographic and Health Surveys
ESS: Effective sample size
IQR: Interquartile range
MCMC: Markov Chain Monte Carlo
NFHS: National Family Health Survey

## References

[1] Abreha SK, Zereyesus YA. Women’s empowerment and infant and child health status in sub-Saharan Africa: a systematic review. Matern Child Health J. 2021;25:95–106. 33226578 10.1007/s10995-020-03025-y PMC7822794

[2] Varkey P, Kureshi S, Lesnick T. Empowerment of women and its association with the health of the community. J Womens Health. 2010;19(1):71–6. 10.1089/jwh.2009.1444 20088661

[3] United Nations. Transforming our world: the 2030 Agenda for Sustainable Development. 2015. Available from: https://sdgs.un.org/2030agenda.

[4] Chi H, Eom Y-J, Jung S, Kim J, Jeong J, Kim R. Maternal decision-making power and care-seeking behaviors for acutely ill children: a multilevel analysis of 33 sub-Saharan African countries. Am J Trop Med Hyg. 2024. 10.4269/ajtmh.23-0511. 38190745 10.4269/ajtmh.23-0511 PMC10859818

[5] Eom Y-J, Chi H, Jung S, Kim J, Jeong J, Subramanian SV, et al. Women’s empowerment and child anthropometric failures across 28 sub-Saharan African countries: a cross-level interaction by Gender Inequality Index. SSM - Population Health. 2024;26:101651. 38524893 10.1016/j.ssmph.2024.101651 PMC10958109

[6] Eom Y-J, Chi H, Bhatia A, Lee H-Y, Subramanian S, Kim R. Individual-and community-based women’s empowerment and complete use of maternal healthcare services: a multilevel analysis of 34 sub-Saharan African countries. Soc Sci Med. 2025;370:117816. 10.1016/j.socscimed.2025.117816 39999578

[7] Berti PR, Sohani S, Costa Ed, Klaas N, Amendola L, Duron J. An adequacy evaluation of a maternal health intervention in rural Honduras: the impact of engagement of men and empowerment of women. Rev Panam Salud Publica. 2015;37:90–7. 25915013

[8] Jejeebhoy SJ, Sathar Z. Revisiting women’s empowerment and contraception. Popul Dev Rev. 2024;50(S2):597–623.

[9] Mainuddin A, Begum HA, Rawal LB, Islam A, Islam SS. Women empowerment and its relation with health seeking behavior in Bangladesh. J Fam Reprod Health. 2015;9(2):65. PMC4500817 26175761

[10] Firdaush S, Das P. Intimate partner violence and its associated factors: a multidimensional analysis in the context of India. J Asian Afr Stud. 2025;60(2):661–76.

[11] Atake E-H, Gnakou Ali P. Women’s empowerment and fertility preferences in high fertility countries in Sub-Saharan Africa. BMC Womens Health. 2019;19(1):1–14. 30953494 10.1186/s12905-019-0747-9 PMC6451210

[12] Prata N, Fraser A, Huchko MJ, Gipson JD, Withers M, Lewis S, et al. Women’s empowerment and family planning: a review of the literature. J Biosoc Sci. 2017;49(6):713–43. 28069078 10.1017/S0021932016000663 PMC5503800

[13] Upadhyay UD, Gipson JD, Withers M, Lewis S, Ciaraldi EJ, Fraser A, et al. Women’s empowerment and fertility: a review of the literature. Soc Sci Med. 2014;115:111–20. 24955875 10.1016/j.socscimed.2014.06.014 PMC4096045

[14] Subburathinam P, et al. Inside government’s ₹3 trillion push to empower women at work. The Economic Times. 2022. Available from: https://economictimes.indiatimes.com/jobs/hr-policies-trends/inside-governments-3-trillion-push-to-empower-women-at-work/articleshow/112678608.cms?from=mdr.

[15] Government of India - Press Information Bureau. NARI SHAKTI: From Women Development to Women led Development. 2024. Available from: https://www.pib.gov.in/PressNoteDetails.aspx?NoteId=151861&ModuleId=3®=3&lang=1.

[16] Ministry of Women and Child Development. GOI initiatives for Women Empowerment 2022 [Available from: https://www.pib.gov.in/PressReleasePage.aspx?PRID=1845382.

[17] International Institute for Population Sciences I. National family health survey (NFHS-5), 2019–21: India. India: National Family Health Survey; 2021.

[18] Eom Y-J, Subramanian SV, Kim R. Geographic variation in women’s empowerment: a multilevel analysis of India’s National Family Health Survey 2021. J Glob Health. 2025. 10.7189/jogh.15.04159. 40576126 10.7189/jogh.15.04159 PMC12203628

[19] Harper C, Watson C, Bantebya GK, Ghimire A, George R. Historical lessons on gender norm change, with case studies from Uganda and Nepal. London: Overseas Development Institute(Advancing Learning and Innovation on Gender Norms); 2020.

[20] Banks J, Sweeney S, Meiring W. The geography of women’s empowerment in West Africa. Spat Demography. 2022;10(2):387–412. 10.1007/s40980-021-00099-2 PMC9611597 36311385

[21] Rogers EM, Singhal A, Quinlan MM. Diffusion of innovations. An integrated approach to communication theory and research. 2nd ed. Routledge; 2014. p. 432–4.

[22] Kishor S. Empowerment of women in Egypt and links to the survival and health of their infants. New York: Oxford University Press; 2000.

[23] Evans A. How cities erode gender inequality: a new theory and evidence from Cambodia. Gend Soc. 2019;33(6):961–84.

[24] Scarborough WJ, Sin R. Gendered places: the dimensions of local gender norms across the United States. Gend Soc. 2020;34(5):705–35.

[25] Paul T, Karmakar S. Domestic violence against women in India: does empowerment matter? J Asian Afr Stud. 2024;59(5):1676–97.

[26] Kumar S, Mondal S. Empowerment of women from the experience of Indian states: a reflection of NFHS-5. GeoJournal. 2024;89(2):64.

[27] Kumari S, Siotra V. Indian females in the twenty-first century: how they have fared? An analysis using Geospatial techniques. GeoJournal. 2023;88(4):4279–95. 10.1007/s10708-023-10865-y PMC10044063 38625116

[28] Rajpal S, Ko S, Leckie G, Jain D, Blossom JC, Kim R, et al. India policy insights: estimates of population health and social determinants indicators across policy units. Sci Data. 2025;12(1):1592. 41028730 10.1038/s41597-025-05923-8 PMC12484729

[29] Kabeer N. Resources, agency, achievements: reflections on the measurement of women’s empowerment. Dev Change. 1999;30(3):435–64.

[30] Ewerling RA, Victora CG, Hellwig F, Coll CV, Barros AJ. SWPER Global: A survey-based women’s empowerment index expanded from Africa to all low-and middle-income countries. J Global Health. 2020;10(2). 10.7189/jogh.10.020434 PMC7699005 33274055

[31] Miedema SS, Haardörfer R, Girard AW, Yount KM. Women’s empowerment in East Africa: development of a cross-country comparable measure. World Dev. 2018;110:453–64.

[32] Browne WJ. MCMC Estimation in MLwiN. United Kingdom: Centre for Multilevel Modelling, University of Bristol; 2017.

[33] Rasbash J, Steele F, Browne W, Goldstein H, Charlton C. A user's guide to MLwin. United Kingdom: Centre for Multilevel Modeling, University of Bristol; 2015.

[34] Kass RE, Carlin BP, Gelman A, Neal RM. Markov chain Monte Carlo in practice: a roundtable discussion. Am Stat. 1998;52(2):93–100.

[35] Leckie GB, Charlton CM. Runmlwin: a program to run the MLwiN multilevel modeling software from within Stata. J Stat Softw. 2013;52(11).

[36] Directorate of Economics and Statistics - Governement of Mizoram. Statistical Handbook Mizoram. Mizoram: Standard Laser Print; 2020.

[37] Datanet India. District Level Information of Guntur (Andhra Pradesh) [Available from: https://www.indiastatdistricts.com/andhrapradesh/guntur-district.

[38] Datanet India. District Level Information of Srikakulam (Andhra Pradesh) [Available from: https://www.indiastatdistricts.com/andhrapradesh/srikakulam-district.

[39] Calvi R, Keskar A. ‘Til Dowry Do Us Part: Bargaining and Violence in Indian Families. CEPR Discussion Papers. 2021:1–37.

[40] Heath R, Tan X. Intrahousehold bargaining, female autonomy, and labor supply: theory and evidence from India. J Eur Econ Assoc. 2020;18(4):1928–68.

[41] Roy S. Empowering women? Inheritance rights, female education and dowry payments in India. J Dev Econ. 2015;114:233–51.

[42] Beaman L, Duflo E, Pande R, Topalova P. Female leadership raises aspirations and educational attainment for girls: a policy experiment in India. Science. 2012;335(6068):582–6. 22245740 10.1126/science.1212382 PMC3394179

[43] Castilla C. Political role models and child marriage in India. Rev Dev Econ. 2018;22(4):1409–31.

[44] Bernhardt A, Field E, Pande R, Rigol N. Household matters: revisiting the returns to capital among female microentrepreneurs. Am Economic Review: Insights. 2019;1(2):141–60.

[45] Kanakalatha V. The socio-economic empowerment of women through self help groups - an empirical study. J Bus Manage. 2017;19(7):35–45.

[46] McClain L, Brown SL. The roles of fathers’ involvement and coparenting in relationship quality among cohabiting and married parents. Sex Roles. 2017;76(5–6):334–45. 30555203 PMC6294450

[47] Jejeebhoy SJ, Sathar ZA. Women’s autonomy in India and Pakistan: the influence of religion and region. Popul Dev Rev. 2001;27(4):687–712.

[48] Zavaleta Cheek J, Corbett PE. Public decision making by women’s self-help groups and its contributions to women’s empowerment: evidence from West Bengal, India. World Development Perspectives. 2024;33:100549.

[49] Lavoori V, Paramanik RN. Microfinance impact on women’s decision making: a case study of Andhra Pradesh. J Glob Entrepreneurship Res. 2014;4(1):11.

