# Supplementary material for: Assessing geographic variation in women’s decision-making power across 720 districts in India, 2016–2021
Source: BMC Womens Health. 2025 Dec 18;26:74. doi: 10.1186/s12905-025-04194-0 (PMC12874934; doi:10.1186/s12905-025-04194-0)
Supplement: Supplementary file 1 — Supplementary Material 1 [file 12905_2025_4194_MOESM1_ESM.pdf]

Supplementary Table 1. Sample size (N) and weighted percentage of urban women's participation in household decision-making in India, 2016-2021

**Variables**	**2021**		**2016**	
	N	%	N	%
**Total**	18360	100.00	25294	100.00
**Women's participation in decision-making**[a]	16682	91.01	21933	85.83
**Women's healthcare**				
Women alone or with husband/partner	15298	83.23	19580	76.08
Husband/partner alone	2787	15.02	5116	21.72
Someone else	182	1.10	346	1.27
Other	93	0.65	252	0.93
**Large household purchases**				
Women alone or with husband/partner	15052	82.51	19559	76.55
Husband/partner alone	2695	13.96	4550	19.12
Someone else	465	2.62	798	2.85
Other	148	0.92	387	1.49
**Visits to family or relatives**				
Women alone or with husband/partner	15442	83.81	19951	78.18
Husband/partner alone	2510	13.83	4453	18.56
Someone else	303	1.73	566	2.06
Other	105	0.62	324	1.20

a. Women were classified as participating in household decision-making if they reported making decisions either alone or jointly with their husband/partner in any of the three domains.

Supplementary Table 2. Sample size (N) and weighted percentage of rural women's participation in household decision-making in India, 2016-2021

Variables	2021		2016	
	N	%	N	%
**Total**	58550	100.00	61400	100.00
**Women's participation in decision-making[a]**	51651	87.66	51411	82.96
**Women's healthcare**				
Women alone or with husband/partner	47413	80.12	46000	73.69
Husband/partner alone	10113	17.93	13521	23.07
Someone else	679	1.32	1183	1.99
Other	345	0.62	696	1.25
**Large household purchases**				
Women alone or with husband/partner	46205	78.17	44625	71.67
Husband/partner alone	10177	17.73	13420	22.63
Someone else	1604	3.03	2384	3.99
Other	564	1.06	971	1.71
**Visits to family or relatives**				
Women alone or with husband/partner	47411	79.83	45329	72.70
Husband/partner alone	9584	17.19	13266	22.61
Someone else	1149	2.19	2015	3.31
Other	406	0.79	790	100.00

a. Women were classified as participating in household decision-making if they reported making decisions either alone or jointly with their husband/partner in any of the three domains.

Supplementary Table 3. Four-level variance component model for urban women's participation in household decision-making in in India, 2016-2021

	2021		2016	
	Variance estimate (95% CI)	VPC (%)	Variance estimate (95% CI)	VPC (%)
State	0.40 (0.19-0.73)	24.40	0.57 (0.30-0.99)	34.48
District	0.23 (0.11-0.37)	13.83	0.20 (0.12-0.29)	12.09
Cluster	1.02 (0.80-1.26)	61.76	0.89 (0.76-1.02)	53.43

Note. VPC – variance partitioning coefficient; CI – Confidence Intervals.

Supplementary Table 4. Four-level variance component model for rural women's participation in household decision-making in India, 2016-2021

	2021		2016	
	Variance estimate (95% CI)	VPC (%)	Variance estimate (95% CI)	VPC (%)
State	0.56 (0.29-1.05)	26.88	0.53 (0.29-0.95)	33.13
District	0.22 (0.16-0.28)	10.44	0.18 (0.14-0.23)	11.12
Cluster	1.30 (1.19-1.42)	62.68	0.90 (0.82-0.98)	55.75

Note. VPC – variance partitioning coefficient; CI – Confidence Intervals.

Supplementary Figure 1. District-level prevalence of urban women's participation in household decision-making in India, 2016-2021

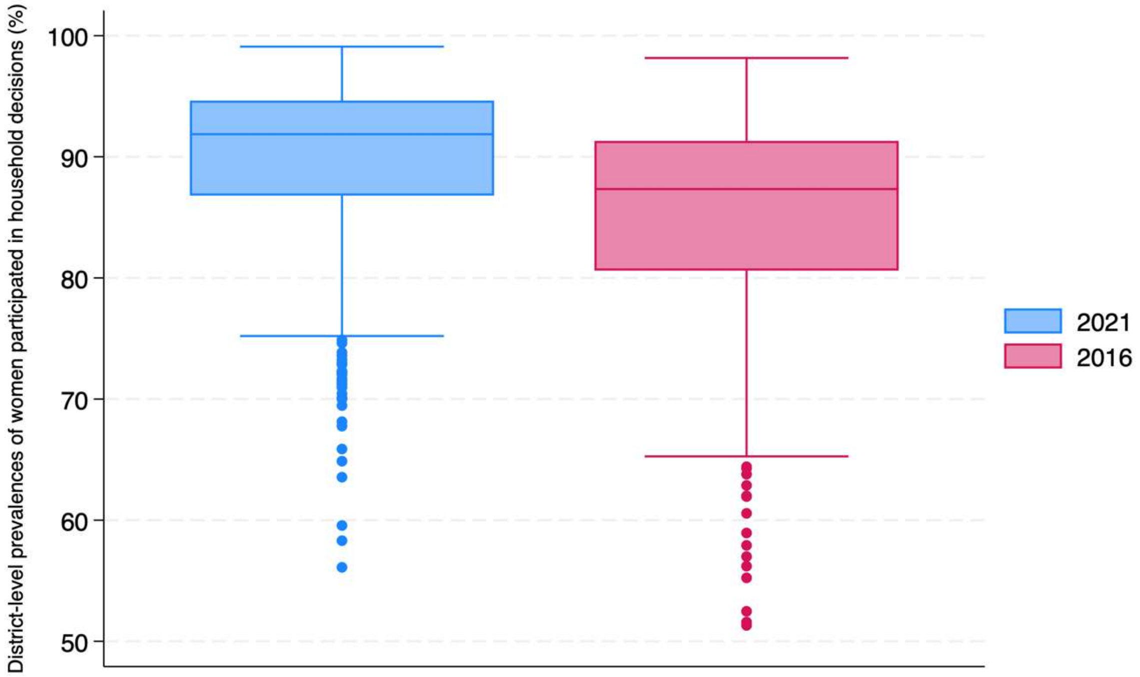

Note. The upper and lower whiskers represent minimum and maximum values respectively. The upper outline of the box depicts the 75th percentile and the lower outline the 25th percentile. The solid line within the box shows the median (50th percentile).

Supplementary Figure 2. District-level prevalence of rural women's participation in household decision-making in India, 2016-2021

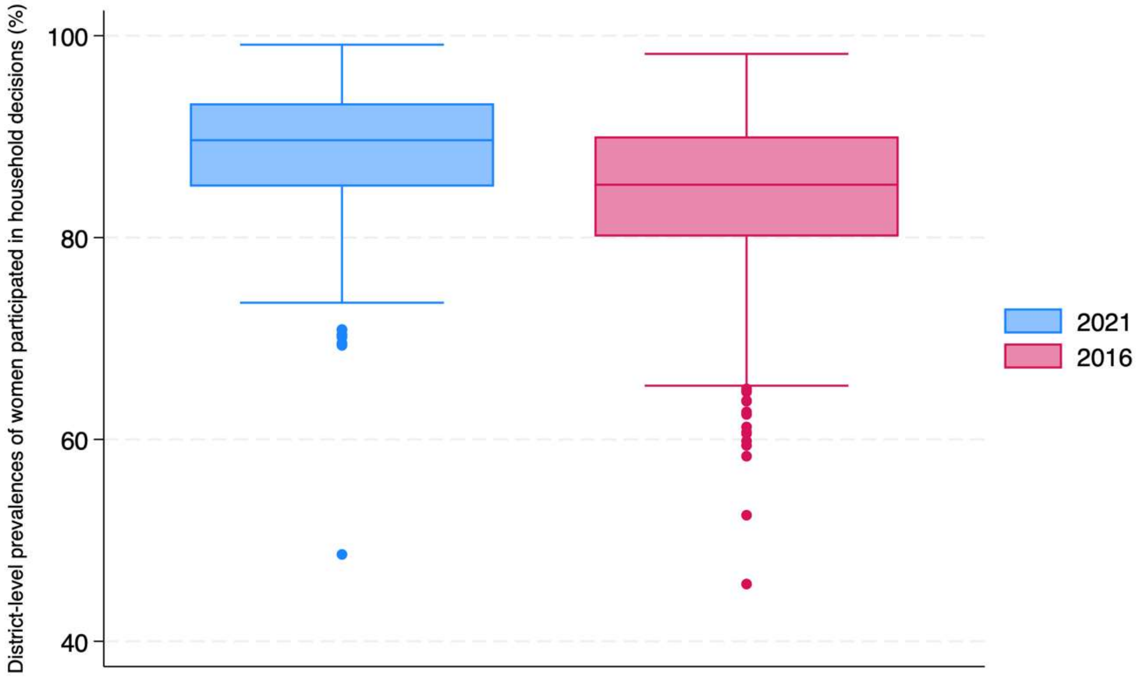

Note. The upper and lower whiskers represent minimum and maximum values respectively. The upper outline of the box depicts the 75th percentile and the lower outline the 25th percentile. The solid line within the box shows the median (50th percentile).

Supplementary Figure 3. Maps of India illustrating the district-level prevalence of urban women's participation in household decision-making, 2016-2021 and the absolute change in prevalence from 2016-2021

A. Women's participation in household decision-making in 2021

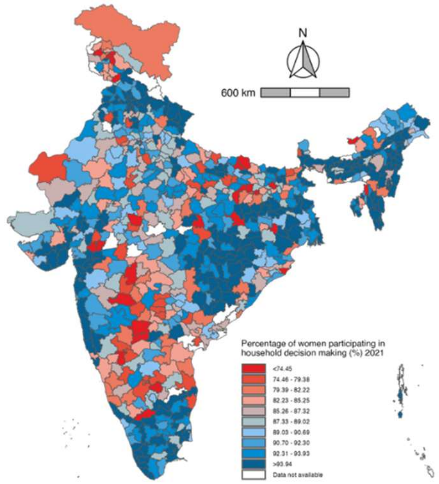

B. Women's participation in household decision-making in 2016

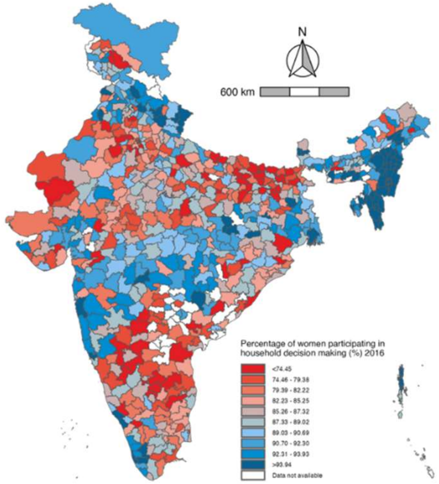

C. Women' participation in household decision-making change from 2016-2021

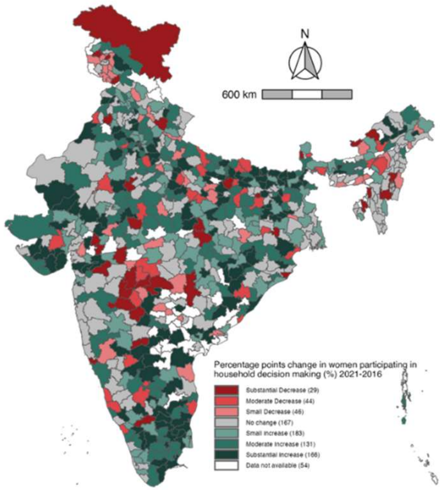

Note. The decile thresholds were based on the 2016 district prevalence to illustrate the variation in the prevalence of the outcome over time for each district. Absolute increase values were utilized to establish seven categories of temporal change: substantial decrease (≤-10.00%); moderate decrease (-9.99% to -5.00%), small decrease (-4.99% to -2.50); no change (-2.49% to 2.49%); small increase (2.50% to 4.99%); moderate increase (5.00% to 9.99%); substantial increase (≥10.00%).

Supplementary Figure 4. Maps of India illustrating the district-level prevalence of rural women's participation in household decision-making, 2016-2021 and the absolute change in prevalence from 2016-2021

A. Women's participation in household decision-making in 2021

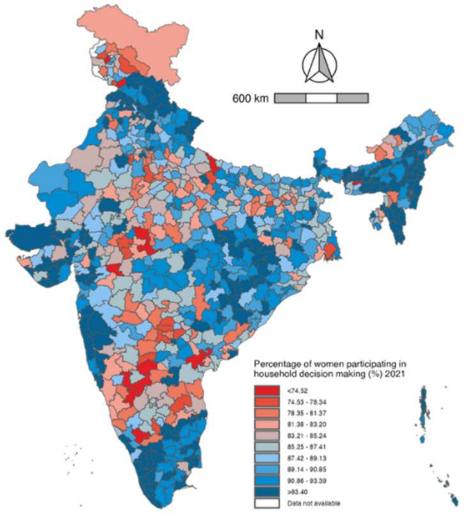

B. Women's participation in household decision-making in 2016

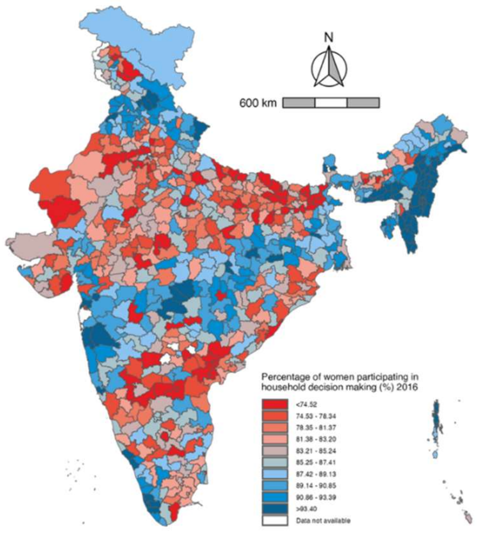

C. Women' participation in household decision-making change from 2016-2021

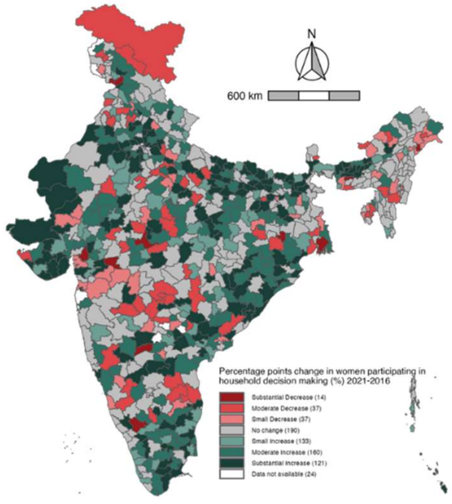

Note. The decile thresholds were based on the 2016 district prevalence to illustrate the variation in the prevalence of the outcome over time for each district. Absolute increase values were utilized to establish seven categories of temporal change: substantial decrease (≤-10.00%); moderate decrease (-9.99% to -5.00%), small decrease (-4.99% to -2.50); no change (-2.49% to 2.49%); small increase (2.50% to 4.99%); moderate increase (5.00% to 9.99%); substantial increase (≥10.00%).

Supplementary Figure 5. Maps of India illustrating the district-level percentage of women's participation in decisions on women's healthcare, 2016-2021 and the absolute change in prevalence from 2016 to 2021

A. Women participated in respondents' healthcare decisions in 2021

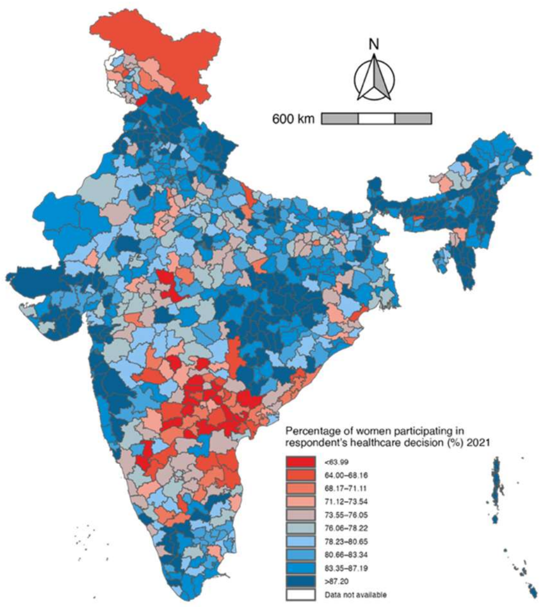

B. Women participated in respondents' healthcare decisions in 2016

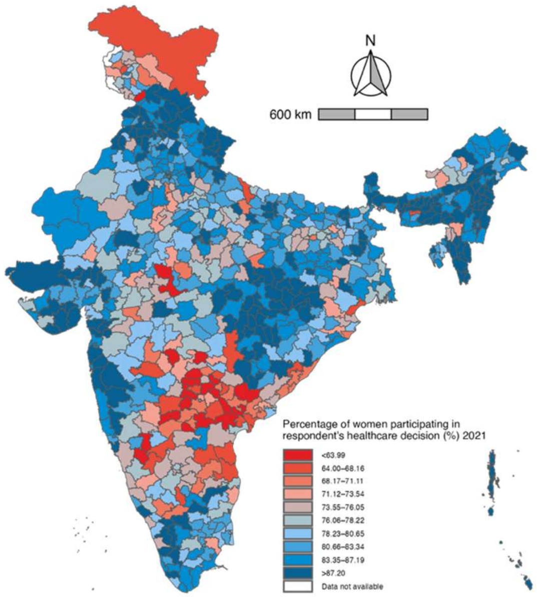

C. Women participated in respondents' healthcare decisions change from 2016-2021

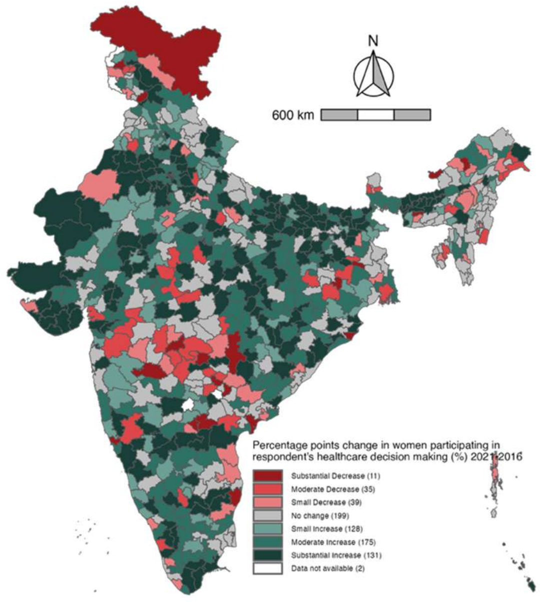

Supplementary Figure 6. Maps of India illustrating the district-level percentage of women's participation in decisions on large household purchases, 2016-2021 and the absolute change in prevalence from 2016 to 2021

A. Women participated in large-scale household purchase decisions in 2021

B. Women participated in large-scale household purchase decisions in 2016

C. Women participated in large-scale household purchase decisions change from 2016-2021

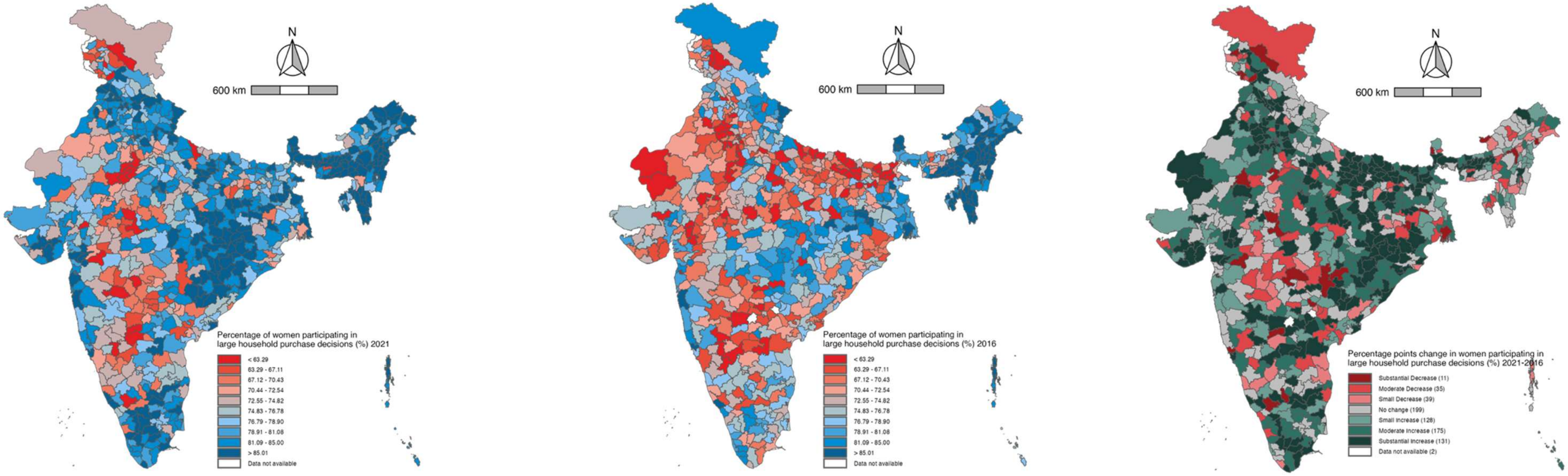

Supplementary Figure 7. Maps of India illustrating the district-level percentage of women's participation in decisions on visits to family or relatives, 2016-2021 and the absolute change in prevalence from 2016 to 2021

A. Women participated in decisions on visits to family and relatives in 2021

B. Women participated in decisions on visits to family and relatives in 2016

C. Women participated in decisions on visits to family and relatives change from 2016-2021

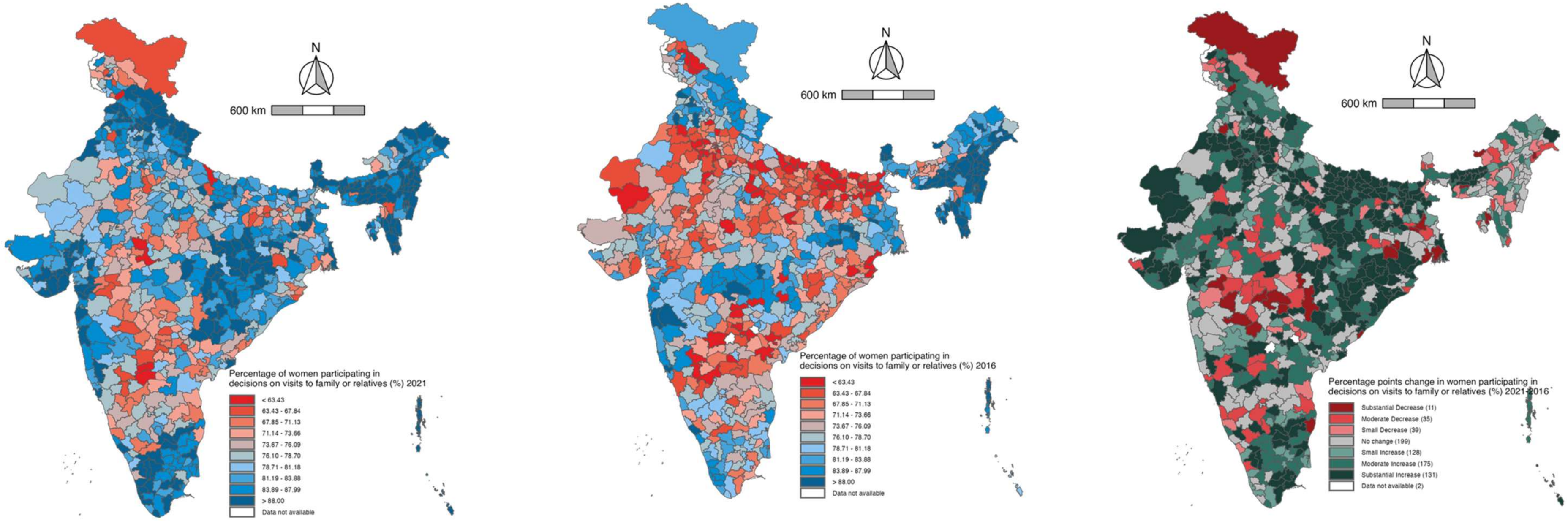

Supplementary Table 5. District-level prevalence and 95% credible intervals of women's participation in household decision-making in India, 2016-2021

			2021			2016		
**State**	**District**	**District ID**	**Mean**	**95% CI**		**Mean**	**95% CI**	
Andhra Pradesh	Alluri Sitharama Raju	2	80.67	56.54	95.90	87.44	76.91	94.73
Andhra Pradesh	Anakapalli	3	85.59	76.53	92.98	85.27	70.71	95.01
Andhra Pradesh	Ananthapuramu	4	85.94	78.41	92.08	81.90	74.40	88.27
Andhra Pradesh	Annamayya	5	79.78	72.37	86.93	82.60	73.71	89.71
Andhra Pradesh	Bapatla	6	86.77	77.94	93.76	80.75	71.16	88.50
Andhra Pradesh	Chittoor	7	82.26	72.07	91.20	83.98	74.08	91.85
Andhra Pradesh	Dr. B.R. Ambedkar Konaseema	8	93.62	85.47	98.28	84.19	67.81	94.62
Andhra Pradesh	East Godavari	9	83.13	75.41	89.69	86.64	78.88	92.72
Andhra Pradesh	Eluru	10	89.19	81.67	94.60	79.23	72.45	85.32
Andhra Pradesh	Guntur	11	79.26	70.57	86.98	73.90	62.83	83.65
Andhra Pradesh	Kakinada	12	87.94	79.38	94.34	79.21	68.92	87.76
Andhra Pradesh	Krishna	13	86.14	75.30	93.92	81.44	68.83	91.25
Andhra Pradesh	Kurnool	14	86.01	79.10	92.09	71.63	63.08	79.36
Andhra Pradesh	Nandyal	15	91.94	85.77	96.33	73.58	64.18	82.51
Andhra Pradesh	Ntr	16	79.27	68.79	87.71	79.14	69.17	87.72
Andhra Pradesh	Palnadu	17	70.89	56.49	82.97	75.56	65.99	84.42
Andhra Pradesh	Parvathipuram Manyam	18	88.36	78.72	94.96	78.65	66.00	89.24
Andhra Pradesh	Prakasam	19	85.13	77.01	91.88	73.98	63.44	83.37
Andhra Pradesh	Sri Potti Sriramulu Nellore	20	80.47	72.43	86.83	81.23	74.65	86.74
Andhra Pradesh	Sri Sathya Sai	21	82.98	74.07	90.27	81.47	68.43	91.73
Andhra Pradesh	Srikakulam	22	89.21	81.99	94.61	71.90	63.43	79.95
Andhra Pradesh	Tirupati	23	80.87	71.61	89.11	85.19	78.30	90.91
Andhra Pradesh	Visakhapatnam	24	81.66	70.92	90.13	86.36	78.74	92.12
Andhra Pradesh	Vizianagaram	25	89.49	83.33	94.09	77.32	69.23	84.15
Andhra Pradesh	West Godavari	26	85.55	75.72	93.27	80.89	68.00	91.17
Andhra Pradesh	Y.S.R.	27	82.49	75.47	88.36	85.40	79.01	90.58
West Bengal	Kolkata	29	94.91	90.91	97.75	92.75	88.12	96.30
Jammu & Kashmir	Kupwara	30	88.04	82.30	92.63	82.15	77.37	86.47
Jammu & Kashmir	Badgam	31	75.06	67.80	81.58	80.64	75.80	85.04
Ladakh	Leh	32	81.20	73.88	87.77	89.81	85.65	93.30

Jammu & Kashmir	Punch	33	84.13	77.64	89.92	86.31	82.53	89.75
Jammu & Kashmir	Rajouri	34	81.14	75.14	86.68	84.18	79.74	88.09
Jammu & Kashmir	Kathua	35	50.10	41.33	58.65	85.54	81.36	89.29
Jammu & Kashmir	Baramula	36	76.43	69.48	82.99	87.14	83.00	90.92
Jammu & Kashmir	Bandipore	37	80.07	73.22	86.40	76.92	71.68	81.65
Jammu & Kashmir	Srinagar	38	81.35	73.63	87.93	84.40	79.51	88.74
Jammu & Kashmir	Ganderbal	39	82.53	76.56	87.78	75.20	70.05	80.22
Jammu & Kashmir	Pulwama	40	86.93	80.91	91.92	91.85	88.18	94.95
Jammu & Kashmir	Shupiyan	41	90.00	84.52	94.26	91.20	87.53	94.29
Jammu & Kashmir	Anantnag	42	81.35	73.49	88.07	78.39	73.38	82.93
Jammu & Kashmir	Kulgam	43	86.88	80.45	92.08	88.81	84.76	92.29
Jammu & Kashmir	Doda	44	88.58	83.11	93.22	67.81	61.97	73.59
Jammu & Kashmir	Ramban	45	82.66	76.24	88.22	75.67	70.12	80.66
Jammu & Kashmir	Kishtwar	46	77.03	69.66	83.43	67.06	60.80	72.69
Jammu & Kashmir	Udhampur	47	89.50	83.88	93.88	86.55	82.56	90.20
Jammu & Kashmir	Reasi	48	87.87	82.30	92.61	87.75	83.73	91.28
Jammu & Kashmir	Jammu	49	85.81	79.38	91.03	87.63	83.32	91.15
Jammu & Kashmir	Samba	50	77.83	71.33	84.00	87.66	83.62	91.50
Himachal Pradesh	Chamba	51	95.79	92.44	98.15	89.28	85.12	92.82
Himachal Pradesh	Kangra	52	92.94	88.25	96.48	87.11	82.42	90.91
Himachal Pradesh	Lahul & Spiti	53	93.76	89.18	97.04	92.24	88.09	95.44
Himachal Pradesh	Kullu	54	96.46	93.36	98.60	94.12	90.96	96.45
Himachal Pradesh	Mandi	55	92.61	88.17	96.01	94.11	91.08	96.49
Himachal Pradesh	Hamirpur	56	92.17	87.19	96.00	93.84	90.65	96.34
Himachal Pradesh	Una	57	95.04	90.87	97.82	88.39	83.94	92.36
Himachal Pradesh	Bilaspur	58	94.80	90.89	97.43	91.73	87.93	94.85
Himachal Pradesh	Solan	59	95.87	92.24	98.25	89.67	85.82	93.07
Himachal Pradesh	Sirmaur	60	97.29	94.88	98.90	91.51	88.10	94.39
Himachal Pradesh	Shimla	61	95.75	92.51	98.11	91.93	88.03	95.03
Himachal Pradesh	Kinnaur	62	94.01	89.89	97.18	92.19	88.31	95.31
Punjab	Kapurthala	63	90.43	85.46	94.41	86.13	80.46	91.10
Punjab	Jalandhar	64	94.91	90.93	97.69	93.21	89.08	96.42
Punjab	Hoshiarpur	65	94.51	90.71	97.38	91.42	86.29	95.09
Punjab	Shahid Bhagat Singh Nagar	66	95.46	91.90	97.95	91.43	86.50	95.06
Punjab	Fatehgarh Sahib	67	94.93	91.15	97.62	88.37	82.83	92.94
Punjab	Ludhiana	68	88.83	82.94	93.63	89.35	83.95	93.81

Punjab	Moga	69	92.35	87.82	95.86	89.55	84.24	93.67
Punjab	Muktsar	70	95.17	91.69	97.67	93.43	89.40	96.41
Punjab	Faridkot	71	84.22	78.07	89.38	86.54	81.09	91.44
Punjab	Bathinda	72	79.10	72.16	85.03	88.93	83.94	93.18
Punjab	Mansa	73	91.18	86.53	95.13	89.27	83.93	93.58
Punjab	Patiala	74	84.67	78.75	89.72	87.78	82.16	92.25
Punjab	Amritsar	75	94.61	90.80	97.44	93.38	89.54	96.25
Punjab	Tarn Taran	76	86.04	80.42	90.94	89.33	84.60	93.49
Punjab	Rupnagar	77	89.90	84.04	94.48	91.59	86.65	95.49
Punjab	Sahibzada Ajit Singh Nagar	78	94.67	90.28	97.58	88.98	83.54	93.38
Punjab	Sangrur	79	92.27	87.92	95.72	92.05	87.57	95.50
Punjab	Barnala	80	94.41	90.71	97.24	91.39	86.49	95.13
Chandigarh	Chandigarh	81	94.51	89.37	97.95	95.27	91.07	98.12
Uttarakhand	Uttarkashi	82	94.85	91.22	97.49	89.20	84.10	93.31
Uttarakhand	Chamoli	83	93.51	89.01	96.79	90.36	85.21	94.60
Uttarakhand	Rudraprayag	84	93.14	88.68	96.49	80.36	73.23	86.62
Uttarakhand	Tehri Garhwal	85	90.88	85.89	94.74	91.99	87.55	95.38
Uttarakhand	Dehradun	86	88.71	82.21	93.73	89.54	85.50	92.81
Uttarakhand	Garhwal	87	91.14	85.72	95.16	91.77	87.09	95.29
Uttarakhand	Pithoragarh	88	96.13	92.97	98.31	95.05	91.24	97.63
Uttarakhand	Bageshwar	89	93.68	89.45	96.92	92.43	88.67	95.54
Uttarakhand	Almora	90	93.57	88.57	96.94	86.07	80.10	91.04
Uttarakhand	Champawat	91	94.05	90.12	96.95	91.51	87.00	95.02
Uttarakhand	Nainital	92	93.37	89.32	96.61	90.26	86.70	93.46
Uttarakhand	Udham Singh Nagar	93	91.46	87.12	95.22	91.59	88.13	94.50
Uttarakhand	Hardwar	94	87.23	81.13	92.18	89.34	85.44	92.70
Haryana	Panchkula	95	93.32	88.03	96.91	80.35	73.40	86.58
Haryana	Ambala	96	86.05	80.64	90.86	85.95	79.54	91.10
Haryana	Yamunanagar	97	87.57	82.42	92.08	88.90	83.53	93.09
Haryana	Kurukshetra	98	92.20	88.19	95.39	72.16	65.18	78.70
Haryana	Kaithal	99	87.66	81.25	92.98	76.12	69.40	82.45
Haryana	Karnal	100	91.54	87.50	94.94	79.34	72.93	85.06
Haryana	Panipat	101	90.21	85.25	94.36	89.40	84.03	93.75
Haryana	Sonipat	102	89.31	84.02	93.59	78.74	72.06	84.54
Haryana	Jind	103	88.66	83.38	93.02	82.32	75.80	87.97
Haryana	Fatehabad	104	91.75	86.77	95.60	81.11	74.95	86.48

Haryana	Sirsa	105	87.52	75.81	95.55	70.56	63.17	76.97
Haryana	Hisar	106	89.44	84.70	93.34	80.14	73.73	85.88
Haryana	Rohtak	107	87.29	81.76	92.04	70.82	63.49	77.62
Haryana	Jhajjar	108	85.68	79.56	90.98	84.43	78.45	89.81
Haryana	Mahendragarh	109	78.45	71.99	84.41	63.86	57.00	70.76
Haryana	Rewari	110	83.81	77.52	89.42	71.73	64.43	78.59
Haryana	Gurgaon	111	90.01	84.44	94.50	82.00	75.34	88.17
Haryana	Mewat	112	78.99	72.12	85.34	57.30	49.43	64.61
Haryana	Faridabad	113	87.79	82.88	92.22	72.80	65.25	79.66
Haryana	Palwal	114	81.69	75.99	86.70	78.00	71.67	84.07
Rajasthan	Ganganagar	115	92.32	88.05	95.52	76.47	70.13	82.27
Rajasthan	Hanumangarh	116	84.92	79.91	89.39	78.99	72.73	84.57
Rajasthan	Bikaner	117	86.31	81.52	90.53	87.52	83.59	90.94
Rajasthan	Churu	118	84.71	79.64	89.23	75.64	69.28	81.67
Rajasthan	Jhunjhunun	119	92.74	88.79	95.92	78.51	72.40	84.11
Rajasthan	Alwar	120	90.36	85.82	94.02	80.62	74.63	85.79
Rajasthan	Bharatpur	121	78.87	72.35	84.82	76.59	70.41	82.35
Rajasthan	Dhaulpur	122	87.86	82.82	92.34	87.79	82.78	91.79
Rajasthan	Karauli	123	82.62	77.47	87.37	87.24	82.07	91.58
Rajasthan	Sawai Madhopur	124	79.99	74.30	85.17	80.58	74.58	85.80
Rajasthan	Dausa	125	80.84	75.25	85.91	77.19	70.93	83.06
Rajasthan	Jaipur	126	89.86	85.59	93.36	86.62	82.72	90.13
Rajasthan	Sikar	127	88.56	83.68	92.58	82.78	77.04	88.00
Rajasthan	Nagaur	128	85.91	80.99	90.28	83.86	78.08	88.63
Rajasthan	Jodhpur	129	91.45	87.42	94.60	84.48	80.31	88.34
Rajasthan	Jaisalmer	130	88.78	83.98	92.66	76.28	69.95	82.32
Rajasthan	Barmer	131	91.28	87.24	94.69	71.81	64.00	79.01
Rajasthan	Jalor	132	89.18	84.52	93.01	77.51	70.80	83.69
Rajasthan	Sirohi	133	87.02	82.23	90.99	86.77	81.56	91.44
Rajasthan	Pali	134	84.32	78.67	89.22	85.01	78.95	90.15
Rajasthan	Ajmer	135	83.24	76.95	88.43	88.90	85.01	92.03
Rajasthan	Tonk	136	85.93	80.65	90.65	80.39	74.03	86.25
Rajasthan	Bundi	137	88.07	83.45	92.32	76.13	69.28	82.37
Rajasthan	Bhilwara	138	94.36	90.72	97.13	89.39	84.27	93.77
Rajasthan	Rajsamand	139	88.22	82.83	92.71	82.97	76.88	88.15
Rajasthan	Dungarpur	140	87.98	83.58	91.77	86.32	81.00	90.87

Rajasthan	Banswara	141	90.17	85.66	93.98	79.90	74.21	85.21
Rajasthan	Chittaurgarh	142	85.88	80.05	90.92	77.69	70.87	83.92
Rajasthan	Kota	143	85.96	80.28	91.04	83.32	78.96	87.38
Rajasthan	Baran	144	88.82	84.23	92.82	85.64	80.43	90.31
Rajasthan	Jhalawar	145	84.64	79.05	89.86	80.64	74.02	86.30
Rajasthan	Udaipur	146	89.73	85.07	93.75	81.04	74.55	86.85
Rajasthan	Pratapgarh	147	90.68	85.93	94.37	78.98	72.94	84.39
Uttar Pradesh	Saharanpur	148	94.34	90.73	97.18	84.34	79.93	88.27
Uttar Pradesh	Bijnor	149	87.62	82.50	92.09	79.46	73.25	85.14
Uttar Pradesh	Rampur	150	91.25	86.16	95.29	86.19	80.92	90.71
Uttar Pradesh	Jyotiba Phule Nagar	151	92.76	88.71	95.99	78.81	72.44	84.63
Uttar Pradesh	Meerut	152	90.93	86.49	94.69	82.66	78.91	86.11
Uttar Pradesh	Baghpat	153	90.86	86.32	94.54	82.18	76.18	87.51
Uttar Pradesh	Gautam Buddha Nagar	154	87.40	81.06	92.23	85.21	80.77	89.02
Uttar Pradesh	Bulandshahr	155	90.30	84.98	94.56	78.57	72.16	84.31
Uttar Pradesh	Aligarh	156	89.32	84.25	93.54	81.19	76.79	85.33
Uttar Pradesh	Mahamaya Nagar	157	81.56	75.31	87.00	77.18	70.86	83.05
Uttar Pradesh	Mathura	158	82.66	77.24	87.52	82.87	76.90	88.01
Uttar Pradesh	Agra	159	85.79	80.26	90.68	88.15	84.56	91.31
Uttar Pradesh	Firozabad	160	87.07	82.20	91.38	85.67	81.31	89.42
Uttar Pradesh	Mainpuri	161	90.53	85.92	94.37	81.83	75.92	87.15
Uttar Pradesh	Bareilly	162	87.17	81.18	92.32	87.50	83.65	90.92
Uttar Pradesh	Pilibhit	163	91.86	87.39	95.43	83.37	77.34	88.70
Uttar Pradesh	Shahjahanpur	164	87.45	82.54	91.75	85.63	79.96	90.30
Uttar Pradesh	Sitapur	165	84.70	78.91	89.96	84.45	78.47	89.64
Uttar Pradesh	Hardoi	166	84.85	79.26	89.69	67.02	59.72	74.34
Uttar Pradesh	Unnao	167	83.10	77.30	88.53	88.39	83.10	92.96
Uttar Pradesh	Lucknow	168	94.00	89.89	97.01	85.17	80.36	89.32
Uttar Pradesh	Farrukhabad	169	87.96	83.02	92.26	82.31	76.26	87.68
Uttar Pradesh	Kannauj	170	86.18	80.65	90.99	81.14	74.83	86.89
Uttar Pradesh	Etawah	171	85.50	80.05	90.22	88.32	83.07	92.81
Uttar Pradesh	Auraiya	172	88.05	83.07	92.39	71.86	65.41	78.11
Uttar Pradesh	Kanpur Dehat	173	80.81	74.41	86.50	88.71	83.50	93.06
Uttar Pradesh	Kanpur Nagar	174	84.13	78.16	89.05	86.37	81.96	90.14
Uttar Pradesh	Jalaun	175	85.13	79.78	89.71	74.32	67.02	81.29
Uttar Pradesh	Jhansi	176	91.74	87.70	95.00	86.37	82.05	90.19

Uttar Pradesh	Lalitpur	177	83.39	78.15	87.85	90.00	84.93	94.05
Uttar Pradesh	Hamirpur	178	90.29	85.70	94.12	78.10	70.64	84.55
Uttar Pradesh	Mahoba	179	84.92	79.49	89.66	84.81	78.13	90.40
Uttar Pradesh	Banda	180	81.90	75.11	87.70	87.98	82.37	92.41
Uttar Pradesh	Chitrakoot	181	83.89	77.59	89.18	86.11	80.58	90.78
Uttar Pradesh	Fatehpur	182	85.94	80.45	90.68	85.13	78.50	90.68
Uttar Pradesh	Pratapgarh	183	91.76	87.48	95.29	77.64	70.55	83.77
Uttar Pradesh	Kaushambi	184	90.71	85.21	94.91	81.61	75.11	87.28
Uttar Pradesh	Allahabad	185	85.47	79.31	90.89	85.57	79.93	90.55
Uttar Pradesh	Bara Banki	186	76.06	69.27	82.41	82.98	76.27	88.64
Uttar Pradesh	Faizabad	187	89.07	84.66	93.00	77.49	70.90	83.59
Uttar Pradesh	Ambedkar Nagar	188	91.97	87.58	95.46	80.12	74.51	85.16
Uttar Pradesh	Bahraich	189	74.95	67.99	81.36	76.86	70.22	83.03
Uttar Pradesh	Shrawasti	190	87.24	82.47	91.31	77.03	70.49	83.11
Chhattisgarh	Balrampur	191	90.19	84.94	94.12	92.24	81.81	97.95
Uttar Pradesh	Gonda	192	90.96	86.36	94.72	80.37	74.10	86.03
Uttar Pradesh	Siddharthnagar	193	89.10	84.80	92.95	71.54	64.80	78.12
Uttar Pradesh	Basti	194	93.53	89.74	96.34	85.01	79.00	90.07
Uttar Pradesh	Sant Kabir Nagar	195	91.60	87.45	95.01	82.61	76.65	87.67
Uttar Pradesh	Maharajganj	196	90.84	86.52	94.24	86.18	81.37	90.52
Uttar Pradesh	Gorakhpur	197	86.07	80.78	90.78	82.42	76.53	87.42
Uttar Pradesh	Kushinagar	198	90.69	86.08	94.52	78.56	72.49	84.08
Uttar Pradesh	Deoria	199	83.80	77.71	89.03	81.63	75.96	86.59
Uttar Pradesh	Azamgarh	200	88.40	83.04	93.03	80.97	74.81	86.41
Uttar Pradesh	Mau	201	84.97	79.16	89.80	78.35	72.16	84.28
Uttar Pradesh	Ballia	202	91.16	85.67	95.25	77.71	71.58	83.42
Uttar Pradesh	Jaunpur	203	85.38	80.50	89.87	74.23	68.12	80.16
Uttar Pradesh	Ghazipur	204	89.33	84.86	93.23	79.30	73.04	84.58
Uttar Pradesh	Chandauli	205	89.29	84.31	93.43	83.36	78.17	87.83
Uttar Pradesh	Varanasi	206	83.83	78.29	88.80	83.15	78.86	87.04
Uttar Pradesh	Sant Ravidas Nagar	207	90.81	86.56	94.57	73.81	67.52	79.58
Uttar Pradesh	Mirzapur	208	88.04	82.99	92.33	78.28	72.22	83.91
Uttar Pradesh	Sonbhadra	209	88.89	83.34	93.27	82.97	77.30	88.28
Uttar Pradesh	Etah	210	84.07	78.60	88.88	89.66	84.71	93.46
Uttar Pradesh	Kanshiram Nagar	211	84.98	79.34	89.66	75.04	67.93	81.22
Bihar	Pashchim Champaran	212	84.48	78.44	89.87	71.66	64.82	78.09

Bihar	Purba Champaran	213	90.07	85.39	93.89	72.02	65.48	78.26
Bihar	Sheohar	214	83.57	77.23	89.08	72.84	66.19	79.15
Bihar	Sitamarhi	215	88.85	83.99	93.00	81.73	75.98	87.08
Bihar	Madhubani	216	90.98	85.93	95.10	78.78	72.69	84.07
Bihar	Supaul	217	93.19	89.20	96.36	73.03	66.59	78.78
Bihar	Araria	218	90.36	86.01	94.01	84.26	78.80	89.20
Bihar	Kishanganj	219	88.68	83.29	93.33	72.13	65.39	78.28
Bihar	Purnia	220	87.08	82.16	91.48	72.39	65.64	78.44
Bihar	Katihar	221	85.62	79.36	90.98	63.87	56.20	70.97
Bihar	Madhepura	222	90.92	86.57	94.55	66.59	60.44	72.46
Bihar	Saharsa	223	85.30	79.70	90.24	73.69	67.19	79.80
Bihar	Darbhanga	224	87.03	81.64	91.73	84.96	79.72	89.62
Bihar	Muzaffarpur	225	89.96	84.96	94.04	65.75	58.46	73.04
Bihar	Gopalganj	226	90.41	85.48	94.49	82.59	76.64	87.76
Bihar	Siwan	227	87.52	82.12	92.15	62.00	55.25	68.27
Bihar	Saran	228	81.94	75.46	87.57	70.11	63.66	76.34
Bihar	Vaishali	229	89.18	84.17	93.43	62.94	56.24	69.51
Bihar	Samastipur	230	87.13	81.47	91.69	77.78	71.07	83.64
Bihar	Begusarai	231	80.99	74.80	86.62	82.43	76.94	87.22
Bihar	Khagaria	232	85.49	80.01	90.45	83.17	77.83	87.80
Bihar	Bhagalpur	233	85.45	80.02	90.43	73.99	68.05	79.88
Bihar	Banka	234	85.67	80.28	90.40	80.31	74.87	85.06
Bihar	Munger	235	82.00	75.95	87.14	69.21	61.92	76.36
Bihar	Lakhisarai	236	88.39	83.30	92.52	70.99	64.20	77.19
Bihar	Sheikhpura	237	82.09	76.10	87.57	85.01	79.71	89.65
Bihar	Nalanda	238	83.80	77.92	89.13	65.66	58.59	72.44
Bihar	Patna	239	83.33	76.98	88.84	84.44	80.38	88.13
Bihar	Bhojpur	240	82.04	76.38	87.18	77.01	71.35	82.32
Bihar	Buxer	241	79.08	72.12	85.50	79.17	73.38	84.58
Bihar	Kaimur (Bhabua)	242	81.51	75.22	87.32	74.46	68.12	80.52
Bihar	Rohtas	243	81.05	74.67	86.70	79.69	74.21	84.75
Bihar	Gaya	244	91.27	87.12	94.80	78.77	73.33	83.70
Bihar	Nawada	245	83.38	77.06	88.87	85.83	80.72	90.44
Bihar	Jamui	246	84.35	78.69	89.38	82.67	77.38	87.50
Bihar	Jehanabad	247	75.98	69.42	81.94	83.02	76.64	88.25
Bihar	Arwal	248	81.67	75.25	87.27	74.53	68.30	80.37

Sikkim	North District	249	92.62	87.59	96.42	89.79	85.03	93.56
Sikkim	West District	250	92.85	87.91	96.60	95.04	91.92	97.39
Sikkim	South District	251	92.80	87.62	96.57	94.07	87.70	98.08
Sikkim	East District	252	88.82	81.99	94.25	94.80	92.30	96.88
Arunachal Pradesh	Tawang	253	80.33	73.53	86.62	92.67	87.32	96.45
Arunachal Pradesh	West Kameng	254	80.38	73.84	86.04	83.64	77.23	89.17
Arunachal Pradesh	East Kameng	255	84.44	78.25	89.76	84.81	78.75	89.89
Arunachal Pradesh	Papum Pare	256	84.23	78.11	89.57	83.17	78.56	87.36
Arunachal Pradesh	Upper Subansiri	257	94.10	90.11	96.97	88.95	83.83	93.19
Arunachal Pradesh	Upper Siang	258	90.53	84.41	94.91	85.99	79.56	91.01
Arunachal Pradesh	Changlang	259	89.75	84.98	93.62	93.90	90.16	96.68
Arunachal Pradesh	Lower Subansiri	260	87.45	82.21	91.89	88.98	83.30	93.50
Arunachal Pradesh	Dibang Valley	261	90.58	84.95	94.94	88.56	82.02	93.44
Arunachal Pradesh	Lower Dibang Valley	262	89.56	84.63	93.74	87.05	81.34	91.58
Arunachal Pradesh	Anjaw	263	91.99	86.93	96.02	84.82	78.61	90.03
Nagaland	Mon	264	98.81	97.11	99.68	95.80	91.97	98.29
Nagaland	Mokokchung	265	99.00	97.48	99.76	97.66	94.88	99.20
Nagaland	Zunheboto	266	99.01	97.40	99.76	98.10	96.05	99.32
Nagaland	Wokha	267	99.04	97.49	99.76	97.10	93.81	98.92
Nagaland	Dimapur	268	98.81	97.11	99.69	97.25	95.09	98.69
Nagaland	Phek	269	99.04	97.57	99.76	97.83	95.59	99.20
Nagaland	Tuensang	270	99.05	97.49	99.75	97.66	95.06	99.16
Nagaland	Longleng	271	98.78	96.96	99.67	98.15	96.10	99.32
Nagaland	Kiphire	272	99.08	97.71	99.77	97.78	95.38	99.16
Nagaland	Kohima	273	98.34	96.15	99.57	96.55	93.74	98.53
Nagaland	Peren	274	99.10	97.73	99.77	97.42	94.96	99.03
Manipur	Senapati	275	94.91	90.34	97.89	93.63	90.01	96.37
Manipur	Tamenglong	276	93.11	88.18	96.59	96.86	94.34	98.55
Manipur	Churachandpur	277	90.18	84.44	94.71	96.98	94.20	98.72
Manipur	Bishnupur	278	93.81	89.87	96.87	96.78	94.71	98.33
Manipur	Thoubal	279	95.66	92.25	98.09	95.89	93.57	97.73
Manipur	Imphal West	280	94.69	90.36	97.73	95.84	93.22	97.75
Manipur	Imphal East	281	93.24	88.97	96.49	96.61	94.29	98.28
Manipur	Ukhrul	282	96.05	92.08	98.52	97.09	94.17	98.89
Manipur	Chandel	283	95.25	91.41	98.01	97.14	94.61	98.79
Mizoram	Mamit	284	98.45	96.49	99.54	97.70	95.48	99.06

Mizoram	Kolasib	285	98.12	95.74	99.41	97.54	95.50	98.88
Mizoram	Aizawl	286	98.31	95.95	99.52	95.96	92.25	98.32
Mizoram	Champhai	287	97.05	94.26	98.87	95.15	92.31	97.32
Mizoram	Serchhip	288	97.94	95.47	99.39	97.42	95.50	98.81
Mizoram	Lunglei	289	98.39	96.29	99.55	96.28	93.64	98.15
Mizoram	Lawngtlai	290	97.81	95.23	99.24	94.86	91.00	97.61
Tripura	Dhalai	291	82.33	76.10	87.76	92.59	88.66	95.61
Meghalaya	South Garo Hills	292	89.25	83.83	93.52	93.96	90.11	96.76
Meghalaya	Ribhoi	293	94.49	90.78	97.27	92.61	88.01	96.11
Meghalaya	East Khasi Hills	294	93.55	89.24	96.92	94.54	91.51	96.88
Assam	Kokrajhar	295	96.48	93.56	98.49	84.71	78.82	89.60
Assam	Goalpara	296	94.83	91.46	97.35	81.96	76.63	86.80
Assam	Barpeta	297	96.57	93.81	98.49	83.93	78.47	88.78
Assam	Morigaon	298	92.56	88.41	95.69	89.38	84.74	93.34
Assam	Lakhimpur	299	95.64	92.30	97.95	93.45	89.65	96.36
Assam	Dhemaji	300	95.01	91.53	97.49	93.02	89.09	96.04
Assam	Tinsukia	301	89.45	84.44	93.56	92.99	89.08	96.04
Assam	Dibrugarh	302	94.12	90.12	96.99	93.14	88.91	96.24
Assam	Golaghat	303	94.87	91.23	97.52	96.03	93.36	98.03
Assam	Dima Hasao	304	94.67	91.24	97.36	87.69	82.58	92.25
Assam	Cachar	305	79.81	73.91	85.32	84.96	79.34	89.87
Assam	Karimganj	306	84.71	78.56	89.96	85.13	79.61	89.76
Assam	Hailakandi	307	81.26	75.24	86.64	79.65	73.08	85.49
Assam	Bongaigaon	308	95.14	91.82	97.63	71.81	64.90	78.55
Assam	Chirang	309	89.34	84.70	93.31	77.25	71.46	82.59
Assam	Kamrup	310	96.09	92.75	98.32	79.99	73.86	85.50
Assam	Kamrup Metropolitan	311	94.67	90.99	97.25	92.97	88.23	96.41
Assam	Nalbari	312	91.89	87.83	95.23	72.11	65.83	78.04
Assam	Baksa	313	91.47	86.88	95.03	86.03	80.88	90.45
Assam	Darrang	314	95.82	92.84	97.98	82.50	76.59	87.71
Assam	Udalguri	315	91.19	86.55	94.94	75.68	69.44	81.26
West Bengal	Darjiling	316	95.60	92.17	97.98	86.30	80.11	91.32
West Bengal	Jalpaiguri	317	93.28	89.30	96.37	89.69	84.27	93.74
West Bengal	Koch Bihar	318	91.46	86.96	95.15	93.10	88.96	96.24
West Bengal	Uttar Dinajpur	319	90.09	85.37	93.97	88.85	83.77	93.12
West Bengal	Dakshin Dinajpur	320	92.50	88.49	95.75	90.59	85.65	94.41

West Bengal	Maldah	321	88.15	83.28	92.37	88.86	83.80	93.25
West Bengal	Murshidabad	322	91.47	87.26	94.93	90.23	85.95	93.87
West Bengal	Birbhum	323	88.47	83.81	92.70	88.52	83.46	92.83
West Bengal	Nadia	324	88.43	83.28	92.76	89.90	85.08	93.73
West Bengal	North Twenty Four Parganas	325	94.46	90.75	97.14	87.88	81.78	92.66
West Bengal	Hugli	326	87.97	82.85	92.22	91.71	86.80	95.46
West Bengal	Bankura	327	88.02	82.58	92.62	89.01	84.06	93.09
West Bengal	Puruliya	328	89.63	84.53	93.79	87.66	82.26	92.00
West Bengal	South Twenty Four Parganas	329	80.47	74.58	85.69	93.02	88.75	96.28
West Bengal	Paschim Medinipur	330	90.79	85.77	94.52	87.44	82.25	91.96
West Bengal	Purba Medinipur	331	83.23	77.08	88.57	89.83	85.45	93.49
Jharkhand	Garhwa	332	96.07	93.07	98.21	92.30	87.78	95.67
Jharkhand	Chatra	333	89.20	84.64	93.23	83.55	77.48	88.77
Jharkhand	Kodarma	334	88.85	84.15	92.78	85.73	80.55	90.02
Jharkhand	Giridih	335	91.19	86.69	94.70	80.77	75.18	85.65
Jharkhand	Deoghar	336	91.33	86.92	94.83	86.60	81.38	90.92
Jharkhand	Godda	337	87.19	81.13	92.30	90.00	84.96	94.06
Jharkhand	Sahibganj	338	91.70	87.30	95.25	86.95	81.92	91.43
Jharkhand	Pakur	339	93.98	89.84	97.01	86.17	81.09	90.75
Jharkhand	Dhanbad	340	92.94	88.71	96.24	77.47	72.46	81.83
Jharkhand	Bokaro	341	92.87	88.51	96.15	83.06	78.88	87.20
Jharkhand	Lohardaga	342	96.57	93.43	98.60	89.87	84.63	94.14
Jharkhand	Purbi Singhbhum	343	94.84	91.34	97.48	90.50	86.99	93.55
Jharkhand	Palamu	344	89.89	85.28	93.61	85.74	79.60	90.77
Jharkhand	Latehar	345	86.01	80.93	90.56	89.05	83.92	92.98
Jharkhand	Hazaribagh	346	90.04	85.60	93.79	83.46	78.09	88.19
Jharkhand	Ramgarh	347	91.41	86.81	94.93	93.19	90.06	95.65
Jharkhand	Dumka	348	93.32	88.84	96.59	92.21	87.53	95.85
Jharkhand	Jamtara	349	88.02	83.21	92.40	89.68	85.23	93.47
Jharkhand	Ranchi	350	92.59	88.35	96.09	91.46	87.74	94.52
Jharkhand	Khunti	351	84.40	78.30	89.60	87.05	81.46	91.84
Jharkhand	Gumla	352	82.69	76.99	87.89	89.70	84.67	93.91
Jharkhand	Simdega	353	94.40	90.53	97.32	94.65	91.15	97.18
Jharkhand	Pashchimi Singhbhum	354	85.16	79.20	90.25	91.20	86.45	94.98
Jharkhand	Saraikela-Kharsawan	355	78.52	72.77	84.28	93.08	88.72	96.41
Odisha	Bargarh	356	91.70	86.94	95.48	82.35	76.04	87.90

Odisha	Jharsuguda	357	94.67	90.54	97.56	84.17	79.87	88.14
Odisha	Sambalpur	358	94.93	91.17	97.63	86.61	80.68	91.40
Odisha	Debagarh	359	90.58	84.97	94.86	82.56	76.32	87.85
Odisha	Sundargarh	360	95.34	91.47	97.91	86.57	82.37	90.26
Odisha	Kendujhar	361	88.57	83.18	92.87	78.82	72.36	84.93
Odisha	Mayurbhanj	362	91.78	87.09	95.50	81.43	74.60	86.81
Odisha	Baleshwar	363	87.69	81.87	92.51	79.34	72.93	85.20
Odisha	Bhadrak	364	87.52	81.80	92.32	81.19	74.11	87.17
Odisha	Kendrapara	365	86.97	81.16	91.87	76.37	68.43	83.37
Odisha	Cuttack	366	86.21	79.68	91.76	80.74	73.76	86.86
Odisha	Jajapur	367	82.89	76.65	88.70	81.36	75.36	86.44
Odisha	Dhenkanal	368	90.78	85.51	94.79	78.81	72.46	84.51
Odisha	Anugul	369	92.43	88.02	95.71	86.36	81.42	90.92
Odisha	Nayagarh	370	89.94	85.10	93.80	82.71	76.83	87.96
Odisha	Khordha	371	91.61	86.90	95.31	82.95	78.32	87.12
Odisha	Puri	372	90.87	85.61	94.90	85.79	80.65	90.35
Odisha	Ganjam	373	90.54	85.47	94.48	78.12	71.03	84.56
Odisha	Gajapati	374	96.44	93.16	98.47	86.93	81.25	91.70
Odisha	Kandhamal	375	91.50	86.40	95.36	89.47	84.64	93.42
Odisha	Baudh	376	93.61	89.44	96.64	82.67	76.42	88.16
Odisha	Subarnapur	377	93.71	89.42	96.89	82.95	76.92	88.45
Odisha	Balangir	378	94.35	90.34	97.16	84.73	78.90	89.58
Odisha	Nuapada	379	95.53	92.04	97.98	83.25	77.30	88.52
Odisha	Kalahandi	380	89.38	84.30	93.61	78.39	71.13	84.72
Odisha	Rayagada	381	88.57	82.98	93.05	83.52	77.15	88.94
Odisha	Nabarangapur	382	93.85	90.10	96.96	85.64	80.21	90.17
Odisha	Koraput	383	94.76	91.23	97.38	83.31	77.16	88.46
Odisha	Malkangiri	384	90.78	86.06	94.67	80.35	74.31	85.80
Chhattisgarh	Koriya	385	94.38	90.30	97.22	90.62	87.27	93.60
Chhattisgarh	Jashpur	386	95.98	92.60	98.24	90.26	85.63	94.11
Chhattisgarh	Raigarh	387	92.52	88.35	96.07	90.32	85.70	94.12
Chhattisgarh	Korba	388	94.65	90.96	97.33	91.39	87.91	94.30
Chhattisgarh	Janjgir-Champa	389	92.46	87.86	95.94	91.22	86.92	94.75
Chhattisgarh	Kabeerdham	390	92.73	87.67	96.48	87.23	81.91	91.83
Chhattisgarh	Rajnandgaon	391	91.98	87.86	95.51	92.83	89.19	95.73
Chhattisgarh	Mahasamund	392	90.12	85.32	93.97	89.70	84.92	93.63

Chhattisgarh	Dhamtari	393	91.67	87.10	95.51	91.00	86.79	94.56
Chhattisgarh	Uttar Bastar Kanker	394	88.09	81.88	92.84	89.46	84.24	93.74
Chhattisgarh	Narayanpur	395	92.73	88.60	96.09	86.57	81.53	90.89
Chhattisgarh	Bijapur	396	93.47	89.16	96.59	92.06	87.96	95.17
Madhya Pradesh	Sheopur	397	85.05	79.12	90.17	85.63	80.10	90.43
Madhya Pradesh	Morena	398	89.83	85.15	93.75	82.98	77.67	87.83
Madhya Pradesh	Bhind	399	82.91	76.41	88.46	85.09	79.18	89.87
Madhya Pradesh	Gwalior	400	86.87	80.68	92.01	83.61	79.33	87.51
Madhya Pradesh	Datia	401	88.91	83.48	93.59	88.38	83.07	92.75
Madhya Pradesh	Shivpuri	402	85.56	78.95	91.51	82.55	77.03	87.11
Madhya Pradesh	Tikamgarh	403	89.75	83.52	94.64	83.87	77.95	88.91
Madhya Pradesh	Chhatarpur	404	86.71	81.04	91.35	81.16	74.63	87.05
Madhya Pradesh	Panna	405	81.49	75.32	86.84	77.76	71.49	83.52
Madhya Pradesh	Sagar	406	82.69	76.57	87.79	84.73	78.34	90.21
Madhya Pradesh	Damoh	407	83.30	76.89	88.73	76.55	69.99	82.59
Madhya Pradesh	Satna	408	90.47	84.14	95.19	77.85	71.96	83.28
Madhya Pradesh	Rewa	409	88.02	81.16	93.39	85.59	80.37	90.11
Madhya Pradesh	Umaria	410	85.35	79.55	90.24	79.09	72.95	84.50
Madhya Pradesh	Neemuch	411	86.32	80.88	91.05	83.48	77.67	88.33
Madhya Pradesh	Mandsaur	412	93.47	89.35	96.62	80.14	74.58	85.26
Madhya Pradesh	Ratlam	413	79.83	73.21	85.47	84.87	79.53	89.69
Madhya Pradesh	Ujjain	414	81.66	75.71	87.28	77.65	73.09	81.90
Madhya Pradesh	Dewas	415	84.79	79.46	89.55	77.68	71.47	83.05
Madhya Pradesh	Dhar	416	84.48	77.21	90.84	82.93	77.99	87.55
Madhya Pradesh	Indore	417	84.05	78.46	88.89	88.57	85.06	91.76
Madhya Pradesh	Khargone (West Nimar)	418	82.82	76.81	88.20	84.43	79.43	88.88
Madhya Pradesh	Barwani	419	76.49	70.39	82.18	85.59	80.57	89.96
Madhya Pradesh	Rajgarh	420	69.98	62.33	77.14	77.85	71.08	83.94
Madhya Pradesh	Vidisha	421	83.06	77.13	88.27	69.86	62.50	76.52
Madhya Pradesh	Bhopal	422	84.63	73.36	92.96	81.41	75.37	86.72
Madhya Pradesh	Sehore	423	73.52	66.12	80.30	82.71	76.91	87.66
Madhya Pradesh	Raisen	424	89.87	80.76	96.28	82.81	76.84	88.15
Madhya Pradesh	Betul	425	92.88	88.53	96.12	82.11	75.84	87.71
Madhya Pradesh	Harda	426	90.16	85.54	94.03	86.36	81.36	90.77
Madhya Pradesh	Hoshangabad	427	85.61	79.71	90.72	91.07	87.70	93.92
Madhya Pradesh	Katni	428	90.49	83.96	95.19	84.43	78.62	89.36

Madhya Pradesh	Jabalpur	429	89.16	78.28	96.37	90.78	87.37	93.68
Madhya Pradesh	Narsimhapur	430	92.00	87.55	95.43	88.08	82.94	92.36
Madhya Pradesh	Dindori	431	90.26	85.31	94.38	88.43	83.07	92.76
Madhya Pradesh	Mandla	432	91.74	87.52	95.17	88.25	83.60	92.36
Madhya Pradesh	Chhindwara	433	86.84	80.49	91.87	88.56	83.59	92.76
Madhya Pradesh	Seoni	434	93.77	89.70	96.85	82.68	76.39	87.78
Madhya Pradesh	Balaghat	435	89.97	84.25	94.36	88.79	83.72	93.04
Madhya Pradesh	Guna	436	79.55	72.99	85.33	84.89	79.56	89.35
Madhya Pradesh	Ashoknagar	437	89.45	83.50	94.15	83.23	77.58	88.44
Madhya Pradesh	Shahdol	438	93.81	89.46	97.16	82.48	76.48	87.76
Madhya Pradesh	Anuppur	439	94.69	90.98	97.46	79.97	73.62	85.93
Madhya Pradesh	Singrauli	440	91.00	84.67	95.70	82.80	77.09	87.69
Madhya Pradesh	Jhabua	441	85.32	79.32	90.19	84.37	78.90	89.01
Madhya Pradesh	Alirajpur	442	85.33	79.89	90.09	83.69	78.56	88.26
Madhya Pradesh	Khandwa (East Nimar)	443	92.48	79.44	98.56	88.02	83.08	92.22
Madhya Pradesh	Burhanpur	444	84.34	78.55	89.42	85.39	81.63	89.09
Gujarat	Kachchh	445	91.53	86.97	95.13	82.49	77.23	87.15
Gujarat	Banas Kantha	446	86.58	80.83	91.60	88.21	84.02	91.91
Gujarat	Patan	447	94.01	90.03	96.81	75.52	70.24	80.61
Gujarat	Mahesana	448	92.81	87.99	96.38	91.31	87.48	94.46
Gujarat	Gandhinagar	449	87.56	81.38	92.45	87.65	83.23	91.35
Gujarat	Porbandar	450	89.44	84.48	93.68	77.09	71.21	82.66
Gujarat	Anand	451	90.81	85.89	94.89	92.20	88.49	95.03
Gujarat	Dohad	452	84.13	78.51	89.01	81.52	76.16	86.25
Gujarat	Narmada	453	94.88	91.14	97.55	91.52	87.75	94.55
Gujarat	Bharuch	454	89.21	83.42	93.89	90.99	87.00	94.33
Gujarat	The Dangs	455	92.65	88.07	96.19	92.25	88.68	95.22
Gujarat	Navsari	456	92.72	88.61	96.03	92.70	88.95	95.57
Gujarat	Valsad	457	91.69	87.11	95.34	92.67	89.09	95.64
Gujarat	Tapi	458	94.53	90.93	97.23	88.46	84.00	92.35
Dadra & Nagar Haveli And Daman & Diu	Daman	459	97.81	95.34	99.34	87.21	81.06	91.99
Dadra & Nagar Haveli And Daman & Diu	Dadra & Nagar Haveli	460	92.90	88.20	96.47	77.07	70.00	83.74
Maharashtra	Nandurbar	461	89.22	84.11	93.60	77.09	69.68	83.57
Maharashtra	Dhule	462	82.15	75.50	87.86	88.81	83.33	93.15
Maharashtra	Jalgaon	463	87.91	82.23	92.54	89.17	83.46	93.35
Maharashtra	Buldana	464	83.53	77.22	88.90	91.77	87.19	95.28

Maharashtra	Akola	465	88.91	84.21	92.86	90.50	85.60	94.42
Maharashtra	Washim	466	87.26	82.12	91.58	90.60	85.84	94.43
Maharashtra	Amravati	467	88.88	83.59	93.12	90.78	85.86	94.78
Maharashtra	Wardha	468	91.02	86.32	94.84	89.13	83.90	93.38
Maharashtra	Nagpur	469	92.02	87.53	95.56	93.24	89.06	96.25
Maharashtra	Bhandara	470	87.16	81.29	92.03	92.82	88.32	96.18
Maharashtra	Gondiya	471	93.18	89.10	96.37	88.80	83.22	93.31
Maharashtra	Gadchiroli	472	80.67	73.81	86.52	90.73	85.60	94.78
Maharashtra	Chandrapur	473	86.63	80.89	91.64	89.07	83.90	93.33
Maharashtra	Yavatmal	474	85.70	79.97	90.78	93.79	89.96	96.82
Maharashtra	Nanded	475	82.90	76.79	88.36	84.28	79.15	88.72
Maharashtra	Hingoli	476	85.05	79.84	89.71	84.27	78.68	89.26
Maharashtra	Parbhani	477	82.61	76.58	87.95	89.25	83.90	93.58
Maharashtra	Jalna	478	80.11	73.18	86.25	74.60	67.52	80.98
Maharashtra	Nashik	479	88.90	82.96	93.75	90.84	86.29	94.64
Maharashtra	Mumbai Suburban	480	91.89	85.31	96.44	90.00	83.91	94.61
Maharashtra	Mumbai	481	94.07	89.58	97.21	89.47	81.69	95.04
Maharashtra	Raigarh	482	95.20	91.62	97.72	95.06	91.32	97.70
Maharashtra	Pune	483	92.51	87.69	96.06	93.38	89.32	96.59
Maharashtra	Ahmadnagar	484	88.80	82.99	93.36	88.07	82.31	92.82
Maharashtra	Bid	485	79.35	72.12	85.96	90.20	84.85	94.37
Maharashtra	Latur	486	85.52	79.22	91.00	88.19	83.20	92.42
Maharashtra	Osmanabad	487	90.81	86.14	94.72	88.21	82.47	92.82
Maharashtra	Solapur	488	89.61	84.00	94.41	88.81	84.05	92.95
Maharashtra	Satara	489	94.03	90.20	97.01	91.66	86.98	95.41
Maharashtra	Ratnagiri	490	93.93	89.36	97.11	92.15	87.05	96.01
Maharashtra	Sindhudurg	491	93.20	88.16	96.85	91.17	85.27	95.60
Maharashtra	Kolhapur	492	95.54	92.16	97.90	87.41	81.77	92.04
Maharashtra	Sangli	493	86.14	81.33	90.28	85.66	79.70	90.91
Karnataka	Belgaum	494	83.46	77.35	88.78	90.51	85.69	94.53
Karnataka	Bagalkot	495	81.18	74.87	86.97	62.07	54.15	69.55
Karnataka	Bijapur	496	84.54	78.10	89.99	76.88	70.25	82.82
Karnataka	Bidar	497	80.92	74.52	86.42	77.52	71.03	83.30
Karnataka	Raichur	498	76.33	69.30	82.52	74.26	66.82	80.76
Karnataka	Koppal	499	84.65	78.48	89.89	84.02	77.51	89.39
Karnataka	Gadag	500	69.52	62.54	75.80	68.32	60.67	75.27

Karnataka	Dharwad	501	86.20	80.18	91.31	81.84	74.98	87.88
Karnataka	Uttara Kannada	502	82.95	76.33	88.35	76.06	68.72	82.44
Karnataka	Haveri	503	80.08	73.63	85.74	82.83	76.00	88.59
Karnataka	Bellary	504	76.43	69.69	82.66	60.62	53.05	68.02
Karnataka	Chitradurga	505	85.97	79.80	91.11	80.78	74.08	86.72
Karnataka	Davanagere	506	75.17	67.48	82.20	75.92	68.47	82.89
Karnataka	Shimoga	507	81.70	75.50	87.01	79.90	71.99	86.68
Karnataka	Chikmagalur	508	80.38	73.61	86.56	84.82	78.58	90.24
Karnataka	Tumkur	509	85.26	78.54	91.12	85.85	79.85	90.93
Karnataka	Bangalore	510	89.31	83.71	93.97	83.93	77.84	89.63
Karnataka	Mandya	511	87.13	81.15	91.93	78.61	71.01	85.50
Karnataka	Hassan	512	86.17	80.51	90.92	86.75	80.77	91.44
Karnataka	Dakshina Kannada	513	80.53	73.40	86.78	83.59	76.87	89.28
Karnataka	Kodagu	514	89.88	84.50	94.05	76.06	68.70	82.84
Karnataka	Mysore	515	76.01	68.29	82.74	81.53	74.72	87.34
Karnataka	Chamarajanagar	516	76.16	69.36	82.34	77.49	70.46	84.11
Karnataka	Gulbarga	517	77.68	70.82	84.16	67.48	59.51	75.22
Karnataka	Yadgir	518	73.07	66.09	79.48	85.96	79.78	90.94
Karnataka	Kolar	519	81.86	75.26	87.55	82.15	75.38	87.82
Karnataka	Chikkaballapura	520	83.75	77.56	89.27	74.37	67.03	81.09
Karnataka	Bangalore Rural	521	87.32	81.19	92.30	82.05	75.66	87.75
Karnataka	Ramanagara	522	78.77	70.95	85.56	85.07	78.93	90.23
Goa	North Goa	523	88.83	82.44	93.94	96.42	94.41	98.01
Goa	South Goa	524	96.29	92.75	98.55	86.71	82.85	90.25
Kerala	Wayanad	525	90.72	85.24	95.00	90.83	86.81	94.26
Kerala	Kozhikode	526	91.34	86.52	95.03	94.34	90.93	96.94
Kerala	Malappuram	527	89.71	84.69	93.64	86.43	81.70	90.41
Kerala	Palakkad	528	95.69	92.12	98.13	88.27	83.85	92.07
Kerala	Thrissur	529	93.39	88.39	96.91	92.60	88.33	95.90
Kerala	Ernakulam	530	96.25	92.56	98.51	92.97	88.85	96.18
Kerala	Idukki	531	97.28	94.48	99.03	91.17	86.51	94.94
Kerala	Kottayam	532	94.79	90.69	97.62	93.65	89.69	96.64
Kerala	Pathanamthitta	533	95.85	92.04	98.34	94.59	90.68	97.39
Kerala	Kollam	534	97.17	94.16	98.96	93.16	89.36	96.09
Kerala	Thiruvananthapuram	535	94.79	90.38	97.69	92.90	88.70	96.12
Tamil Nadu	Chennai	536	92.20	86.87	96.14	85.67	80.00	90.44

Tamil Nadu	Kancheepuram	537	83.69	76.61	89.72	88.10	83.12	92.34
Tamil Nadu	Vellore	538	95.81	92.25	98.11	79.85	74.05	85.04
Tamil Nadu	Tiruvannamalai	539	92.17	86.87	96.17	79.81	74.00	85.12
Tamil Nadu	Viluppuram	540	92.48	87.84	96.32	86.67	81.60	90.84
Tamil Nadu	Salem	541	92.33	87.16	96.04	81.50	75.97	86.51
Tamil Nadu	Namakkal	542	92.02	87.35	95.80	86.61	81.36	91.04
Tamil Nadu	Erode	543	95.43	91.84	97.82	89.89	85.28	93.75
Tamil Nadu	The Nilgiris	544	96.33	93.01	98.42	77.68	71.97	83.23
Tamil Nadu	Dindigul	545	92.75	88.72	95.89	84.84	79.63	89.44
Tamil Nadu	Karur	546	92.59	87.36	96.45	84.23	78.70	88.99
Tamil Nadu	Tiruchirappalli	547	92.28	86.75	96.17	83.75	78.09	88.83
Tamil Nadu	Perambalur	548	94.35	90.00	97.27	85.30	80.13	89.57
Tamil Nadu	Ariyalur	549	90.65	85.64	94.55	80.98	75.39	86.17
Tamil Nadu	Cuddalore	550	93.78	88.68	97.31	83.09	77.20	88.16
Tamil Nadu	Nagapattinam	551	92.51	87.44	96.13	85.43	79.83	90.08
Tamil Nadu	Thiruvarur	552	93.75	89.11	97.06	89.68	85.01	93.63
Tamil Nadu	Thanjavur	553	95.32	91.18	97.96	83.80	77.82	88.78
Tamil Nadu	Pudukkottai	554	85.08	78.29	90.75	82.39	76.78	87.69
Tamil Nadu	Sivaganga	555	92.79	88.08	96.23	85.04	79.47	89.89
Tamil Nadu	Madurai	556	94.25	90.02	97.33	84.79	79.30	89.60
Tamil Nadu	Theni	557	91.25	86.73	95.05	85.73	80.15	90.38
Tamil Nadu	Virudhunagar	558	90.39	84.18	94.87	77.54	70.53	84.01
Tamil Nadu	Thoothukkudi	559	92.43	87.36	96.18	76.32	69.15	82.69
Tamil Nadu	Tirunelveli	560	93.46	88.16	96.93	87.13	81.20	91.87
Tamil Nadu	Kanniyakumari	561	94.68	90.55	97.52	86.94	82.12	91.21
Tamil Nadu	Dharmapuri	562	94.55	90.46	97.45	75.20	69.63	80.47
Tamil Nadu	Krishnagiri	563	94.83	91.26	97.43	83.44	78.39	87.81
Tamil Nadu	Coimbatore	564	95.53	91.91	98.05	81.27	75.58	86.54
Tamil Nadu	Tiruppur	565	93.56	88.60	96.98	85.81	81.09	89.83
Puducherry	Yanam	566	92.94	88.42	96.32	80.17	74.62	85.29
Puducherry	Puducherry	567	96.61	93.21	98.74	84.13	79.00	88.95
Arunachal Pradesh	East Siang	568	87.95	82.59	92.30	85.63	78.99	90.86
Arunachal Pradesh	Kra Daadi	569	80.41	72.07	87.69	88.75	78.56	95.66
Arunachal Pradesh	Kurung Kumey	570	82.95	74.60	90.09	88.70	81.86	93.88
Arunachal Pradesh	Lohit	571	89.30	83.97	93.70	92.47	83.85	97.54
Arunachal Pradesh	Longding	572	91.61	86.74	95.42	94.97	90.04	98.16

Arunachal Pradesh	Namsai	573	81.19	75.79	86.20	84.34	77.65	90.33
Arunachal Pradesh	Siang	574	90.69	85.82	94.61	92.56	85.41	96.95
Arunachal Pradesh	Tirap	575	82.49	75.78	88.45	92.36	87.05	96.28
Arunachal Pradesh	West Siang	576	88.64	83.03	93.29	85.35	78.48	91.08
Assam	Biswanath	577	92.85	88.77	96.29	83.71	73.76	91.54
Assam	Charaideo	578	91.47	87.17	94.94	94.43	87.23	98.26
Assam	Dhubri	579	93.90	89.89	96.82	89.06	83.12	93.87
Assam	Hojai	580	95.09	91.47	97.74	92.32	85.63	96.75
Assam	Jorhat	581	92.36	87.84	95.87	95.33	91.99	97.72
Assam	Karbi Anglong	582	90.81	85.68	94.77	92.99	89.01	96.12
Assam	Majuli	583	94.15	90.31	96.94	95.69	88.54	99.05
Assam	Nagaon	584	90.93	86.26	94.54	91.21	86.21	95.13
Assam	Sivasagar	585	94.42	90.60	97.19	91.72	86.72	95.49
Assam	Sonitpur	586	94.52	90.80	97.25	91.31	86.35	95.36
Assam	South Salmara Mancachar	587	94.80	91.47	97.37	86.57	79.21	92.44
Assam	West Karbi Anglong	588	91.60	87.24	95.35	95.09	85.26	99.30
Chhattisgarh	Balod	589	94.07	90.16	97.00	77.35	64.14	87.89
Chhattisgarh	Baloda Bazar	590	91.54	86.82	95.17	92.00	84.65	96.81
Uttar Pradesh	Balrampur	591	86.99	81.38	91.74	65.78	59.22	71.94
Chhattisgarh	Bastar	592	91.42	86.58	95.19	89.85	84.12	94.25
Chhattisgarh	Bemetara	593	93.44	88.99	96.78	90.52	83.34	95.31
Chhattisgarh	Bilaspur	594	89.83	85.24	93.82	89.19	84.05	93.52
Chhattisgarh	Dantewada	595	93.01	88.98	96.22	89.00	81.32	94.67
Chhattisgarh	Durg	596	91.57	87.16	95.11	92.10	88.54	95.02
Chhattisgarh	Gariyaband	597	95.25	91.77	97.76	83.70	70.00	93.58
Chhattisgarh	Kodagaon	598	95.06	91.57	97.65	91.68	86.00	95.81
Chhattisgarh	Mungeli	599	92.42	87.39	96.17	90.50	81.10	96.45
Chhattisgarh	Raipur	600	95.33	91.56	97.79	91.82	88.29	94.74
Chhattisgarh	Sukma	601	93.73	90.23	96.58	86.34	79.78	91.85
Chhattisgarh	Surguja	602	94.24	90.23	97.24	94.15	88.00	97.93
Nct Of Delhi	Central	603	87.14	80.69	92.49	67.93	60.35	74.99
Nct Of Delhi	East	604	92.65	88.17	96.11	75.42	64.20	85.61
Nct Of Delhi	New Delhi	605	88.73	83.19	93.14	68.37	59.17	76.46
Nct Of Delhi	North	606	88.60	82.92	93.28	74.98	60.84	86.19
Nct Of Delhi	North East	607	92.18	87.50	95.86	75.76	67.67	83.15
Nct Of Delhi	North West	608	92.48	87.70	96.02	78.34	66.84	87.63

Nct Of Delhi	Shahdara	609	92.98	88.42	96.35	72.98	59.79	84.89
Nct Of Delhi	South	610	95.77	92.32	98.19	81.59	70.64	90.27
Nct Of Delhi	South East	611	94.01	89.98	96.94	65.72	52.46	77.24
Nct Of Delhi	South West	612	93.48	89.08	96.65	77.94	68.85	85.85
Nct Of Delhi	West	613	93.55	89.38	96.66	78.19	70.65	85.01
Gujarat	Ahmadabad	614	95.67	92.21	98.10	90.22	85.93	93.68
Gujarat	Aravali	615	94.19	90.72	96.96	90.87	85.10	95.15
Gujarat	Bhavnagar	616	94.18	89.72	97.32	76.65	70.05	82.37
Gujarat	Botad	617	95.32	91.71	97.84	75.67	65.58	84.75
Gujarat	Chhota Udaipur	618	89.40	84.83	93.59	83.17	73.02	91.07
Gujarat	Devbhumi Dwarka	619	85.02	79.58	89.82	89.90	82.83	95.21
Gujarat	Gir Somnath	620	97.10	94.52	98.75	86.08	78.79	92.06
Gujarat	Jamnagar	621	90.29	84.81	94.35	81.62	76.06	86.88
Gujarat	Junagadh	622	87.54	82.08	91.96	81.54	75.11	87.43
Gujarat	Kheda	623	87.04	81.88	91.76	81.88	76.95	86.14
Gujarat	Mahisagar	624	86.82	81.31	91.53	78.53	69.14	86.58
Gujarat	Morbi	625	89.59	84.70	93.67	82.44	73.40	89.61
Gujarat	Panch Mahals	626	94.88	91.52	97.39	80.78	75.04	85.99
Gujarat	Rajkot	627	96.84	94.04	98.73	85.94	80.71	90.37
Gujarat	Sabar Kantha	628	95.36	91.67	97.93	90.77	86.49	94.57
Gujarat	Surendranagar	629	84.38	78.03	89.76	85.08	80.22	89.67
Gujarat	Vadodara	630	85.85	80.38	90.75	91.42	87.03	94.88
Haryana	Bhiwani	631	86.95	81.18	91.58	71.06	63.10	78.40
Haryana	Charkhi Dadri	632	89.60	83.86	94.36	83.09	73.33	90.77
Madhya Pradesh	Agar Malwa	633	90.54	83.78	95.57	77.60	66.83	86.70
Madhya Pradesh	Shajapur	634	80.73	74.58	86.20	79.84	72.93	86.05
Maharashtra	Palghar	635	93.99	89.44	97.19	92.79	78.73	98.98
Maharashtra	Thane	636	94.04	88.54	97.52	85.83	78.88	91.67
Meghalaya	East Garo Hills	637	89.02	84.27	93.08	90.61	82.83	96.07
Meghalaya	East Jantia Hills	638	97.01	94.42	98.72	94.91	89.17	98.19
Meghalaya	North Garo Hills	639	72.52	65.67	78.86	89.92	85.09	93.78
Meghalaya	South West Garo Hills	640	88.95	83.33	93.33	85.70	74.55	93.70
Meghalaya	South West Khasi Hills	641	96.21	92.98	98.34	95.07	87.44	99.03
Meghalaya	West Garo Hills	642	86.81	82.04	91.28	91.02	86.73	94.63
Meghalaya	West Jaintia Hills	643	94.11	90.32	97.01	93.40	88.72	96.79
Meghalaya	West Khasi Hills	644	96.93	94.40	98.73	96.05	92.49	98.32

Punjab	Fazilka	645	91.28	86.92	94.91	90.86	84.48	95.61
Punjab	Firozpur	646	93.42	89.47	96.53	93.41	87.83	97.03
Punjab	Gurdaspur	647	95.41	91.95	97.91	91.22	85.75	95.34
Punjab	Pathankot	648	90.31	84.75	94.72	79.79	63.44	91.74
Telangana	Adilabad	649	84.76	78.62	89.89	89.48	76.15	96.83
Telangana	Bhadradri Kothagudem	650	84.22	77.42	90.14	72.95	62.90	82.19
Telangana	Hyderabad	651	91.42	85.74	95.54	79.74	72.44	86.21
Telangana	Jagitial	652	88.27	83.04	92.45	87.39	76.45	94.95
Telangana	Jangoan	653	87.17	81.02	92.26	NA	NA	NA
Telangana	Jayashankar Bhupalapally	654	95.11	91.33	97.73	85.36	69.34	95.16
Telangana	Jogulamba Gadwal	655	82.19	75.37	88.03	75.71	51.12	92.86
Telangana	Kamareddy	656	75.77	68.16	82.68	83.02	73.65	90.44
Telangana	Karimnagar	657	85.23	78.73	90.69	86.47	75.48	94.30
Telangana	Khammam	658	89.35	83.73	93.85	67.92	53.70	80.31
Telangana	Komaram Bheem Asifabad	659	89.56	84.31	93.87	64.97	47.03	81.41
Telangana	Mahabubabad	660	85.30	78.76	90.77	81.52	71.12	90.20
Telangana	Mahabubnagar	661	92.07	87.12	95.80	83.73	72.32	91.85
Telangana	Mancherial	662	90.94	85.71	95.06	88.61	77.37	95.90
Telangana	Medak	663	80.09	73.08	86.02	74.72	51.11	91.85
Telangana	Medchal-Malkajgiri	664	91.33	86.32	95.40	82.58	74.09	89.62
Telangana	Nagarkurnool	665	88.55	82.85	93.07	90.83	81.40	96.68
Telangana	Nalgonda	666	92.71	87.93	96.18	78.62	70.29	86.25
Telangana	Nirmal	667	84.87	78.27	90.48	71.03	60.45	80.30
Telangana	Nizamabad	668	77.15	69.41	84.36	78.89	69.01	87.40
Telangana	Peddapalli	669	90.74	85.75	94.77	83.02	73.60	90.73
Telangana	Rajanna Sircilla	670	88.44	83.09	92.91	86.62	71.63	95.64
Telangana	Ranga Reddy	671	87.83	81.75	92.99	80.61	72.63	87.44
Telangana	Sangareddy	672	80.44	72.65	86.90	79.00	69.66	86.81
Telangana	Siddipet	673	78.62	71.04	85.00	80.10	65.45	90.79
Telangana	Suryapet	674	83.62	77.05	88.73	76.90	65.17	86.86
Telangana	Vikarabad	675	86.81	80.91	91.78	NA	NA	NA
Telangana	Wanaparthy	676	88.08	82.11	92.76	90.40	77.49	97.31
Telangana	Warangal Rural	677	81.90	75.23	87.88	90.11	82.10	95.51
Telangana	Warangal Urban	678	88.11	82.27	92.93	88.02	79.04	94.58
Telangana	Yadadri Bhuvanagiri	679	91.75	86.86	95.53	66.69	48.89	83.29
Tripura	Gomati	680	87.45	81.29	92.24	91.71	85.33	96.13

Tripura	Khowai	681	87.74	82.32	92.31	90.85	83.15	96.11
Tripura	North Tripura	682	93.38	89.02	96.72	91.17	85.94	95.10
Tripura	South Tripura	683	93.26	88.53	96.57	90.91	85.77	94.90
Tripura	Unakoti	684	94.45	90.55	97.18	88.60	82.39	93.57
Tripura	West Tripura	685	94.32	90.35	97.19	93.25	89.81	95.96
Uttar Pradesh	Amethi	686	91.64	87.14	95.09	77.52	69.49	84.89
Uttar Pradesh	Budaun	687	83.46	77.39	88.88	83.88	78.48	88.53
Uttar Pradesh	Ghaziabad	688	90.92	84.83	95.41	83.92	78.91	88.22
Uttar Pradesh	Hapur	689	90.35	85.92	94.13	77.28	69.80	83.93
Uttar Pradesh	Moradabad	690	90.20	85.02	94.33	87.99	83.73	91.76
Uttar Pradesh	Muzaffarnagar	691	89.88	84.92	93.79	78.90	71.29	85.17
Uttar Pradesh	Rae Bareli	692	80.44	74.21	86.05	78.34	71.97	83.86
Uttar Pradesh	Sambhal	693	80.72	74.69	85.98	84.80	78.71	89.97
Uttar Pradesh	Shamli	694	91.64	87.12	95.10	76.52	63.01	87.73
Uttar Pradesh	Sultanpur	695	88.65	83.41	93.01	79.46	65.45	89.91
West Bengal	Paschim Barddhaman	696	89.12	84.05	93.56	92.67	87.10	96.71
West Bengal	Purba Barddhaman	697	87.17	82.01	91.61	87.69	80.11	93.41
Tamil Nadu	Thiruvallur	698	95.28	91.43	97.90	83.87	78.63	88.37
Gujarat	Surat	699	94.74	91.50	97.30	79.57	74.15	84.61
Gujarat	Amreli	700	95.59	91.93	98.02	77.33	71.42	82.53
Chhattisgarh	Surajpur	701	93.07	88.86	96.41	90.02	84.37	94.35
Madhya Pradesh	Sidhi	702	82.15	75.98	87.37	67.67	61.00	74.27
Uttar Pradesh	Kheri	703	87.52	82.38	91.88	88.05	82.67	92.48
Tripura	Sepahijala	704	88.10	82.54	92.50	90.92	86.29	94.61
Karnataka	Udupi	705	82.43	76.49	88.08	86.77	81.07	91.62
Kerala	Kasaragod	706	89.59	84.98	93.37	88.28	83.58	92.34
Lakshadweep	Lakshadweep	707	92.42	87.79	96.05	81.35	75.19	87.06
Kerala	Kannur	708	95.47	91.83	97.89	95.24	91.60	97.69
Kerala	Alappuzha	709	97.06	93.91	98.96	94.56	90.95	97.21
Mizoram	Saiha	710	96.80	93.81	98.80	98.17	96.59	99.22
Andaman & Nicobar Islands	South Andaman	711	93.43	88.88	96.91	88.43	82.52	93.17
Tamil Nadu	Ramanathapuram	712	95.79	92.41	98.05	80.32	73.72	86.02
Andaman & Nicobar Islands	Nicobars	713	96.39	92.71	98.63	84.59	78.37	90.18
Puducherry	Karaikal	714	94.25	90.19	97.38	73.17	66.11	79.54
West Bengal	Haora	715	90.69	85.93	94.51	89.94	84.95	94.08
Odisha	Jagatsinghapur	716	83.57	76.79	89.44	86.32	80.71	91.03

Andaman & Nicobar Islands	North & Middle Andaman	717	96.88	93.49	98.92	96.09	92.64	98.32
Ladakh	Kargil	718	82.14	75.13	88.14	86.61	82.47	90.38
Bihar	Aurangabad	720	82.71	76.71	88.05	70.54	63.50	77.10
Maharashtra	Aurangabad	721	87.49	82.13	92.13	89.83	84.82	93.97
Dadra & Nagar Haveli And Daman & Diu	Diu	722	97.67	94.85	99.27	92.29	87.85	95.80
Puducherry	Mahe	723	95.21	91.19	97.93	84.73	80.01	89.23

Supplementary Table 6. District-level prevalence and 95% credible intervals of urban women's participation in household decision-making in India, 2016-2021

			2021			2016		
**State**	**District**	**District ID**	**Mean**	**95% CI**		**Mean**	**95% CI**	
Andhra Pradesh	Ananthapuramu	4	90.37	78.69	97.45	77.01	63.83	88.35
Andhra Pradesh	Annamayya	5	88.51	73.71	97.01	63.79	31.93	88.19
Andhra Pradesh	Bapatla	6	NA	NA	NA	86.05	67.61	96.74
Andhra Pradesh	Chittoor	7	NA	NA	NA	78.97	54.08	94.50
Andhra Pradesh	East Godavari	9	85.63	59.28	97.94	89.83	71.12	98.24
Andhra Pradesh	Eluru	10	89.42	64.21	98.99	78.71	68.28	87.67
Andhra Pradesh	Guntur	11	76.53	63.93	86.93	75.93	63.03	87.17
Andhra Pradesh	Kakinada	12	87.15	72.46	96.26	82.69	68.48	93.29
Andhra Pradesh	Krishna	13	86.36	63.08	97.76	NA	NA	NA
Andhra Pradesh	Kurnool	14	92.30	84.08	97.62	79.83	68.71	88.90
Andhra Pradesh	Nandyal	15	80.57	53.86	96.13	NA	NA	NA
Andhra Pradesh	Ntr	16	84.15	69.54	93.86	80.60	70.08	89.42
Andhra Pradesh	Palnadu	17	NA	NA	NA	79.26	66.86	89.57
Andhra Pradesh	Parvathipuram Manyam	18	82.78	59.29	96.33	NA	NA	NA
Andhra Pradesh	Prakasam	19	83.63	67.98	94.84	66.45	46.73	82.95
Andhra Pradesh	Sri Potti Sriramulu Nellore	20	79.39	65.10	90.07	82.35	70.26	91.68
Andhra Pradesh	Sri Sathya Sai	21	78.77	61.76	91.69	71.85	45.10	91.16
Andhra Pradesh	Srikakulam	22	93.16	82.01	98.56	70.67	51.60	85.57
Andhra Pradesh	Tirupati	23	74.62	53.79	91.24	76.03	61.00	87.69
Andhra Pradesh	Visakhapatnam	24	81.47	69.94	90.45	86.76	78.45	92.86
Andhra Pradesh	Vizianagaram	25	87.61	73.75	96.30	77.84	62.97	88.72
Andhra Pradesh	West Godavari	26	89.32	75.44	97.19	NA	NA	NA
Andhra Pradesh	Y.S.R.	27	85.00	75.12	92.75	82.12	73.57	89.11
West Bengal	Kolkata	29	94.91	90.91	97.75	92.75	88.12	96.30
Jammu & Kashmir	Kupwara	30	84.98	69.04	95.61	81.84	71.22	90.39
Jammu & Kashmir	Badgam	31	81.80	64.24	94.09	84.51	74.38	92.26
Ladakh	Leh	32	79.97	66.57	90.38	91.22	86.17	95.13
Jammu & Kashmir	Punch	33	86.49	65.04	97.75	91.00	80.50	97.13
Jammu & Kashmir	Rajouri	34	92.84	78.41	99.09	90.35	79.48	96.91

Jammu & Kashmir	Kathua	35	58.31	35.24	80.24	91.99	84.74	96.72
Jammu & Kashmir	Baramula	36	70.06	54.51	85.43	88.12	81.11	93.80
Jammu & Kashmir	Bandipore	37	82.04	67.25	93.75	75.07	63.47	85.48
Jammu & Kashmir	Srinagar	38	81.35	73.63	87.93	86.22	81.45	90.42
Jammu & Kashmir	Ganderbal	39	84.37	70.36	94.59	82.69	73.08	90.83
Jammu & Kashmir	Pulwama	40	72.02	55.38	87.67	92.58	85.55	97.08
Jammu & Kashmir	Shupiyan	41	94.25	80.56	99.42	93.74	85.25	98.29
Jammu & Kashmir	Anantnag	42	70.44	53.46	85.98	74.45	64.50	83.06
Jammu & Kashmir	Kulgam	43	92.14	80.03	98.23	92.61	87.18	96.35
Jammu & Kashmir	Doda	44	92.70	76.82	99.20	88.08	75.55	95.94
Jammu & Kashmir	Ramban	45	81.95	59.86	95.63	86.73	71.64	96.18
Jammu & Kashmir	Kishtwar	46	83.50	58.53	96.95	67.62	49.97	83.88
Jammu & Kashmir	Udhampur	47	85.11	69.97	95.14	87.87	80.19	93.49
Jammu & Kashmir	Reasi	48	84.19	61.16	96.79	87.73	74.81	95.86
Jammu & Kashmir	Jammu	49	84.47	75.77	91.36	89.42	84.18	93.61
Jammu & Kashmir	Samba	50	82.39	66.61	93.71	85.11	74.62	92.88
Himachal Pradesh	Chamba	51	96.97	88.18	99.76	91.71	80.67	97.71
Himachal Pradesh	Kangra	52	91.45	73.54	98.99	90.38	75.18	97.77
Himachal Pradesh	Kullu	54	96.93	87.64	99.81	93.60	86.30	97.91
Himachal Pradesh	Mandi	55	91.79	74.95	98.97	94.72	87.96	98.52
Himachal Pradesh	Hamirpur	56	95.47	82.95	99.62	92.90	82.83	98.35
Himachal Pradesh	Una	57	96.29	85.46	99.71	87.52	73.30	96.20
Himachal Pradesh	Bilaspur	58	96.51	86.63	99.72	93.82	83.76	98.53
Himachal Pradesh	Solan	59	96.51	88.48	99.49	91.47	84.76	96.03
Himachal Pradesh	Sirmaur	60	97.41	91.70	99.62	94.43	88.17	97.95
Himachal Pradesh	Shimla	61	95.69	88.47	99.05	93.32	86.37	97.46
Punjab	Kapurthala	63	93.57	87.36	97.54	83.48	75.21	90.26
Punjab	Jalandhar	64	95.81	91.07	98.49	93.40	87.91	97.17
Punjab	Hoshiarpur	65	93.73	85.78	98.16	91.16	82.10	96.89
Punjab	Shahid Bhagat Singh Nagar	66	95.53	89.45	98.87	92.97	87.40	96.66
Punjab	Fatehgarh Sahib	67	96.00	90.67	98.94	88.66	81.34	94.21
Punjab	Ludhiana	68	93.04	87.18	96.93	87.13	79.09	93.42
Punjab	Moga	69	93.87	85.61	98.29	93.70	86.96	97.94
Punjab	Muktsar	70	94.57	88.68	98.21	94.08	87.90	97.91
Punjab	Faridkot	71	90.19	81.40	95.86	84.93	77.19	91.34
Punjab	Bathinda	72	76.62	64.98	86.33	88.82	81.82	94.22

Punjab	Mansa	73	94.18	86.56	98.30	91.34	82.89	96.95
Punjab	Patiala	74	86.47	79.27	92.52	86.04	78.55	92.00
Punjab	Amritsar	75	93.93	88.36	97.62	94.67	89.98	97.71
Punjab	Tarn Taran	76	94.21	83.94	98.82	80.27	65.18	91.99
Punjab	Rupnagar	77	94.38	83.98	99.00	91.77	83.11	97.08
Punjab	Sahibzada Ajit Singh Nagar	78	94.47	88.36	97.98	88.64	81.59	94.22
Punjab	Sangrur	79	92.48	85.29	97.13	93.11	84.63	97.76
Punjab	Barnala	80	95.04	89.02	98.49	92.21	86.01	96.45
Chandigarh	Chandigarh	81	94.51	89.37	97.95	95.71	91.56	98.42
Uttarakhand	Uttarkashi	82	96.25	85.28	99.69	92.72	81.62	98.14
Uttarakhand	Chamoli	83	95.10	85.67	99.20	89.63	78.78	96.45
Uttarakhand	Rudraprayag	84	95.46	82.81	99.59	80.04	55.84	95.04
Uttarakhand	Tehri Garhwal	85	93.59	83.74	98.51	94.44	86.07	98.68
Uttarakhand	Dehradun	86	87.65	78.22	94.74	90.73	86.10	94.43
Uttarakhand	Garhwal	87	82.04	66.04	93.75	94.45	87.93	98.21
Uttarakhand	Pithoragarh	88	96.12	87.95	99.40	95.82	90.03	98.85
Uttarakhand	Bageshwar	89	95.67	84.16	99.64	94.27	81.38	99.24
Uttarakhand	Almora	90	94.64	82.61	99.15	89.29	75.64	96.87
Uttarakhand	Champawat	91	95.75	87.68	99.21	93.93	86.69	98.09
Uttarakhand	Nainital	92	93.88	87.86	97.75	89.88	85.33	93.74
Uttarakhand	Udham Singh Nagar	93	88.34	80.01	94.97	92.65	88.70	95.75
Uttarakhand	Hardwar	94	93.23	85.76	97.84	91.18	86.65	94.69
Haryana	Panchkula	95	93.65	85.65	98.02	79.34	69.25	87.58
Haryana	Ambala	96	87.01	79.43	93.16	89.08	81.94	94.42
Haryana	Yamunanagar	97	87.88	79.80	94.21	90.78	84.00	95.40
Haryana	Kurukshetra	98	95.60	89.44	98.85	65.63	54.13	76.51
Haryana	Kaithal	99	76.87	45.57	95.37	76.66	64.40	87.06
Haryana	Karnal	100	94.34	88.36	98.04	77.17	68.87	84.34
Haryana	Panipat	101	92.63	85.74	97.07	90.04	83.39	95.21
Haryana	Sonipat	102	88.04	78.25	94.91	84.39	76.13	91.02
Haryana	Jind	103	91.73	81.36	97.51	83.63	71.54	93.01
Haryana	Fatehabad	104	89.47	77.28	97.25	77.05	65.07	87.07
Haryana	Sirsa	105	NA	NA	NA	56.20	42.67	68.16
Haryana	Hisar	106	91.78	83.85	97.03	75.66	66.21	83.92
Haryana	Rohtak	107	89.96	82.09	95.52	73.39	64.33	81.43
Haryana	Jhajjar	108	87.28	74.02	95.88	79.54	66.38	90.34

Haryana	Mahendragarh	109	85.66	69.84	95.71	80.24	66.14	91.10
Haryana	Rewari	110	92.88	82.45	98.42	69.63	55.98	81.62
Haryana	Gurgaon	111	88.06	80.61	93.81	83.58	74.89	90.75
Haryana	Mewat	112	80.71	63.70	93.22	51.59	33.80	69.89
Haryana	Faridabad	113	90.30	84.87	94.66	71.38	62.97	79.38
Haryana	Palwal	114	92.74	84.45	97.83	86.87	76.31	94.30
Rajasthan	Ganganagar	115	93.42	86.25	97.81	76.83	66.97	85.58
Rajasthan	Hanumangarh	116	88.27	78.29	95.25	80.12	68.34	89.59
Rajasthan	Bikaner	117	89.44	82.96	94.47	91.87	87.90	94.93
Rajasthan	Churu	118	87.00	78.40	93.55	83.16	73.76	90.49
Rajasthan	Jhunjhunun	119	89.59	78.94	96.26	69.65	56.37	81.41
Rajasthan	Alwar	120	89.22	77.09	96.71	86.72	73.90	94.72
Rajasthan	Bharatpur	121	87.18	75.98	94.77	81.56	69.30	90.81
Rajasthan	Dhaulpur	122	92.11	83.43	97.52	90.52	80.46	96.54
Rajasthan	Karauli	123	89.01	77.27	96.74	86.97	76.38	94.49
Rajasthan	Sawai Madhopur	124	78.96	67.41	88.28	89.92	79.56	96.12
Rajasthan	Dausa	125	82.16	67.62	93.21	76.79	60.44	89.82
Rajasthan	Jaipur	126	93.28	87.69	97.03	90.33	85.41	94.27
Rajasthan	Sikar	127	90.05	80.17	96.37	80.82	70.39	89.21
Rajasthan	Nagaur	128	84.71	73.55	92.91	82.90	71.59	91.81
Rajasthan	Jodhpur	129	92.02	86.04	96.29	86.48	81.32	90.79
Rajasthan	Jaisalmer	130	77.41	60.14	90.22	76.30	59.93	88.99
Rajasthan	Barmer	131	87.21	68.54	97.36	74.26	46.44	93.27
Rajasthan	Jalor	132	94.41	81.79	99.37	79.08	64.06	90.76
Rajasthan	Sirohi	133	93.80	86.35	98.17	83.50	72.44	91.97
Rajasthan	Pali	134	86.82	75.70	94.62	88.77	79.74	95.46
Rajasthan	Ajmer	135	85.23	76.98	92.20	90.49	85.82	94.07
Rajasthan	Tonk	136	90.19	81.48	96.20	79.72	65.72	90.17
Rajasthan	Bundi	137	92.75	84.21	97.69	83.65	71.60	92.35
Rajasthan	Bhilwara	138	92.44	83.26	97.67	92.31	83.90	97.31
Rajasthan	Rajsamand	139	88.16	75.97	96.14	89.12	79.07	95.61
Rajasthan	Dungarpur	140	90.94	75.05	98.54	92.33	78.24	98.76
Rajasthan	Banswara	141	94.88	83.16	99.47	72.16	56.90	85.61
Rajasthan	Chittaurgarh	142	85.22	70.48	95.34	80.81	67.93	90.56
Rajasthan	Kota	143	87.70	80.85	93.18	82.64	76.75	87.81
Rajasthan	Baran	144	93.12	85.61	97.79	87.20	76.71	94.50

Rajasthan	Jhalawar	145	89.81	76.28	97.36	74.71	60.40	86.36
Rajasthan	Udaipur	146	93.69	86.74	97.86	79.38	64.98	90.98
Rajasthan	Pratapgarh	147	94.88	84.08	99.47	81.60	66.65	92.42
Uttar Pradesh	Saharanpur	148	93.86	87.58	97.92	87.42	82.49	91.70
Uttar Pradesh	Bijnor	149	87.28	75.32	95.54	82.66	72.40	90.57
Uttar Pradesh	Rampur	150	90.08	78.98	96.92	86.00	75.76	93.51
Uttar Pradesh	Jyotiba Phule Nagar	151	94.99	87.90	98.80	82.64	72.43	90.95
Uttar Pradesh	Meerut	152	90.46	84.00	95.24	82.67	77.96	86.74
Uttar Pradesh	Baghpat	153	91.67	82.91	97.31	89.15	79.15	95.67
Uttar Pradesh	Gautam Buddha Nagar	154	90.88	82.82	96.16	87.54	81.89	92.12
Uttar Pradesh	Bulandshahr	155	89.00	76.61	96.55	75.03	62.80	85.28
Uttar Pradesh	Aligarh	156	90.88	82.46	96.67	80.62	74.52	86.10
Uttar Pradesh	Mahamaya Nagar	157	87.42	76.74	94.79	74.48	61.16	86.43
Uttar Pradesh	Mathura	158	79.31	69.14	87.29	84.89	75.18	92.31
Uttar Pradesh	Agra	159	93.15	86.59	97.34	85.95	80.85	90.33
Uttar Pradesh	Firozabad	160	86.04	77.54	92.89	89.13	84.23	93.12
Uttar Pradesh	Mainpuri	161	92.29	81.51	98.16	87.85	78.12	95.03
Uttar Pradesh	Bareilly	162	88.13	78.36	94.93	90.03	85.79	93.71
Uttar Pradesh	Pilibhit	163	94.37	83.99	98.88	86.36	75.57	94.03
Uttar Pradesh	Shahjahanpur	164	87.30	77.81	94.42	91.22	81.96	96.89
Uttar Pradesh	Sitapur	165	85.41	69.93	95.68	84.71	69.11	94.72
Uttar Pradesh	Hardoi	166	89.79	76.52	97.28	62.87	43.54	80.15
Uttar Pradesh	Unnao	167	84.30	69.30	94.82	86.62	74.72	94.96
Uttar Pradesh	Lucknow	168	93.23	87.88	97.01	86.56	80.61	91.14
Uttar Pradesh	Farrukhabad	169	93.97	86.62	98.22	87.86	78.26	94.81
Uttar Pradesh	Kannauj	170	94.06	85.39	98.61	81.71	69.81	90.82
Uttar Pradesh	Etawah	171	88.16	78.11	95.25	90.56	81.42	96.46
Uttar Pradesh	Auraiya	172	94.57	85.88	98.88	51.31	36.95	66.01
Uttar Pradesh	Kanpur Dehat	173	84.45	59.51	97.59	89.63	77.24	96.92
Uttar Pradesh	Kanpur Nagar	174	83.88	76.39	89.91	87.62	82.28	91.98
Uttar Pradesh	Jalaun	175	92.25	83.63	97.31	80.21	67.91	90.04
Uttar Pradesh	Jhansi	176	92.89	87.12	96.88	86.83	81.28	91.32
Uttar Pradesh	Lalitpur	177	87.18	74.54	95.47	90.70	80.94	96.78
Uttar Pradesh	Hamirpur	178	93.33	85.25	97.95	74.94	60.37	86.50
Uttar Pradesh	Mahoba	179	93.15	84.52	97.96	86.81	75.65	94.79
Uttar Pradesh	Banda	180	85.23	68.71	95.54	90.30	80.86	96.55

Uttar Pradesh	Chitrakoot	181	81.71	56.32	96.12	87.88	75.51	95.64
Uttar Pradesh	Fatehpur	182	76.11	58.26	90.20	77.84	58.60	91.31
Uttar Pradesh	Pratapgarh	183	76.68	50.05	93.99	79.99	52.19	95.67
Uttar Pradesh	Kaushambi	184	95.02	83.45	99.46	79.20	61.92	91.28
Uttar Pradesh	Allahabad	185	85.89	69.90	95.93	88.89	80.10	95.31
Uttar Pradesh	Bara Banki	186	77.38	61.05	90.36	84.70	69.32	94.33
Uttar Pradesh	Faizabad	187	94.90	86.76	98.90	82.00	65.97	93.35
Uttar Pradesh	Ambedkar Nagar	188	94.82	86.03	99.05	80.38	66.24	91.57
Uttar Pradesh	Bahraich	189	82.60	55.67	96.88	90.90	80.40	97.17
Uttar Pradesh	Shrawasti	190	86.14	67.10	97.01	79.89	57.57	94.59
Chhattisgarh	Balrampur	191	59.56	31.05	83.05	NA	NA	NA
Uttar Pradesh	Gonda	192	92.09	69.84	99.35	83.53	61.05	96.32
Uttar Pradesh	Siddharthnagar	193	90.12	73.13	98.44	70.51	45.21	89.22
Uttar Pradesh	Basti	194	95.49	84.00	99.60	90.88	74.75	98.57
Uttar Pradesh	Sant Kabir Nagar	195	94.97	81.92	99.58	86.55	75.30	94.52
Uttar Pradesh	Maharajganj	196	93.07	75.32	99.40	85.95	64.97	97.05
Uttar Pradesh	Gorakhpur	197	91.64	80.83	97.91	81.44	68.73	91.02
Uttar Pradesh	Kushinagar	198	71.34	41.13	93.01	78.00	53.23	94.15
Uttar Pradesh	Deoria	199	90.50	78.11	97.47	72.68	56.14	86.59
Uttar Pradesh	Azamgarh	200	79.03	50.38	95.71	89.26	77.64	96.34
Uttar Pradesh	Mau	201	78.08	65.32	88.25	85.60	74.63	93.50
Uttar Pradesh	Ballia	202	93.89	78.44	99.43	60.56	42.91	77.52
Uttar Pradesh	Jaunpur	203	88.72	68.25	98.31	76.43	60.70	89.06
Uttar Pradesh	Ghazipur	204	93.83	78.71	99.41	88.24	75.87	95.94
Uttar Pradesh	Chandauli	205	89.02	72.76	97.71	80.09	67.11	90.22
Uttar Pradesh	Varanasi	206	85.89	77.35	92.35	85.11	79.39	89.92
Uttar Pradesh	Sant Ravidas Nagar	207	92.73	82.08	98.34	71.22	58.82	83.10
Uttar Pradesh	Mirzapur	208	93.87	83.78	98.73	76.58	59.50	89.89
Uttar Pradesh	Sonbhadra	209	93.05	80.44	98.71	85.78	75.19	94.01
Uttar Pradesh	Etah	210	88.85	76.17	96.46	91.03	81.68	96.67
Uttar Pradesh	Kanshiram Nagar	211	88.89	77.89	95.91	82.24	69.97	91.51
Bihar	Pashchim Champaran	212	69.46	44.14	89.46	76.77	59.52	89.34
Bihar	Purba Champaran	213	84.62	62.79	96.56	65.30	47.70	80.62
Bihar	Sheohar	214	82.62	59.59	96.09	65.27	40.31	87.21
Bihar	Sitamarhi	215	93.32	77.73	99.27	52.47	27.29	77.12
Bihar	Madhubani	216	94.29	80.72	99.46	73.12	51.91	89.89

Bihar	Supaul	217	93.57	77.27	99.46	78.38	55.43	93.63
Bihar	Araria	218	77.66	56.89	93.05	55.24	31.98	77.36
Bihar	Kishanganj	219	80.21	57.66	94.81	75.11	58.44	88.34
Bihar	Purnia	220	80.47	65.57	91.38	76.98	60.33	90.43
Bihar	Katihar	221	92.53	75.97	99.17	64.41	45.91	81.47
Bihar	Madhepura	222	94.50	81.60	99.44	66.05	39.94	88.02
Bihar	Saharsa	223	93.94	81.15	99.21	67.77	49.23	83.45
Bihar	Darbhanga	224	92.57	76.35	99.12	88.24	76.36	96.27
Bihar	Muzaffarpur	225	89.00	70.70	98.31	72.01	52.54	87.47
Bihar	Gopalganj	226	93.93	79.83	99.31	87.09	63.81	98.00
Bihar	Siwan	227	91.65	78.45	98.52	71.20	47.33	89.13
Bihar	Saran	228	85.05	61.97	97.40	81.31	64.48	92.45
Bihar	Vaishali	229	88.45	68.70	98.06	65.51	41.72	85.61
Bihar	Samastipur	230	93.43	79.04	99.29	76.44	49.91	93.44
Bihar	Begusarai	231	73.85	59.65	85.51	76.31	65.03	86.29
Bihar	Khagaria	232	88.84	69.85	98.14	86.21	66.77	96.81
Bihar	Bhagalpur	233	84.75	72.82	94.05	79.49	67.22	88.83
Bihar	Banka	234	80.69	55.92	95.77	86.64	68.85	96.86
Bihar	Munger	235	88.36	80.24	94.33	61.95	49.21	74.30
Bihar	Lakhisarai	236	82.48	67.35	93.05	72.95	59.12	84.85
Bihar	Sheikhpura	237	88.66	76.24	96.49	86.87	77.07	93.92
Bihar	Nalanda	238	83.88	69.22	94.51	72.32	58.24	84.21
Bihar	Patna	239	87.67	79.32	93.87	87.66	83.00	91.81
Bihar	Bhojpur	240	67.76	49.75	83.50	67.82	54.33	79.88
Bihar	Buxer	241	71.52	39.79	93.33	80.27	64.63	92.07
Bihar	Kaimur (Bhabua)	242	71.67	42.87	91.85	81.58	62.76	94.73
Bihar	Rohtas	243	80.73	65.92	92.07	73.25	59.63	85.03
Bihar	Gaya	244	80.47	67.44	91.32	88.24	77.28	95.53
Bihar	Nawada	245	87.80	67.66	97.85	89.50	78.17	96.42
Bihar	Jamui	246	93.02	77.75	99.23	72.48	55.62	86.77
Bihar	Jehanabad	247	72.27	54.52	87.20	86.50	71.20	95.79
Bihar	Arwal	248	79.27	54.32	95.26	81.42	67.12	92.03
Sikkim	North District	249	90.90	71.98	98.87	87.09	75.75	95.04
Sikkim	West District	250	78.57	51.99	95.08	95.80	88.30	99.14
Sikkim	South District	251	94.80	83.98	99.11	94.07	87.70	98.08
Sikkim	East District	252	87.91	77.51	95.24	95.09	92.10	97.38

Arunachal Pradesh	Tawang	253	72.14	55.95	86.68	93.51	85.23	98.14
Arunachal Pradesh	West Kameng	254	82.22	69.04	91.78	84.95	71.49	94.44
Arunachal Pradesh	East Kameng	255	92.94	82.56	98.54	90.33	80.25	96.59
Arunachal Pradesh	Papum Pare	256	79.66	70.58	87.30	89.59	83.96	93.87
Arunachal Pradesh	Upper Subansiri	257	90.47	78.48	97.64	89.81	80.18	96.31
Arunachal Pradesh	Upper Siang	258	91.50	78.55	97.98	90.54	81.18	96.53
Arunachal Pradesh	Changlang	259	90.33	78.23	97.30	92.10	81.12	98.01
Arunachal Pradesh	Lower Subansiri	260	93.25	82.82	98.52	80.22	62.28	92.96
Arunachal Pradesh	Dibang Valley	261	93.51	85.55	98.08	86.40	74.46	94.56
Arunachal Pradesh	Lower Dibang Valley	262	89.55	79.49	96.28	86.28	74.54	94.79
Arunachal Pradesh	Anjaw	263	94.87	81.74	99.51	91.99	77.05	98.80
Nagaland	Mon	264	98.88	95.54	99.89	93.64	82.82	98.72
Nagaland	Mokokchung	265	99.03	96.70	99.83	97.88	94.36	99.49
Nagaland	Zunheboto	266	99.04	96.01	99.91	98.14	94.71	99.59
Nagaland	Wokha	267	99.01	96.58	99.86	97.69	93.70	99.49
Nagaland	Dimapur	268	98.72	96.61	99.73	97.51	95.26	98.94
Nagaland	Phek	269	99.01	95.80	99.89	98.01	94.35	99.57
Nagaland	Tuensang	270	99.07	96.44	99.90	97.78	93.81	99.56
Nagaland	Longleng	271	98.91	95.81	99.90	98.10	94.52	99.61
Nagaland	Kiphire	272	99.09	96.73	99.89	98.00	94.37	99.57
Nagaland	Kohima	273	97.98	94.86	99.56	96.43	92.87	98.63
Nagaland	Peren	274	99.08	96.56	99.91	97.65	93.16	99.49
Manipur	Senapati	275	85.62	60.32	97.96	96.35	86.65	99.58
Manipur	Tamenglong	276	96.02	88.82	99.24	94.68	86.19	98.77
Manipur	Churachandpur	277	82.21	55.77	96.82	96.95	87.68	99.71
Manipur	Bishnupur	278	94.64	89.39	98.15	96.79	94.19	98.54
Manipur	Thoubal	279	96.25	91.69	98.84	96.36	93.50	98.23
Manipur	Imphal West	280	94.05	88.26	97.81	95.37	91.96	97.75
Manipur	Imphal East	281	94.22	88.10	98.11	96.49	93.46	98.49
Manipur	Ukhrul	282	94.46	83.76	99.10	97.27	92.66	99.40
Manipur	Chandel	283	96.69	90.01	99.43	97.38	92.21	99.52
Mizoram	Mamit	284	98.44	94.28	99.82	97.86	94.30	99.47
Mizoram	Kolasib	285	98.30	95.64	99.58	97.57	95.22	99.02
Mizoram	Aizawl	286	98.30	95.76	99.56	96.00	92.18	98.42
Mizoram	Champhai	287	97.88	94.59	99.48	94.73	91.06	97.36
Mizoram	Serchhip	288	97.74	94.07	99.48	97.68	95.51	99.07

Mizoram	Lunglei	289	98.37	95.26	99.67	96.57	93.67	98.48
Mizoram	Lawngtlai	290	98.07	93.36	99.76	95.70	89.67	98.88
Tripura	Dhalai	291	82.24	59.63	96.05	95.03	88.95	98.49
Meghalaya	South Garo Hills	292	79.57	49.97	96.97	83.13	68.95	93.44
Meghalaya	Ribhoi	293	96.17	85.27	99.65	87.18	71.33	96.35
Meghalaya	East Khasi Hills	294	90.99	83.98	96.31	94.29	90.46	96.99
Assam	Kokrajhar	295	97.00	88.47	99.74	93.44	81.73	98.88
Assam	Goalpara	296	88.70	76.35	96.43	68.02	49.82	83.66
Assam	Barpeta	297	97.20	89.02	99.78	92.16	82.21	97.87
Assam	Morigaon	298	95.76	84.00	99.64	88.83	76.84	96.40
Assam	Lakhimpur	299	96.62	86.28	99.72	90.48	78.65	97.15
Assam	Dhemaji	300	96.43	86.75	99.73	94.36	81.33	99.31
Assam	Tinsukia	301	94.86	86.24	98.89	91.80	83.17	97.20
Assam	Dibrugarh	302	95.93	87.93	99.28	94.68	87.83	98.34
Assam	Golaghat	303	93.16	78.48	99.20	95.76	88.32	99.08
Assam	Dima Hasao	304	96.20	90.82	98.98	92.16	84.75	96.90
Assam	Cachar	305	80.23	64.71	92.46	80.90	68.56	91.13
Assam	Karimganj	306	71.17	39.42	92.46	92.99	83.51	98.13
Assam	Hailakandi	307	93.33	77.07	99.32	91.23	79.84	97.42
Assam	Bongaigaon	308	96.15	88.45	99.42	76.36	60.95	88.38
Assam	Chirang	309	95.47	84.88	99.57	89.41	77.87	96.49
Assam	Kamrup	310	96.64	87.06	99.74	87.74	73.50	96.30
Assam	Kamrup Metropolitan	311	94.95	90.94	97.59	93.01	87.85	96.65
Assam	Nalbari	312	95.71	87.80	99.18	89.57	76.71	96.72
Assam	Baksa	313	NA	NA	NA	92.23	76.93	98.80
Assam	Darrang	314	96.78	88.03	99.73	88.10	71.01	97.51
Assam	Udalguri	315	90.72	71.08	98.78	89.79	72.05	98.40
West Bengal	Darjiling	316	95.22	89.77	98.41	87.43	79.96	93.43
West Bengal	Jalpaiguri	317	94.40	87.77	98.33	89.52	79.93	96.04
West Bengal	Koch Bihar	318	86.77	68.47	97.47	93.27	84.11	98.15
West Bengal	Uttar Dinajpur	319	94.59	86.11	98.83	79.50	65.15	91.68
West Bengal	Dakshin Dinajpur	320	95.58	88.14	99.11	87.03	74.45	95.55
West Bengal	Maldah	321	93.82	83.33	98.81	90.67	79.06	97.42
West Bengal	Murshidabad	322	95.32	87.69	99.03	90.67	81.99	96.34
West Bengal	Birbhum	323	94.42	84.80	98.79	92.31	82.50	97.67
West Bengal	Nadia	324	92.62	84.98	97.58	90.47	82.96	95.78

West Bengal	North Twenty Four Parganas	325	95.87	91.72	98.35	91.07	83.84	96.20
West Bengal	Hugli	326	89.60	80.98	95.60	90.85	83.66	95.90
West Bengal	Bankura	327	94.62	81.34	99.44	93.54	80.51	99.09
West Bengal	Puruliya	328	89.85	77.83	96.97	86.47	74.60	94.70
West Bengal	South Twenty Four Parganas	329	92.37	84.59	97.41	94.51	87.31	98.34
West Bengal	Paschim Medinipur	330	95.21	87.19	98.99	92.66	82.93	98.16
West Bengal	Purba Medinipur	331	91.12	79.15	97.67	88.65	77.59	95.88
Jharkhand	Garhwa	332	96.55	87.06	99.71	93.01	75.97	99.22
Jharkhand	Chatra	333	94.63	80.38	99.44	89.46	68.84	98.48
Jharkhand	Kodarma	334	94.89	88.23	98.57	80.07	69.92	88.85
Jharkhand	Giridih	335	94.86	81.90	99.49	91.44	81.09	97.55
Jharkhand	Deoghar	336	88.34	74.63	96.47	82.81	70.77	91.69
Jharkhand	Godda	337	93.58	78.69	99.46	87.70	67.07	98.05
Jharkhand	Sahibganj	338	91.51	80.83	97.78	81.86	65.94	93.38
Jharkhand	Pakur	339	95.51	82.82	99.60	88.45	76.64	96.09
Jharkhand	Dhanbad	340	94.67	90.21	97.68	77.14	70.06	83.26
Jharkhand	Bokaro	341	95.03	90.13	97.99	83.42	77.63	88.62
Jharkhand	Lohardaga	342	96.16	88.03	99.42	92.84	82.26	98.24
Jharkhand	Purbi Singhbhum	343	94.23	89.33	97.56	91.78	87.74	95.14
Jharkhand	Palamu	344	95.19	87.18	99.01	87.37	74.73	95.82
Jharkhand	Latehar	345	94.49	81.96	99.44	91.21	81.13	97.29
Jharkhand	Hazaribagh	346	94.48	84.58	98.92	83.46	72.80	91.84
Jharkhand	Ramgarh	347	92.05	85.91	96.49	94.00	90.35	96.75
Jharkhand	Dumka	348	93.33	80.40	99.08	94.62	83.42	99.25
Jharkhand	Jamtara	349	94.34	81.03	99.39	75.70	60.79	87.94
Jharkhand	Ranchi	350	91.86	84.94	96.53	90.64	85.68	94.60
Jharkhand	Khunti	351	84.13	62.92	96.60	89.37	75.49	97.00
Jharkhand	Gumla	352	93.46	79.19	99.33	88.92	70.39	98.16
Jharkhand	Simdega	353	95.49	83.15	99.64	95.41	88.54	98.91
Jharkhand	Pashchimi Singhbhum	354	88.01	74.21	96.39	88.49	77.44	95.86
Jharkhand	Saraikela-Kharsawan	355	72.84	60.42	84.37	92.73	84.63	97.47
Odisha	Bargarh	356	94.71	80.47	99.54	90.32	78.98	97.28
Odisha	Jharsuguda	357	95.91	90.81	98.75	84.90	79.80	89.47
Odisha	Sambalpur	358	94.80	88.36	98.48	84.44	74.50	92.27
Odisha	Debagarh	359	90.09	71.43	98.71	82.31	64.84	94.08
Odisha	Sundargarh	360	95.22	89.42	98.40	86.38	80.70	91.12

Odisha	Kendujhar	361	93.13	80.83	98.64	86.35	74.08	94.70
Odisha	Mayurbhanj	362	94.84	81.77	99.56	73.07	57.84	86.28
Odisha	Baleshwar	363	82.21	65.60	93.95	88.70	75.17	96.59
Odisha	Bhadrak	364	92.06	81.57	98.13	82.22	66.27	93.20
Odisha	Kendrapara	365	83.39	60.40	96.44	81.21	55.92	95.63
Odisha	Cuttack	366	86.17	74.62	95.00	85.18	74.31	92.97
Odisha	Jajapur	367	93.35	78.36	99.25	69.38	53.79	82.24
Odisha	Dhenkanal	368	90.32	71.66	98.81	90.63	79.57	97.13
Odisha	Anugul	369	90.13	76.97	97.48	90.49	82.03	96.05
Odisha	Nayagarh	370	83.80	61.40	96.71	83.79	71.06	93.32
Odisha	Khordha	371	90.54	83.30	95.71	83.77	78.18	88.77
Odisha	Puri	372	91.00	76.88	98.02	86.91	76.39	94.34
Odisha	Ganjam	373	85.93	74.88	93.87	82.35	70.18	91.74
Odisha	Gajapati	374	96.44	89.09	99.43	88.66	75.26	96.54
Odisha	Kandhamal	375	90.46	71.89	98.80	88.87	76.86	96.45
Odisha	Baudh	376	95.48	82.12	99.62	90.24	73.52	98.32
Odisha	Subarnapur	377	95.88	85.49	99.65	72.96	54.53	87.82
Odisha	Balangir	378	95.85	88.53	99.26	74.54	59.19	87.30
Odisha	Nuapada	379	92.64	77.12	99.16	62.00	38.09	83.14
Odisha	Kalahandi	380	94.10	80.56	99.44	83.32	67.73	93.89
Odisha	Rayagada	381	80.26	63.66	92.36	88.10	78.02	95.16
Odisha	Nabarangapur	382	95.34	82.39	99.59	85.40	71.41	95.20
Odisha	Koraput	383	96.01	88.52	99.27	83.16	71.71	92.01
Odisha	Malkangiri	384	95.24	83.32	99.56	65.39	48.14	80.93
Chhattisgarh	Koriya	385	93.08	85.96	97.71	91.72	87.66	95.04
Chhattisgarh	Jashpur	386	87.77	65.93	98.09	90.69	77.73	97.64
Chhattisgarh	Raigarh	387	95.07	85.52	99.13	92.30	83.68	97.37
Chhattisgarh	Korba	388	96.33	91.97	98.82	92.15	88.18	95.37
Chhattisgarh	Janjgir-Champa	389	92.88	81.75	98.52	91.73	82.23	97.25
Chhattisgarh	Kabeerdham	390	96.07	86.13	99.65	90.96	79.91	97.36
Chhattisgarh	Rajnandgaon	391	95.63	87.37	99.24	92.94	85.43	97.58
Chhattisgarh	Mahasamund	392	94.67	85.39	98.95	80.93	65.77	92.00
Chhattisgarh	Dhamtari	393	93.22	82.43	98.54	91.49	82.53	96.98
Chhattisgarh	Uttar Bastar Kanker	394	94.91	82.22	99.46	90.13	79.40	96.95
Chhattisgarh	Narayanpur	395	93.25	82.27	98.63	93.59	86.40	97.79
Chhattisgarh	Bijapur	396	95.89	88.00	99.29	94.83	87.15	98.78

Madhya Pradesh	Sheopur	397	85.77	71.69	95.11	87.37	75.94	95.22
Madhya Pradesh	Morena	398	93.65	85.72	98.24	87.13	79.16	93.52
Madhya Pradesh	Bhind	399	88.83	78.16	95.85	84.55	74.98	91.60
Madhya Pradesh	Gwalior	400	87.08	79.16	93.45	87.01	81.96	91.30
Madhya Pradesh	Datia	401	92.97	83.58	98.03	89.73	80.17	96.05
Madhya Pradesh	Shivpuri	402	91.92	74.08	99.20	85.25	74.39	93.29
Madhya Pradesh	Tikamgarh	403	89.76	59.43	99.27	89.15	79.13	95.80
Madhya Pradesh	Chhatarpur	404	86.89	76.76	94.55	87.39	76.62	94.76
Madhya Pradesh	Panna	405	81.36	63.85	93.56	57.92	41.56	73.61
Madhya Pradesh	Sagar	406	88.72	78.48	95.50	82.16	71.89	90.53
Madhya Pradesh	Damoh	407	82.54	70.27	92.51	76.64	63.79	87.30
Madhya Pradesh	Satna	408	93.18	81.42	98.75	88.65	78.12	95.51
Madhya Pradesh	Rewa	409	88.29	75.47	96.75	85.44	74.14	93.61
Madhya Pradesh	Umaria	410	73.02	57.27	87.18	84.26	73.19	92.93
Madhya Pradesh	Neemuch	411	88.83	79.95	95.40	86.51	78.58	92.96
Madhya Pradesh	Mandsaur	412	91.77	82.44	97.43	84.55	74.73	92.40
Madhya Pradesh	Ratlam	413	82.39	69.31	91.74	85.03	76.81	91.72
Madhya Pradesh	Ujjain	414	90.50	83.76	95.46	80.62	74.80	85.82
Madhya Pradesh	Dewas	415	91.58	83.70	96.66	86.02	76.67	93.41
Madhya Pradesh	Dhar	416	NA	NA	NA	79.34	67.42	88.67
Madhya Pradesh	Indore	417	87.59	81.24	92.78	89.44	85.97	92.67
Madhya Pradesh	Khargone (West Nimar)	418	78.83	64.84	90.62	91.69	82.66	96.94
Madhya Pradesh	Barwani	419	92.69	83.51	98.21	88.33	77.61	95.28
Madhya Pradesh	Rajgarh	420	65.88	35.86	89.60	76.06	64.23	86.40
Madhya Pradesh	Vidisha	421	85.56	71.53	94.75	79.88	66.87	89.97
Madhya Pradesh	Bhopal	422	91.08	74.22	98.40	84.67	78.65	89.78
Madhya Pradesh	Sehore	423	85.94	73.48	94.34	81.94	70.84	90.95
Madhya Pradesh	Raisen	424	NA	NA	NA	88.22	78.10	95.20
Madhya Pradesh	Betul	425	91.29	80.81	97.34	88.16	78.15	95.09
Madhya Pradesh	Harda	426	90.52	80.44	96.83	90.78	81.70	96.62
Madhya Pradesh	Hoshangabad	427	86.05	76.24	93.47	89.84	85.28	93.45
Madhya Pradesh	Katni	428	94.34	80.51	99.44	82.19	69.08	91.84
Madhya Pradesh	Jabalpur	429	NA	NA	NA	91.07	86.76	94.58
Madhya Pradesh	Narsimhapur	430	93.73	84.84	98.55	89.49	80.18	95.79
Madhya Pradesh	Dindori	431	75.20	42.08	95.62	91.25	72.35	98.86
Madhya Pradesh	Mandla	432	93.78	82.62	98.80	91.87	80.28	97.98

Madhya Pradesh	Chhindwara	433	87.67	76.63	95.62	90.16	82.09	95.78
Madhya Pradesh	Seoni	434	94.60	83.17	99.06	90.31	78.25	97.06
Madhya Pradesh	Balaghat	435	93.28	82.85	98.55	90.30	80.45	96.71
Madhya Pradesh	Guna	436	80.03	67.37	91.07	87.54	78.36	94.11
Madhya Pradesh	Ashoknagar	437	92.74	74.98	99.29	86.77	76.06	94.21
Madhya Pradesh	Shahdol	438	91.55	78.14	98.14	87.76	76.86	95.09
Madhya Pradesh	Anuppur	439	95.46	89.64	98.68	84.79	73.46	93.19
Madhya Pradesh	Singrauli	440	93.72	82.92	98.85	88.44	78.13	95.26
Madhya Pradesh	Jhabua	441	91.38	69.39	99.10	87.32	74.22	95.69
Madhya Pradesh	Alirajpur	442	69.99	44.37	89.48	86.91	74.14	95.33
Madhya Pradesh	Khandwa (East Nimar)	443	NA	NA	NA	90.29	80.37	96.27
Madhya Pradesh	Burhanpur	444	83.62	74.24	91.12	87.59	82.86	91.68
Gujarat	Kachchh	445	88.16	80.40	94.21	81.77	74.45	88.10
Gujarat	Banas Kantha	446	90.32	76.01	97.77	87.06	77.61	94.02
Gujarat	Patan	447	93.33	85.87	97.97	77.61	67.48	86.43
Gujarat	Mahesana	448	93.88	85.29	98.28	93.28	87.36	97.16
Gujarat	Gandhinagar	449	86.80	76.08	94.10	88.44	83.14	92.77
Gujarat	Porbandar	450	82.90	74.37	90.45	75.34	67.56	82.70
Gujarat	Anand	451	93.71	86.62	97.89	92.59	87.86	96.10
Gujarat	Dohad	452	78.65	59.58	92.77	91.46	81.05	97.42
Gujarat	Narmada	453	96.09	85.72	99.70	93.59	87.12	97.58
Gujarat	Bharuch	454	91.97	83.97	97.14	91.52	86.52	95.37
Gujarat	The Dangs	455	95.24	87.35	99.06	87.32	78.62	94.05
Gujarat	Navsari	456	94.82	88.28	98.45	93.10	88.58	96.46
Gujarat	Valsad	457	94.90	89.27	98.31	90.91	85.93	94.96
Gujarat	Tapi	458	96.26	86.17	99.66	83.65	71.42	92.42
Dadra & Nagar Haveli And Daman & Diu	Daman	459	97.78	95.13	99.36	86.73	79.91	91.98
Dadra & Nagar Haveli And Daman & Diu	Dadra & Nagar Haveli	460	93.89	88.41	97.62	78.20	68.95	86.23
Maharashtra	Nandurbar	461	95.00	86.97	98.89	72.82	55.23	88.05
Maharashtra	Dhule	462	80.17	68.32	89.74	90.80	81.67	96.65
Maharashtra	Jalgaon	463	91.51	82.92	96.84	89.14	82.21	94.38
Maharashtra	Buldana	464	70.96	56.13	84.63	93.19	85.95	97.66
Maharashtra	Akola	465	85.33	77.05	91.81	91.35	84.82	95.76
Maharashtra	Washim	466	80.85	65.05	92.76	94.42	87.57	98.31
Maharashtra	Amravati	467	85.10	75.93	92.38	91.90	85.77	96.18
Maharashtra	Wardha	468	87.01	78.44	93.94	84.69	76.89	91.30

Maharashtra	Nagpur	469	91.67	86.30	95.79	92.86	88.05	96.45
Maharashtra	Bhandara	470	92.58	78.44	98.58	93.54	85.62	97.95
Maharashtra	Gondiya	471	95.14	86.30	99.04	85.68	71.50	95.29
Maharashtra	Gadchiroli	472	80.55	63.18	93.57	93.60	84.00	98.51
Maharashtra	Chandrapur	473	88.09	79.59	94.49	91.26	85.30	95.75
Maharashtra	Yavatmal	474	83.44	71.70	92.56	93.78	86.30	98.00
Maharashtra	Nanded	475	93.31	84.94	98.15	80.20	68.29	89.91
Maharashtra	Hingoli	476	84.82	71.24	94.00	88.76	76.27	96.10
Maharashtra	Parbhani	477	81.83	72.05	90.12	88.65	81.16	94.40
Maharashtra	Jalna	478	78.35	59.69	91.94	89.52	80.18	95.96
Maharashtra	Nashik	479	90.53	81.52	96.44	91.04	84.53	95.70
Maharashtra	Mumbai Suburban	480	91.89	85.31	96.44	90.00	83.91	94.61
Maharashtra	Mumbai	481	94.07	89.58	97.21	89.47	81.69	95.04
Maharashtra	Raigarh	482	96.25	91.95	98.79	94.62	89.57	97.97
Maharashtra	Pune	483	91.03	84.37	95.72	93.37	87.76	97.25
Maharashtra	Ahmadnagar	484	87.14	73.24	95.93	80.64	67.24	90.96
Maharashtra	Bid	485	70.11	51.18	85.64	93.82	86.35	98.08
Maharashtra	Latur	486	78.42	65.11	89.66	90.09	81.08	96.11
Maharashtra	Osmanabad	487	93.13	83.92	98.33	92.51	81.91	98.18
Maharashtra	Solapur	488	92.63	84.61	97.39	89.02	82.69	94.06
Maharashtra	Satara	489	91.68	80.37	97.99	92.83	85.12	97.62
Maharashtra	Ratnagiri	490	95.28	86.30	99.21	93.25	82.39	98.42
Maharashtra	Sindhudurg	491	94.42	78.40	99.52	93.50	83.23	98.59
Maharashtra	Kolhapur	492	94.12	87.80	98.18	88.10	80.77	93.71
Maharashtra	Sangli	493	92.83	84.53	97.84	85.35	74.07	93.85
Karnataka	Belgaum	494	88.18	76.92	95.81	93.01	85.53	97.61
Karnataka	Bagalkot	495	78.22	65.45	88.40	64.30	54.01	74.47
Karnataka	Bijapur	496	81.32	66.02	92.30	77.56	64.56	88.07
Karnataka	Bidar	497	82.07	69.52	91.49	57.00	42.92	69.27
Karnataka	Raichur	498	82.27	68.53	92.31	66.79	54.13	78.52
Karnataka	Koppal	499	79.06	62.44	91.66	87.30	73.12	95.92
Karnataka	Gadag	500	68.13	57.97	77.92	66.21	55.32	75.62
Karnataka	Dharwad	501	84.51	75.90	91.34	82.04	73.51	89.56
Karnataka	Uttara Kannada	502	83.87	73.16	92.23	77.17	65.12	86.77
Karnataka	Haveri	503	85.75	73.47	94.19	85.39	73.74	93.77
Karnataka	Bellary	504	87.81	78.73	94.42	68.75	58.47	78.07

Karnataka	Chitradurga	505	93.14	83.28	98.35	87.70	77.42	94.72
Karnataka	Davanagere	506	77.04	65.01	87.78	72.46	61.67	81.99
Karnataka	Shimoga	507	79.67	69.66	87.59	78.83	68.38	87.68
Karnataka	Chikmagalur	508	85.38	73.27	94.15	87.64	76.17	95.22
Karnataka	Tumkur	509	79.19	62.61	91.60	85.97	74.64	94.23
Karnataka	Bangalore	510	89.43	83.70	94.21	85.40	79.12	90.93
Karnataka	Mandya	511	91.05	79.85	97.65	80.23	62.87	92.84
Karnataka	Hassan	512	81.48	68.88	91.56	81.54	68.73	91.56
Karnataka	Dakshina Kannada	513	79.91	69.46	88.71	85.33	77.16	91.97
Karnataka	Kodagu	514	88.83	76.22	96.77	72.62	57.50	85.81
Karnataka	Mysore	515	84.31	72.20	93.10	79.94	71.03	87.50
Karnataka	Chamarajanagar	516	56.11	36.66	74.28	83.85	72.33	92.67
Karnataka	Gulbarga	517	72.30	59.71	83.26	72.49	61.51	82.28
Karnataka	Yadgir	518	64.87	46.13	81.40	86.79	76.20	94.37
Karnataka	Kolar	519	86.66	76.12	94.16	82.59	74.98	89.69
Karnataka	Chikkaballapura	520	73.58	58.33	86.80	64.29	49.18	78.29
Karnataka	Bangalore Rural	521	87.75	76.78	95.33	73.73	59.83	84.83
Karnataka	Ramanagara	522	87.61	75.20	95.72	89.67	79.65	95.93
Goa	North Goa	523	85.14	76.15	92.29	96.44	94.00	98.19
Goa	South Goa	524	96.31	92.13	98.75	87.79	83.23	91.63
Kerala	Wayanad	525	94.54	79.09	99.56	90.26	78.63	97.10
Kerala	Kozhikode	526	89.93	83.92	94.45	94.98	91.00	97.82
Kerala	Malappuram	527	91.07	84.88	95.68	84.42	77.79	90.24
Kerala	Palakkad	528	93.17	83.37	98.41	87.96	81.75	93.02
Kerala	Thrissur	529	92.18	85.46	96.59	92.59	87.31	96.54
Kerala	Ernakulam	530	96.39	92.34	98.74	92.99	87.72	96.78
Kerala	Idukki	531	97.38	89.43	99.82	94.24	85.70	98.59
Kerala	Kottayam	532	95.28	89.01	98.68	92.50	85.99	96.97
Kerala	Pathanamthitta	533	96.23	88.19	99.48	94.90	87.40	98.67
Kerala	Kollam	534	97.13	93.25	99.20	92.20	86.90	96.06
Kerala	Thiruvananthapuram	535	94.37	88.31	97.98	92.44	86.62	96.42
Tamil Nadu	Chennai	536	92.20	86.87	96.14	85.67	80.00	90.44
Tamil Nadu	Kancheepuram	537	78.77	69.46	87.24	87.68	81.01	93.05
Tamil Nadu	Vellore	538	94.56	88.35	98.19	72.14	63.90	79.98
Tamil Nadu	Tiruvannamalai	539	93.64	84.31	98.58	84.91	75.64	92.31
Tamil Nadu	Viluppuram	540	95.27	86.73	99.16	83.17	71.51	91.78

Tamil Nadu	Salem	541	93.07	86.73	97.30	79.42	71.72	86.22
Tamil Nadu	Namakkal	542	91.62	84.54	96.76	85.36	78.23	91.38
Tamil Nadu	Erode	543	95.29	91.00	98.15	91.04	85.53	95.28
Tamil Nadu	The Nilgiris	544	96.32	92.51	98.67	73.83	65.53	81.56
Tamil Nadu	Dindigul	545	95.22	90.31	98.28	87.67	81.17	92.73
Tamil Nadu	Karur	546	92.84	85.31	97.53	86.10	79.34	91.78
Tamil Nadu	Tiruchirappalli	547	92.32	84.20	97.11	82.28	74.98	88.89
Tamil Nadu	Perambalur	548	95.54	85.80	99.32	84.36	74.80	91.85
Tamil Nadu	Ariyalur	549	88.91	74.68	97.29	77.82	64.93	88.91
Tamil Nadu	Cuddalore	550	93.37	84.68	98.15	83.33	76.35	89.71
Tamil Nadu	Nagapattinam	551	90.06	79.74	96.95	85.28	74.65	93.13
Tamil Nadu	Thiruvarur	552	93.94	84.92	98.57	90.59	82.29	96.22
Tamil Nadu	Thanjavur	553	95.16	89.43	98.54	84.81	77.84	90.58
Tamil Nadu	Pudukkottai	554	87.94	74.14	96.60	81.20	70.48	89.71
Tamil Nadu	Sivaganga	555	95.35	89.35	98.63	87.49	81.13	92.76
Tamil Nadu	Madurai	556	93.63	88.22	97.32	87.95	81.58	92.85
Tamil Nadu	Theni	557	92.91	87.52	96.89	85.66	78.65	91.36
Tamil Nadu	Virudhunagar	558	88.95	80.06	95.21	74.63	65.23	83.07
Tamil Nadu	Thoothukkudi	559	91.69	83.96	96.63	80.03	72.32	86.72
Tamil Nadu	Tirunelveli	560	93.59	86.47	97.60	87.69	80.72	93.07
Tamil Nadu	Kanniyakumari	561	94.76	90.28	97.66	86.26	80.64	91.10
Tamil Nadu	Dharmapuri	562	95.72	86.59	99.34	80.15	70.73	88.23
Tamil Nadu	Krishnagiri	563	96.46	91.41	99.16	79.04	68.51	87.85
Tamil Nadu	Coimbatore	564	95.25	91.15	98.09	80.73	74.28	86.69
Tamil Nadu	Tiruppur	565	93.46	87.99	97.17	80.23	73.02	86.65
Puducherry	Yanam	566	92.94	88.42	96.32	80.17	74.62	85.29
Puducherry	Puducherry	567	96.44	92.35	98.79	88.39	82.26	93.35
Arunachal Pradesh	East Siang	568	91.82	83.97	96.80	80.54	62.64	92.70
Arunachal Pradesh	Kurung Kumey	570	80.07	54.62	96.05	92.70	78.08	98.98
Arunachal Pradesh	Lohit	571	89.69	80.42	95.63	92.61	81.47	98.12
Arunachal Pradesh	Longding	572	95.00	82.63	99.51	NA	NA	NA
Arunachal Pradesh	Namsai	573	86.10	73.44	95.35	58.94	30.39	83.61
Arunachal Pradesh	Siang	574	92.49	81.80	98.12	92.83	82.85	98.16
Arunachal Pradesh	Tirap	575	88.87	79.82	95.21	92.67	84.06	97.74
Arunachal Pradesh	West Siang	576	90.62	78.32	97.25	75.53	59.05	88.92
Assam	Biswanath	577	95.33	82.68	99.61	NA	NA	NA

Assam	Charaideo	578	96.12	86.67	99.65	94.33	81.45	99.27
Assam	Dhubri	579	95.59	87.47	99.25	90.85	79.38	97.39
Assam	Hojai	580	95.06	88.01	98.76	91.94	71.33	99.05
Assam	Jorhat	581	95.66	88.79	98.98	95.34	89.06	98.74
Assam	Karbi Anglong	582	86.60	72.31	95.89	94.17	84.98	98.70
Assam	Nagaon	584	87.71	74.53	96.11	93.90	79.99	99.20
Assam	Sivasagar	585	93.55	82.52	98.77	92.85	75.57	99.11
Assam	Sonitpur	586	96.47	90.00	99.41	93.09	83.62	98.16
Assam	South Salmara Mancachar	587	96.41	86.02	99.71	NA	NA	NA
Assam	West Karbi Anglong	588	95.88	85.72	99.64	NA	NA	NA
Chhattisgarh	Balod	589	93.80	83.12	98.85	83.65	59.97	96.61
Chhattisgarh	Baloda Bazar	590	95.70	88.14	99.20	88.96	69.10	98.18
Uttar Pradesh	Balrampur	591	78.83	55.21	94.18	70.37	53.94	84.40
Chhattisgarh	Bastar	592	95.81	88.69	99.22	NA	NA	NA
Chhattisgarh	Bemetara	593	95.41	82.75	99.67	89.27	70.25	98.27
Chhattisgarh	Bilaspur	594	94.85	88.33	98.57	91.56	83.65	96.92
Chhattisgarh	Dantewada	595	90.75	80.99	96.97	88.38	68.20	98.00
Chhattisgarh	Durg	596	93.03	87.64	96.82	92.16	87.86	95.51
Chhattisgarh	Gariyaband	597	96.75	87.96	99.73	NA	NA	NA
Chhattisgarh	Kodagaon	598	96.32	85.12	99.71	93.59	85.08	98.15
Chhattisgarh	Mungeli	599	91.32	73.98	98.86	83.08	60.23	96.01
Chhattisgarh	Raipur	600	95.56	91.30	98.21	92.66	88.62	95.71
Chhattisgarh	Sukma	601	96.00	88.73	99.33	90.83	78.91	97.46
Chhattisgarh	Surguja	602	96.00	87.94	99.37	94.48	83.26	99.19
Nct Of Delhi	Central	603	87.14	80.69	92.49	68.34	60.57	75.45
Nct Of Delhi	East	604	92.65	88.17	96.11	75.42	64.20	85.61
Nct Of Delhi	New Delhi	605	88.73	83.19	93.14	68.37	59.17	76.46
Nct Of Delhi	North	606	87.89	81.50	93.06	74.98	60.84	86.19
Nct Of Delhi	North East	607	92.18	87.50	95.86	75.76	67.67	83.15
Nct Of Delhi	North West	608	92.48	87.70	96.02	77.31	64.66	87.69
Nct Of Delhi	Shahdara	609	92.98	88.42	96.35	72.98	59.79	84.89
Nct Of Delhi	South	610	95.77	92.32	98.19	81.59	70.64	90.27
Nct Of Delhi	South East	611	94.01	89.98	96.94	65.72	52.46	77.24
Nct Of Delhi	South West	612	93.50	88.85	96.79	82.55	72.95	90.30
Nct Of Delhi	West	613	93.55	89.38	96.66	78.19	70.65	85.01
Gujarat	Ahmadabad	614	95.51	91.77	98.15	91.07	86.66	94.56

Gujarat	Aravali	615	95.01	88.06	98.84	91.42	75.83	98.30
Gujarat	Bhavnagar	616	95.29	89.22	98.53	82.92	74.58	89.75
Gujarat	Botad	617	95.36	89.44	98.63	74.01	61.28	84.78
Gujarat	Chhota Udaipur	618	63.55	35.41	86.31	NA	NA	NA
Gujarat	Devbhumi Dwarka	619	91.89	85.00	96.63	92.61	79.82	98.69
Gujarat	Gir Somnath	620	97.11	92.19	99.37	89.10	78.19	95.92
Gujarat	Jamnagar	621	91.29	84.94	95.89	80.64	73.74	86.90
Gujarat	Junagadh	622	89.67	82.19	95.09	79.76	71.59	87.40
Gujarat	Kheda	623	89.91	80.80	96.28	89.01	81.34	94.37
Gujarat	Mahisagar	624	90.08	71.19	98.58	NA	NA	NA
Gujarat	Morbi	625	91.00	83.58	96.19	90.54	78.57	97.10
Gujarat	Panch Mahals	626	95.99	88.62	99.27	82.35	71.92	90.84
Gujarat	Rajkot	627	96.89	93.59	98.92	86.39	79.70	92.17
Gujarat	Sabar Kantha	628	96.36	89.38	99.37	92.46	84.26	97.37
Gujarat	Surendranagar	629	82.94	71.31	92.00	92.04	86.51	95.99
Gujarat	Vadodara	630	87.90	81.92	92.94	90.55	84.94	94.57
Haryana	Bhiwani	631	79.09	65.73	90.28	70.67	54.72	84.10
Haryana	Charkhi Dadri	632	80.85	45.91	97.84	89.34	73.56	97.97
Madhya Pradesh	Agar Malwa	633	88.61	74.15	97.07	80.86	59.22	94.64
Madhya Pradesh	Shajapur	634	76.54	60.31	90.37	78.60	65.05	89.70
Maharashtra	Palghar	635	94.79	89.18	98.10	92.79	78.73	98.98
Maharashtra	Thane	636	93.75	87.83	97.51	84.18	75.55	91.06
Meghalaya	East Garo Hills	637	95.13	86.95	99.05	93.08	83.46	98.13
Meghalaya	North Garo Hills	639	89.00	75.63	96.96	NA	NA	NA
Meghalaya	West Garo Hills	642	95.13	87.13	99.05	94.72	86.25	98.69
Meghalaya	West Jaintia Hills	643	90.68	71.75	98.97	93.23	82.67	98.47
Meghalaya	West Khasi Hills	644	97.34	92.30	99.54	96.54	89.80	99.31
Punjab	Fazilka	645	87.40	76.64	95.49	92.40	81.35	98.18
Punjab	Firozpur	646	96.01	91.00	98.70	90.21	72.11	98.36
Punjab	Gurdaspur	647	96.32	90.66	99.20	88.91	79.47	95.35
Punjab	Pathankot	648	88.14	79.42	94.85	NA	NA	NA
Telangana	Adilabad	649	86.68	74.37	94.78	NA	NA	NA
Telangana	Bhadradri Kothagudem	650	81.20	69.05	91.25	83.18	67.31	94.34
Telangana	Hyderabad	651	91.42	85.74	95.54	79.74	72.44	86.21
Telangana	Jagitial	652	91.35	82.00	96.95	NA	NA	NA
Telangana	Jangoan	653	90.95	78.51	97.70	NA	NA	NA

Telangana	Jayashankar Bhupalapally	654	90.06	68.00	98.92	NA	NA	NA
Telangana	Jogulamba Gadwal	655	88.91	70.85	98.07	NA	NA	NA
Telangana	Kamareddy	656	77.06	59.90	90.29	NA	NA	NA
Telangana	Karimnagar	657	86.86	76.94	93.89	NA	NA	NA
Telangana	Khammam	658	86.36	74.66	94.51	70.66	44.63	89.74
Telangana	Komaram Bheem Asifabad	659	87.13	71.82	96.53	NA	NA	NA
Telangana	Mahabubabad	660	79.18	52.64	95.55	NA	NA	NA
Telangana	Mahabubnagar	661	94.63	87.61	98.58	83.07	66.41	93.92
Telangana	Mancherial	662	90.16	81.29	96.12	89.17	75.83	96.86
Telangana	Medak	663	75.20	46.12	94.00	74.72	51.11	91.85
Telangana	Medchal-Malkajgiri	664	91.00	85.72	95.27	81.53	72.12	89.17
Telangana	Nagarkurnool	665	81.89	58.61	96.43	NA	NA	NA
Telangana	Nalgonda	666	95.27	88.58	98.68	79.70	66.23	89.78
Telangana	Nirmal	667	78.63	62.79	90.88	78.28	60.41	91.19
Telangana	Nizamabad	668	74.89	61.81	86.24	78.44	65.52	89.38
Telangana	Peddapalli	669	90.59	83.18	96.06	76.11	59.44	89.00
Telangana	Rajanna Sircilla	670	87.83	76.50	95.58	90.95	72.63	98.57
Telangana	Ranga Reddy	671	88.39	80.72	94.46	78.05	64.04	89.13
Telangana	Sangareddy	672	84.97	75.62	92.24	78.05	59.98	91.09
Telangana	Siddipet	673	84.35	70.17	94.50	NA	NA	NA
Telangana	Suryapet	674	87.01	71.67	96.14	NA	NA	NA
Telangana	Vikarabad	675	84.77	69.58	94.95	NA	NA	NA
Telangana	Wanaparthy	676	91.51	80.29	97.75	NA	NA	NA
Telangana	Warangal Rural	677	93.50	79.85	99.17	91.29	74.06	98.68
Telangana	Warangal Urban	678	89.08	82.84	94.02	91.84	82.93	97.23
Telangana	Yadadri Bhuvanagiri	679	91.14	77.96	97.95	NA	NA	NA
Tripura	Gomati	680	93.31	81.98	98.67	93.52	78.51	99.11
Tripura	Khowai	681	94.05	84.58	98.73	NA	NA	NA
Tripura	North Tripura	682	92.07	82.16	97.58	94.00	88.00	97.85
Tripura	South Tripura	683	95.02	80.29	99.57	91.13	78.73	97.71
Tripura	Unakoti	684	95.96	85.64	99.64	NA	NA	NA
Tripura	West Tripura	685	93.80	88.56	97.30	93.01	89.18	96.00
Uttar Pradesh	Amethi	686	93.73	76.64	99.45	67.90	38.78	89.19
Uttar Pradesh	Budaun	687	87.40	74.17	95.65	78.83	66.82	88.48
Uttar Pradesh	Ghaziabad	688	90.26	83.33	95.22	87.35	82.05	91.94
Uttar Pradesh	Hapur	689	87.07	77.23	94.03	81.02	59.58	95.01

Uttar Pradesh	Moradabad	690	89.35	81.26	95.09	87.06	81.35	92.23
Uttar Pradesh	Muzaffarnagar	691	88.94	80.56	95.02	81.57	69.56	90.53
Uttar Pradesh	Rae Bareli	692	92.57	77.20	99.08	88.32	76.39	95.88
Uttar Pradesh	Sambhal	693	82.32	70.36	91.97	83.49	74.06	90.98
Uttar Pradesh	Shamli	694	92.84	85.05	97.75	82.87	59.09	96.22
Uttar Pradesh	Sultanpur	695	93.83	80.24	99.33	NA	NA	NA
West Bengal	Paschim Barddhaman	696	87.99	82.26	92.99	91.96	84.98	96.82
West Bengal	Purba Barddhaman	697	89.79	77.79	97.08	87.24	73.57	95.80
Tamil Nadu	Thiruvallur	698	95.46	90.97	98.28	87.05	80.64	92.17
Gujarat	Surat	699	94.19	90.38	97.11	80.46	74.90	85.64
Gujarat	Amreli	700	94.83	87.38	98.68	74.69	63.62	84.15
Chhattisgarh	Surajpur	701	92.11	75.74	99.08	94.24	81.39	99.26
Madhya Pradesh	Sidhi	702	93.03	78.43	99.11	77.39	61.66	89.69
Uttar Pradesh	Kheri	703	93.62	82.99	98.64	88.54	74.71	96.62
Tripura	Sepahijala	704	94.04	80.04	99.41	95.96	87.71	99.40
Karnataka	Udupi	705	82.11	72.43	90.78	87.28	77.66	94.31
Kerala	Kasaragod	706	95.63	90.72	98.56	89.34	83.52	93.93
Lakshadweep	Lakshadweep	707	91.94	86.77	96.01	80.42	73.99	86.45
Kerala	Kannur	708	95.23	90.99	98.11	95.37	91.23	98.00
Kerala	Alappuzha	709	96.94	92.70	99.09	94.72	90.41	97.65
Mizoram	Saiha	710	96.89	93.02	99.13	98.16	96.17	99.31
Andaman & Nicobar Islands	South Andaman	711	93.95	88.66	97.58	88.76	81.42	94.59
Tamil Nadu	Ramanathapuram	712	94.83	88.46	98.39	77.70	68.57	85.93
Puducherry	Karaikal	714	96.19	91.58	98.83	74.95	66.00	82.93
West Bengal	Haora	715	92.41	87.35	96.29	90.95	84.39	95.73
Odisha	Jagatsinghapur	716	73.25	45.96	92.67	88.19	73.43	96.48
Andaman & Nicobar Islands	North & Middle Andaman	717	NA	NA	NA	96.25	86.98	99.59
Ladakh	Kargil	718	87.65	71.65	96.91	82.49	72.19	91.13
Bihar	Aurangabad	720	83.44	62.64	96.30	77.66	61.29	90.31
Maharashtra	Aurangabad	721	88.52	80.61	94.77	90.77	84.58	95.25
Dadra & Nagar Haveli And Daman & Diu	Diu	722	97.73	94.21	99.45	90.85	84.75	95.31
Puducherry	Mahe	723	95.21	91.19	97.93	84.73	80.01	89.23

Note. Districts missing data for both years were excluded from this table.

Supplementary Table 7. District-level prevalence and 95% credible intervals of rural women's participation in household decision-making in India, 2016-2021

			2021			2016		
**State**	**District**	**District ID**	**Mean**	**95% CI**		**Mean**	**95% CI**	
Andhra Pradesh	Alluri Sitharama Raju	2	80.67	56.54	95.90	87.44	76.91	94.73
Andhra Pradesh	Anakapalli	3	85.59	76.53	92.98	85.27	70.71	95.01
Andhra Pradesh	Ananthapuramu	4	84.16	74.82	91.56	83.99	75.10	91.05
Andhra Pradesh	Annamayya	5	76.87	67.80	85.54	86.37	77.77	92.83
Andhra Pradesh	Bapatla	6	86.77	77.94	93.76	79.43	68.62	88.25
Andhra Pradesh	Chittoor	7	82.26	72.07	91.20	85.24	74.71	93.24
Andhra Pradesh	Dr. B.R. Ambedkar Konaseema	8	93.62	85.47	98.28	84.19	67.81	94.62
Andhra Pradesh	East Godavari	9	82.71	74.51	89.75	85.84	76.98	92.62
Andhra Pradesh	Eluru	10	89.15	81.32	95.07	79.53	71.03	86.83
Andhra Pradesh	Guntur	11	82.00	69.99	91.41	70.86	51.83	86.33
Andhra Pradesh	Kakinada	12	88.33	78.24	95.55	76.89	63.22	87.73
Andhra Pradesh	Krishna	13	86.09	74.19	94.54	81.44	68.83	91.25
Andhra Pradesh	Kurnool	14	79.73	68.67	89.33	60.70	47.24	73.31
Andhra Pradesh	Nandyal	15	93.84	88.03	97.56	73.58	64.18	82.51
Andhra Pradesh	Ntr	16	74.40	60.45	86.16	71.83	45.47	90.75
Andhra Pradesh	Palnadu	17	70.89	56.49	82.97	71.86	58.12	84.70
Andhra Pradesh	Parvathipuram Manyam	18	90.22	80.16	96.55	78.65	66.00	89.24
Andhra Pradesh	Prakasam	19	85.73	76.40	92.78	77.74	65.41	88.20
Andhra Pradesh	Sri Potti Sriramulu Nellore	20	81.09	72.72	88.43	80.81	73.38	87.09
Andhra Pradesh	Sri Sathya Sai	21	85.09	75.15	92.87	86.29	71.10	95.74
Andhra Pradesh	Srikakulam	22	88.33	80.57	94.25	72.21	62.90	80.73
Andhra Pradesh	Tirupati	23	84.00	74.50	91.78	90.68	84.20	95.39
Andhra Pradesh	Visakhapatnam	24	82.82	54.16	97.31	85.17	70.58	95.03
Andhra Pradesh	Vizianagaram	25	89.91	83.50	94.68	77.09	68.03	85.11
Andhra Pradesh	West Godavari	26	81.78	66.26	93.34	80.89	68.00	91.17
Andhra Pradesh	Y.S.R.	27	80.47	71.59	88.24	90.32	81.74	96.01
Jammu & Kashmir	Kupwara	30	88.60	82.85	93.25	82.21	76.96	86.73
Jammu & Kashmir	Badgam	31	73.83	66.38	80.83	79.78	74.65	84.59
Ladakh	Leh	32	81.82	74.17	88.73	88.40	83.32	92.83
Jammu & Kashmir	Punch	33	83.94	77.03	90.07	85.82	81.82	89.41
Jammu & Kashmir	Rajouri	34	80.16	73.77	86.02	83.53	78.71	87.63

Jammu & Kashmir	Kathua	35	48.61	39.99	56.82	84.11	79.47	88.31
Jammu & Kashmir	Baramula	36	77.70	70.45	84.52	86.82	82.30	90.80
Jammu & Kashmir	Bandipore	37	79.72	72.06	86.64	77.33	71.94	82.27
Jammu & Kashmir	Srinagar	38	NA	NA	NA	65.32	47.09	81.47
Jammu & Kashmir	Ganderbal	39	82.19	75.94	87.77	73.54	67.81	79.00
Jammu & Kashmir	Pulwama	40	89.64	83.63	94.13	91.69	87.77	94.84
Jammu & Kashmir	Shupiyan	41	89.64	83.82	94.09	90.93	87.14	94.10
Jammu & Kashmir	Anantnag	42	84.99	77.14	91.75	79.87	74.10	84.88
Jammu & Kashmir	Kulgam	43	85.82	78.77	91.59	87.54	82.81	91.58
Jammu & Kashmir	Doda	44	88.24	82.55	93.05	65.68	59.51	71.83
Jammu & Kashmir	Ramban	45	82.72	76.17	88.48	74.50	68.71	79.81
Jammu & Kashmir	Kishtwar	46	76.49	68.90	83.19	67.00	60.63	72.82
Jammu & Kashmir	Udhampur	47	90.38	84.73	94.80	86.11	81.62	90.00
Jammu & Kashmir	Reasi	48	88.20	82.19	93.03	87.75	83.60	91.30
Jammu & Kashmir	Jammu	49	87.14	79.25	93.52	85.84	80.24	90.54
Jammu & Kashmir	Samba	50	77.00	70.12	83.59	88.22	84.05	92.08
Himachal Pradesh	Chamba	51	95.69	92.23	98.09	89.03	84.71	92.83
Himachal Pradesh	Kangra	52	93.07	88.41	96.54	86.77	81.94	90.72
Himachal Pradesh	Lahul & Spiti	53	93.76	89.18	97.04	92.24	88.09	95.44
Himachal Pradesh	Kullu	54	96.42	93.26	98.63	94.22	91.19	96.56
Himachal Pradesh	Mandi	55	92.68	88.15	96.13	94.05	90.87	96.47
Himachal Pradesh	Hamirpur	56	91.90	86.68	96.00	93.94	90.75	96.48
Himachal Pradesh	Una	57	94.92	90.56	97.82	88.48	83.94	92.43
Himachal Pradesh	Bilaspur	58	94.66	90.75	97.39	91.51	87.58	94.70
Himachal Pradesh	Solan	59	95.75	91.91	98.25	89.06	84.71	92.77
Himachal Pradesh	Sirmaur	60	97.26	94.74	98.94	90.90	87.27	94.00
Himachal Pradesh	Shimla	61	95.77	92.17	98.18	91.41	87.20	94.72
Himachal Pradesh	Kinnaur	62	94.01	89.89	97.18	92.19	88.31	95.31
Punjab	Kapurthala	63	88.47	82.11	93.78	88.77	81.20	94.17
Punjab	Jalandhar	64	93.86	88.37	97.69	93.02	87.45	96.92
Punjab	Hoshiarpur	65	94.74	90.44	97.67	91.50	85.80	95.48
Punjab	Shahid Bhagat Singh Nagar	66	95.44	91.29	98.10	89.88	82.98	94.94
Punjab	Fatehgarh Sahib	67	94.45	89.89	97.57	88.07	81.30	93.52
Punjab	Ludhiana	68	82.09	71.94	90.99	91.58	85.30	96.12
Punjab	Moga	69	91.89	86.66	95.74	88.17	81.98	93.02
Punjab	Muktsar	70	95.48	91.42	98.08	93.14	88.58	96.42

Punjab	Faridkot	71	79.96	71.74	86.76	88.16	80.93	93.72
Punjab	Bathinda	72	80.64	72.97	87.49	89.05	82.48	94.24
Punjab	Mansa	73	90.27	84.60	94.72	88.58	82.42	93.49
Punjab	Patiala	74	83.55	76.11	90.30	89.51	82.60	94.85
Punjab	Amritsar	75	95.39	91.00	98.21	92.09	86.69	96.09
Punjab	Tarn Taran	76	84.56	78.12	90.15	91.14	86.38	95.04
Punjab	Rupnagar	77	88.91	82.03	93.97	91.53	86.04	95.55
Punjab	Sahibzada Ajit Singh Nagar	78	94.89	89.97	98.05	89.32	82.24	94.40
Punjab	Sangrur	79	92.17	87.11	96.01	91.73	86.63	95.63
Punjab	Barnala	80	94.13	89.74	97.40	90.58	84.21	95.39
Chandigarh	Chandigarh	81	NA	NA	NA	90.02	72.21	98.39
Uttarakhand	Uttarkashi	82	94.75	90.83	97.49	88.61	83.28	92.96
Uttarakhand	Chamoli	83	93.22	88.43	96.70	90.56	85.07	94.87
Uttarakhand	Rudraprayag	84	92.97	88.24	96.42	80.38	73.30	86.61
Uttarakhand	Tehri Garhwal	85	90.43	84.85	94.54	91.58	86.85	95.29
Uttarakhand	Dehradun	86	89.77	82.42	95.29	88.35	82.90	92.54
Uttarakhand	Garhwal	87	92.79	87.36	96.66	91.04	85.74	94.99
Uttarakhand	Pithoragarh	88	96.13	92.91	98.35	94.84	90.63	97.63
Uttarakhand	Bageshwar	89	93.52	89.11	96.84	92.29	88.17	95.50
Uttarakhand	Almora	90	93.39	88.11	96.92	85.53	79.27	90.92
Uttarakhand	Champawat	91	93.74	89.42	96.84	90.85	85.80	94.93
Uttarakhand	Nainital	92	93.05	87.96	96.82	90.64	86.33	94.47
Uttarakhand	Udham Singh Nagar	93	93.19	88.63	96.70	90.52	85.84	94.45
Uttarakhand	Hardwar	94	83.48	75.29	90.19	87.50	82.45	91.93
Haryana	Panchkula	95	93.04	87.03	97.07	81.37	72.79	88.89
Haryana	Ambala	96	85.23	77.63	91.30	82.83	74.13	90.53
Haryana	Yamunanagar	97	87.38	81.40	92.61	87.03	79.90	92.51
Haryana	Kurukshetra	98	91.07	86.41	94.80	75.06	67.34	82.36
Haryana	Kaithal	99	89.01	82.97	94.01	75.94	68.42	82.84
Haryana	Karnal	100	90.29	85.16	94.43	81.52	72.78	88.68
Haryana	Panipat	101	88.13	81.60	93.59	88.76	81.63	94.28
Haryana	Sonipat	102	89.87	83.83	94.48	73.09	63.60	81.42
Haryana	Jind	103	87.74	81.70	92.55	81.88	74.72	88.03
Haryana	Fatehabad	104	92.20	87.20	96.03	82.32	75.66	88.04
Haryana	Sirsa	105	87.52	75.81	95.55	75.34	67.24	82.48
Haryana	Hisar	106	88.40	82.90	93.03	84.62	77.34	90.77

Haryana	Rohtak	107	85.38	77.97	91.59	68.25	58.03	77.66
Haryana	Jhajjar	108	85.15	78.53	90.84	86.07	79.68	91.28
Haryana	Mahendragarh	109	77.14	70.05	83.82	60.59	53.17	68.25
Haryana	Rewari	110	81.79	74.28	88.06	72.43	64.37	79.84
Haryana	Gurgaon	111	93.92	86.84	98.03	80.42	70.71	88.50
Haryana	Mewat	112	78.68	71.12	85.53	58.34	50.20	66.04
Haryana	Faridabad	113	79.43	67.61	88.97	77.06	62.82	88.31
Haryana	Palwal	114	78.38	71.52	84.47	75.04	67.41	82.20
Rajasthan	Ganganagar	115	91.88	87.30	95.53	76.32	68.92	82.95
Rajasthan	Hanumangarh	116	84.01	78.46	89.05	78.68	71.96	84.98
Rajasthan	Bikaner	117	84.57	78.41	89.95	83.17	77.36	88.10
Rajasthan	Churu	118	83.79	77.66	89.11	72.29	64.38	79.86
Rajasthan	Jhunjhunun	119	93.59	89.31	96.74	81.17	74.56	86.91
Rajasthan	Alwar	120	90.56	85.87	94.30	78.96	72.55	84.79
Rajasthan	Bharatpur	121	76.60	69.12	83.44	75.23	68.38	81.66
Rajasthan	Dhaulpur	122	86.70	80.94	91.79	87.04	81.50	91.41
Rajasthan	Karauli	123	81.46	75.92	86.78	87.32	81.78	92.18
Rajasthan	Sawai Madhopur	124	80.27	73.70	85.85	78.03	71.46	83.75
Rajasthan	Dausa	125	80.63	74.37	85.90	77.26	70.61	83.38
Rajasthan	Jaipur	126	85.88	79.92	90.97	82.90	77.54	87.70
Rajasthan	Sikar	127	88.11	82.76	92.52	83.56	77.03	89.12
Rajasthan	Nagaur	128	86.24	80.80	90.97	84.12	77.79	89.25
Rajasthan	Jodhpur	129	91.13	86.35	94.87	82.48	76.78	87.46
Rajasthan	Jaisalmer	130	90.68	86.03	94.32	76.27	69.62	82.45
Rajasthan	Barmer	131	91.59	87.52	94.89	71.61	63.92	78.92
Rajasthan	Jalor	132	88.74	83.85	92.80	77.25	69.99	83.61
Rajasthan	Sirohi	133	85.17	79.78	89.93	87.67	82.13	92.38
Rajasthan	Pali	134	83.63	77.23	89.09	83.88	76.88	89.79
Rajasthan	Ajmer	135	82.00	73.71	88.73	87.31	82.22	91.35
Rajasthan	Tonk	136	84.77	78.64	90.13	80.59	73.82	86.80
Rajasthan	Bundi	137	86.79	81.52	91.57	74.08	66.41	81.29
Rajasthan	Bhilwara	138	94.88	91.06	97.57	88.51	82.47	93.46
Rajasthan	Rajsamand	139	88.23	82.47	93.03	81.29	74.44	87.18
Rajasthan	Dungarpur	140	87.75	83.16	91.73	85.81	80.34	90.56
Rajasthan	Banswara	141	89.81	85.04	93.76	81.19	74.98	86.65
Rajasthan	Chittaurgarh	142	86.00	79.80	91.25	76.84	69.64	83.48

Rajasthan	Kota	143	83.19	74.47	90.54	84.01	78.59	88.57
Rajasthan	Baran	144	87.65	82.56	92.19	85.21	79.48	90.40
Rajasthan	Jhalawar	145	83.70	77.71	89.43	82.26	75.23	88.08
Rajasthan	Udaipur	146	88.65	83.23	93.21	81.50	74.76	87.40
Rajasthan	Pratapgarh	147	90.33	85.38	94.22	78.54	72.02	84.20
Uttar Pradesh	Saharanpur	148	94.56	90.22	97.58	81.27	75.39	86.47
Uttar Pradesh	Bijnor	149	87.72	82.18	92.52	78.18	70.97	84.49
Uttar Pradesh	Rampur	150	91.69	86.09	95.91	86.26	80.41	91.06
Uttar Pradesh	Jyotiba Phule Nagar	151	92.09	87.59	95.58	77.28	70.07	84.27
Uttar Pradesh	Meerut	152	91.40	85.67	95.86	82.65	77.26	87.11
Uttar Pradesh	Baghpat	153	90.64	85.64	94.65	80.28	73.38	86.27
Uttar Pradesh	Gautam Buddha Nagar	154	81.30	71.92	89.27	82.88	77.56	87.68
Uttar Pradesh	Bulandshahr	155	90.69	85.19	94.97	79.99	72.90	86.22
Uttar Pradesh	Aligarh	156	88.63	82.99	93.29	81.76	76.29	86.88
Uttar Pradesh	Mahamaya Nagar	157	79.96	72.71	86.14	78.00	71.35	84.31
Uttar Pradesh	Mathura	158	84.00	77.82	89.38	81.97	74.80	87.88
Uttar Pradesh	Agra	159	80.53	72.59	87.62	90.35	86.25	93.83
Uttar Pradesh	Firozabad	160	87.65	82.04	92.34	82.22	76.52	87.40
Uttar Pradesh	Mainpuri	161	90.21	85.19	94.47	80.18	73.38	86.32
Uttar Pradesh	Bareilly	162	86.63	79.48	92.32	84.97	79.42	89.62
Uttar Pradesh	Pilibhit	163	91.40	86.61	95.26	82.56	75.94	88.44
Uttar Pradesh	Shahjahanpur	164	87.49	81.80	92.18	84.10	77.79	89.53
Uttar Pradesh	Sitapur	165	84.58	78.36	89.90	84.41	78.29	89.87
Uttar Pradesh	Hardoi	166	84.02	78.08	89.19	67.71	60.02	75.17
Uttar Pradesh	Unnao	167	82.88	76.51	88.68	88.88	83.18	93.45
Uttar Pradesh	Lucknow	168	95.39	90.71	98.42	83.79	77.73	89.04
Uttar Pradesh	Farrukhabad	169	86.32	80.65	91.18	80.64	73.60	86.66
Uttar Pradesh	Kannauj	170	84.74	78.59	90.17	80.99	74.12	87.24
Uttar Pradesh	Etawah	171	84.77	78.72	90.07	87.64	81.59	92.59
Uttar Pradesh	Auraiya	172	86.87	81.33	91.77	77.46	70.83	83.81
Uttar Pradesh	Kanpur Dehat	173	80.51	74.25	86.31	88.55	83.20	93.19
Uttar Pradesh	Kanpur Nagar	174	84.58	76.74	91.12	85.13	79.51	90.00
Uttar Pradesh	Jalaun	175	83.00	76.91	88.44	71.96	63.81	79.72
Uttar Pradesh	Jhansi	176	90.88	85.46	94.98	85.92	79.97	90.64
Uttar Pradesh	Lalitpur	177	82.76	77.35	87.42	89.81	84.25	94.18
Uttar Pradesh	Hamirpur	178	89.47	84.29	93.80	78.96	70.82	86.01

Uttar Pradesh	Mahoba	179	82.68	76.32	88.09	84.27	76.88	90.46
Uttar Pradesh	Banda	180	81.30	74.21	87.35	87.35	81.03	92.26
Uttar Pradesh	Chitrakoot	181	84.07	77.64	89.42	85.82	79.73	90.75
Uttar Pradesh	Fatehpur	182	87.58	81.87	92.37	86.34	79.67	91.74
Uttar Pradesh	Pratapgarh	183	92.92	88.87	96.19	77.45	70.50	83.69
Uttar Pradesh	Kaushambi	184	90.35	84.60	94.79	82.01	75.30	87.67
Uttar Pradesh	Allahabad	185	85.38	78.64	91.05	84.24	77.47	89.95
Uttar Pradesh	Bara Banki	186	75.85	68.51	82.73	82.69	75.77	88.74
Uttar Pradesh	Faizabad	187	88.09	83.30	92.44	76.74	69.75	83.06
Uttar Pradesh	Ambedkar Nagar	188	91.49	86.66	95.29	80.07	73.98	85.30
Uttar Pradesh	Bahraich	189	74.31	67.08	80.98	74.52	67.26	81.35
Uttar Pradesh	Shrawasti	190	87.33	82.42	91.51	76.81	70.12	83.16
Chhattisgarh	Balrampur	191	92.74	87.82	96.22	92.24	81.81	97.95
Uttar Pradesh	Gonda	192	90.87	86.25	94.66	80.10	73.68	86.05
Uttar Pradesh	Siddharthnagar	193	89.02	84.47	92.99	71.62	64.67	78.25
Uttar Pradesh	Basti	194	93.38	89.52	96.38	84.52	78.13	89.73
Uttar Pradesh	Sant Kabir Nagar	195	91.34	86.98	94.90	81.96	75.38	87.54
Uttar Pradesh	Maharajganj	196	90.67	86.39	94.12	86.20	81.18	90.68
Uttar Pradesh	Gorakhpur	197	85.05	79.34	90.20	82.69	76.68	88.15
Uttar Pradesh	Kushinagar	198	92.18	87.88	95.63	78.61	72.35	84.10
Uttar Pradesh	Deoria	199	82.68	76.14	88.37	83.12	77.25	88.20
Uttar Pradesh	Azamgarh	200	89.18	84.08	93.74	79.59	72.90	85.36
Uttar Pradesh	Mau	201	86.85	80.69	91.85	76.18	68.86	83.01
Uttar Pradesh	Ballia	202	90.93	85.47	95.16	80.57	74.36	86.30
Uttar Pradesh	Jaunpur	203	85.13	80.11	89.75	73.86	67.41	79.85
Uttar Pradesh	Ghazipur	204	88.99	84.33	93.00	77.81	71.18	83.69
Uttar Pradesh	Chandauli	205	89.34	84.30	93.45	83.90	78.54	88.55
Uttar Pradesh	Varanasi	206	82.28	75.71	88.58	81.19	75.74	86.07
Uttar Pradesh	Sant Ravidas Nagar	207	90.46	85.86	94.55	74.52	67.83	80.58
Uttar Pradesh	Mirzapur	208	87.07	81.71	91.81	78.56	72.36	84.40
Uttar Pradesh	Sonbhadra	209	88.14	82.19	92.86	82.20	75.73	87.97
Uttar Pradesh	Etah	210	83.20	77.26	88.30	89.28	83.88	93.48
Uttar Pradesh	Kanshiram Nagar	211	83.91	77.63	89.31	73.07	65.27	80.22
Bihar	Pashchim Champaran	212	85.73	79.60	90.94	70.80	63.39	77.62
Bihar	Purba Champaran	213	90.52	85.74	94.29	73.14	66.14	79.51
Bihar	Sheohar	214	83.64	77.05	89.18	73.42	66.72	79.65

Bihar	Sitamarhi	215	88.51	83.37	92.80	84.17	78.50	88.98
Bihar	Madhubani	216	90.72	85.42	94.98	79.22	73.06	84.61
Bihar	Supaul	217	93.16	89.01	96.32	72.58	65.86	78.61
Bihar	Araria	218	91.34	86.83	94.74	86.68	81.37	91.36
Bihar	Kishanganj	219	89.38	83.69	93.98	71.63	64.55	78.10
Bihar	Purnia	220	88.19	82.90	92.54	71.63	64.45	78.06
Bihar	Katihar	221	85.04	78.45	90.62	63.78	56.06	71.27
Bihar	Madhepura	222	90.64	86.12	94.36	66.63	60.40	72.76
Bihar	Saharsa	223	84.58	78.68	89.75	74.68	68.06	80.93
Bihar	Darbhanga	224	86.57	80.90	91.55	84.41	78.70	89.44
Bihar	Muzaffarpur	225	90.04	85.04	94.10	64.71	56.93	72.48
Bihar	Gopalganj	226	90.14	85.05	94.33	82.21	76.25	87.56
Bihar	Siwan	227	87.21	81.54	92.02	61.23	54.52	67.69
Bihar	Saran	228	81.68	75.09	87.58	68.25	61.66	74.96
Bihar	Vaishali	229	89.24	84.05	93.65	62.73	55.75	69.39
Bihar	Samastipur	230	86.64	80.63	91.50	77.89	71.16	83.95
Bihar	Begusarai	231	82.94	76.15	88.81	84.09	78.47	89.20
Bihar	Khagaria	232	85.23	79.54	90.22	82.91	77.61	87.69
Bihar	Bhagalpur	233	85.64	79.82	90.74	72.49	65.44	78.78
Bihar	Banka	234	86.05	80.39	90.67	79.83	74.34	84.87
Bihar	Munger	235	79.46	72.22	85.82	72.11	63.94	79.82
Bihar	Lakhisarai	236	89.37	84.03	93.53	70.46	63.02	77.26
Bihar	Sheikhpura	237	80.90	74.26	86.96	84.50	78.56	89.69
Bihar	Nalanda	238	83.79	77.47	89.28	63.84	56.19	70.99
Bihar	Patna	239	80.08	71.67	87.34	81.23	75.68	86.11
Bihar	Bhojpur	240	84.41	78.70	89.51	79.52	73.68	84.86
Bihar	Buxer	241	79.71	72.80	86.03	78.98	72.95	84.62
Bihar	Kaimur (Bhabua)	242	82.27	75.75	87.94	73.91	67.19	80.19
Bihar	Rohtas	243	81.11	74.59	87.10	81.45	75.80	86.41
Bihar	Gaya	244	93.06	88.74	96.43	77.19	71.44	82.66
Bihar	Nawada	245	83.01	76.48	88.68	85.22	79.60	90.25
Bihar	Jamui	246	83.62	77.74	88.89	84.37	78.97	89.25
Bihar	Jehanabad	247	76.60	69.42	83.06	82.44	76.02	88.02
Bihar	Arwal	248	81.86	75.38	87.50	73.38	66.42	79.69
Sikkim	North District	249	92.78	87.65	96.62	90.33	85.53	94.01
Sikkim	West District	250	94.03	89.13	97.36	94.95	91.75	97.37

Sikkim	South District	251	92.43	86.65	96.49	NA	NA	NA
Sikkim	East District	252	89.48	81.65	95.26	94.51	91.47	96.94
Arunachal Pradesh	Tawang	253	82.57	75.58	88.70	92.39	86.55	96.52
Arunachal Pradesh	West Kameng	254	79.87	72.32	86.12	83.28	76.60	89.21
Arunachal Pradesh	East Kameng	255	82.90	75.70	88.78	83.15	76.11	88.91
Arunachal Pradesh	Papum Pare	256	90.32	83.49	95.44	76.76	70.43	82.77
Arunachal Pradesh	Upper Subansiri	257	94.76	90.83	97.55	88.71	83.01	93.44
Arunachal Pradesh	Upper Siang	258	90.35	83.79	94.96	84.74	77.63	90.35
Arunachal Pradesh	Changlang	259	89.66	84.47	93.78	94.20	90.46	97.00
Arunachal Pradesh	Lower Subansiri	260	86.40	80.61	91.25	90.58	85.17	94.70
Arunachal Pradesh	Dibang Valley	261	89.40	82.96	94.45	89.52	83.11	94.34
Arunachal Pradesh	Lower Dibang Valley	262	89.56	84.02	94.11	87.25	81.28	91.95
Arunachal Pradesh	Anjaw	263	91.76	86.40	95.93	84.27	77.84	89.82
Nagaland	Mon	264	98.79	97.07	99.69	96.34	92.68	98.59
Nagaland	Mokokchung	265	98.99	97.31	99.78	97.54	94.22	99.25
Nagaland	Zunheboto	266	99.01	97.33	99.78	98.08	95.90	99.37
Nagaland	Wokha	267	99.04	97.49	99.76	96.92	93.35	98.91
Nagaland	Dimapur	268	98.92	96.80	99.79	96.97	94.12	98.73
Nagaland	Phek	269	99.04	97.55	99.77	97.78	95.32	99.20
Nagaland	Tuensang	270	99.04	97.42	99.77	97.62	94.73	99.20
Nagaland	Longleng	271	98.75	96.80	99.68	98.17	95.97	99.37
Nagaland	Kiphire	272	99.07	97.63	99.77	97.72	95.13	99.19
Nagaland	Kohima	273	98.64	96.23	99.72	96.71	93.47	98.80
Nagaland	Peren	274	99.10	97.69	99.78	97.36	94.74	99.03
Manipur	Senapati	275	95.68	91.59	98.29	93.42	89.69	96.30
Manipur	Tamenglong	276	92.58	87.13	96.43	97.22	94.75	98.80
Manipur	Churachandpur	277	90.84	85.25	95.20	96.98	94.14	98.73
Manipur	Bishnupur	278	93.29	88.29	96.97	96.78	94.26	98.55
Manipur	Thoubal	279	95.29	91.01	98.18	95.42	92.44	97.71
Manipur	Imphal West	280	95.71	90.57	98.69	96.31	93.32	98.24
Manipur	Imphal East	281	92.63	87.42	96.58	96.74	94.31	98.48
Manipur	Ukhrul	282	96.34	92.32	98.71	97.04	93.89	98.95
Manipur	Chandel	283	94.99	90.59	97.98	97.10	94.37	98.83
Mizoram	Mamit	284	98.45	96.46	99.55	97.66	95.36	99.08
Mizoram	Kolasib	285	97.92	94.71	99.50	97.51	95.07	99.02
Mizoram	Aizawl	286	98.33	94.45	99.75	95.85	89.56	99.03

Mizoram	Champhai	287	96.53	92.77	98.79	95.57	92.46	97.79
Mizoram	Serchhip	288	98.15	95.03	99.58	97.15	94.64	98.84
Mizoram	Lunglei	289	98.40	95.89	99.62	95.94	92.58	98.24
Mizoram	Lawngtlai	290	97.76	95.07	99.27	94.57	90.03	97.60
Tripura	Dhalai	291	82.33	75.89	87.80	92.10	87.85	95.44
Meghalaya	South Garo Hills	292	90.13	85.14	94.18	95.77	92.51	98.10
Meghalaya	Ribhoi	293	94.33	90.45	97.22	93.52	89.17	96.65
Meghalaya	East Khasi Hills	294	95.74	91.08	98.49	94.78	91.40	97.37
Assam	Kokrajhar	295	96.44	93.29	98.49	83.98	77.65	89.19
Assam	Goalpara	296	95.95	92.79	98.13	84.28	79.20	88.86
Assam	Barpeta	297	96.51	93.65	98.49	82.56	76.52	87.85
Assam	Morigaon	298	92.29	87.97	95.59	89.47	84.62	93.48
Assam	Lakhimpur	299	95.55	92.03	97.99	93.94	90.18	96.79
Assam	Dhemaji	300	94.90	91.35	97.51	92.90	88.81	95.99
Assam	Tinsukia	301	88.37	82.55	93.01	93.31	89.15	96.50
Assam	Dibrugarh	302	93.75	89.50	96.81	92.72	87.92	96.16
Assam	Golaghat	303	95.02	91.29	97.66	96.07	93.29	98.14
Assam	Dima Hasao	304	93.98	89.83	97.21	85.71	79.26	91.14
Assam	Cachar	305	79.73	73.58	85.38	86.06	80.17	91.25
Assam	Karimganj	306	85.94	80.22	91.00	83.82	77.72	88.92
Assam	Hailakandi	307	80.25	73.94	85.97	77.72	70.41	84.18
Assam	Bongaigaon	308	94.96	91.42	97.59	70.57	63.30	77.43
Assam	Chirang	309	88.83	83.90	93.05	75.22	68.72	80.99
Assam	Kamrup	310	96.04	92.63	98.31	78.70	71.91	84.49
Assam	Kamrup Metropolitan	311	93.14	83.94	98.37	92.80	83.79	97.72
Assam	Nalbari	312	91.19	86.75	94.86	69.20	62.42	75.72
Assam	Baksa	313	91.47	86.88	95.03	85.55	80.32	90.17
Assam	Darrang	314	95.74	92.56	97.97	82.03	75.80	87.53
Assam	Udalguri	315	91.23	86.38	95.03	74.60	68.04	80.47
West Bengal	Darjiling	316	95.84	91.86	98.32	85.16	76.67	91.99
West Bengal	Jalpaiguri	317	92.91	87.96	96.40	89.74	83.82	94.11
West Bengal	Koch Bihar	318	91.89	87.46	95.59	93.07	88.61	96.42
West Bengal	Uttar Dinajpur	319	89.27	83.83	93.61	90.72	85.56	94.65
West Bengal	Dakshin Dinajpur	320	91.94	87.26	95.50	91.30	86.14	95.24
West Bengal	Maldah	321	87.12	81.74	91.71	88.50	83.17	93.06
West Bengal	Murshidabad	322	90.70	86.14	94.65	90.09	85.35	93.95

West Bengal	Birbhum	323	87.39	81.99	92.07	87.77	82.08	92.46
West Bengal	Nadia	324	86.56	80.21	91.90	89.65	84.04	94.03
West Bengal	North Twenty Four Parganas	325	92.48	85.91	96.72	84.68	76.47	91.44
West Bengal	Hugli	326	86.95	81.08	91.91	92.57	87.47	96.50
West Bengal	Bankura	327	87.42	81.68	92.29	88.60	83.46	92.85
West Bengal	Puruliya	328	89.59	84.12	93.95	87.90	82.02	92.62
West Bengal	South Twenty Four Parganas	329	76.50	69.31	82.96	92.52	87.70	96.29
West Bengal	Paschim Medinipur	330	89.98	84.41	94.14	86.40	80.68	91.34
West Bengal	Purba Medinipur	331	81.79	74.97	87.68	90.05	85.33	93.94
Jharkhand	Garhwa	332	96.03	92.87	98.20	92.24	87.64	95.70
Jharkhand	Chatra	333	88.78	83.89	92.98	83.05	76.61	88.50
Jharkhand	Kodarma	334	87.20	81.85	92.08	87.27	81.91	92.05
Jharkhand	Giridih	335	90.89	86.16	94.62	78.99	72.86	84.20
Jharkhand	Deoghar	336	91.88	87.54	95.34	87.63	82.25	92.12
Jharkhand	Godda	337	86.70	80.22	92.01	90.19	85.09	94.17
Jharkhand	Sahibganj	338	91.74	87.14	95.48	87.79	83.05	92.16
Jharkhand	Pakur	339	93.86	89.44	96.98	85.78	80.28	90.76
Jharkhand	Dhanbad	340	90.63	83.62	95.99	77.81	71.95	83.04
Jharkhand	Bokaro	341	91.02	84.81	95.56	82.71	77.41	87.75
Jharkhand	Lohardaga	342	96.63	93.53	98.68	89.38	83.71	93.91
Jharkhand	Purbi Singhbhum	343	95.67	91.33	98.41	89.22	84.52	93.39
Jharkhand	Palamu	344	89.01	84.19	93.11	85.47	78.81	90.89
Jharkhand	Latehar	345	85.35	80.04	90.13	88.69	83.04	92.85
Jharkhand	Hazaribagh	346	89.23	84.39	93.36	83.46	77.81	88.49
Jharkhand	Ramgarh	347	90.94	84.89	95.36	92.38	88.17	95.65
Jharkhand	Dumka	348	93.32	88.71	96.65	92.01	87.20	95.75
Jharkhand	Jamtara	349	87.49	82.46	92.11	92.01	87.52	95.50
Jharkhand	Ranchi	350	93.14	88.04	97.04	92.28	87.99	95.54
Jharkhand	Khunti	351	84.42	78.23	89.72	86.67	80.72	91.58
Jharkhand	Gumla	352	81.86	75.85	87.31	89.76	84.82	94.01
Jharkhand	Simdega	353	94.32	90.36	97.35	94.53	90.72	97.27
Jharkhand	Pashchimi Singhbhum	354	84.64	77.95	90.08	91.94	86.96	95.74
Jharkhand	Saraikela-Kharsawan	355	80.23	73.91	86.33	93.22	88.22	96.69
Odisha	Bargarh	356	91.43	86.39	95.39	81.03	74.22	87.23
Odisha	Jharsuguda	357	93.89	88.58	97.44	83.44	77.21	88.74
Odisha	Sambalpur	358	94.98	90.62	97.88	87.57	81.35	92.76

Odisha	Debagarh	359	90.62	84.80	94.97	82.61	76.34	88.10
Odisha	Sundargarh	360	95.41	90.76	98.24	86.76	81.54	91.04
Odisha	Kendujhar	361	87.74	81.72	92.49	77.57	70.43	84.16
Odisha	Mayurbhanj	362	91.53	86.60	95.38	82.82	75.82	88.45
Odisha	Baleshwar	363	88.69	82.67	93.42	77.78	70.94	84.14
Odisha	Bhadrak	364	86.70	80.31	91.87	81.02	73.47	87.36
Odisha	Kendrapara	365	87.27	81.24	92.15	75.97	67.98	83.23
Odisha	Cuttack	366	86.24	79.07	92.20	78.97	71.14	86.12
Odisha	Jajapur	367	82.02	75.39	88.01	83.36	76.99	88.60
Odisha	Dhenkanal	368	90.83	85.32	94.93	76.84	69.77	83.22
Odisha	Anugul	369	92.85	88.27	96.09	85.24	79.61	90.41
Odisha	Nayagarh	370	90.45	85.57	94.29	82.53	76.38	87.91
Odisha	Khordha	371	92.52	87.35	96.35	82.13	75.54	87.59
Odisha	Puri	372	90.84	85.39	94.94	85.49	79.69	90.60
Odisha	Ganjam	373	91.93	86.58	95.96	76.85	69.09	84.03
Odisha	Gajapati	374	96.44	92.99	98.56	86.65	80.64	91.75
Odisha	Kandhamal	375	91.59	86.36	95.52	89.57	84.59	93.71
Odisha	Baudh	376	93.45	89.08	96.60	82.08	75.53	87.82
Odisha	Subarnapur	377	93.53	89.03	96.79	84.61	78.68	89.95
Odisha	Balangir	378	94.08	89.79	97.07	86.43	80.61	91.29
Odisha	Nuapada	379	95.78	92.28	98.21	85.02	79.14	90.17
Odisha	Kalahandi	380	88.98	83.58	93.55	77.57	70.00	84.39
Odisha	Rayagada	381	90.08	84.69	94.42	82.27	75.33	88.47
Odisha	Nabarangapur	382	93.73	89.69	96.94	85.68	80.11	90.42
Odisha	Koraput	383	94.53	90.83	97.32	83.35	76.67	88.98
Odisha	Malkangiri	384	90.40	85.44	94.50	82.84	76.62	88.34
Chhattisgarh	Koriya	385	94.95	90.69	97.84	89.51	85.08	93.34
Chhattisgarh	Jashpur	386	96.73	93.64	98.65	90.19	85.57	94.10
Chhattisgarh	Raigarh	387	92.06	87.33	95.93	89.78	84.80	94.00
Chhattisgarh	Korba	388	93.60	88.56	97.14	90.63	85.99	94.26
Chhattisgarh	Janjgir-Champa	389	92.39	87.54	96.09	91.13	86.40	94.96
Chhattisgarh	Kabeerdham	390	92.42	87.12	96.38	86.61	80.86	91.54
Chhattisgarh	Rajnandgaon	391	91.32	86.75	95.16	92.80	88.84	95.90
Chhattisgarh	Mahasamund	392	89.30	83.98	93.59	91.16	86.58	94.76
Chhattisgarh	Dhamtari	393	91.36	86.36	95.49	90.87	86.43	94.66
Chhattisgarh	Uttar Bastar Kanker	394	87.47	80.74	92.49	89.35	83.82	93.88

Chhattisgarh	Narayanpur	395	92.64	88.14	96.13	84.65	78.91	89.74
Chhattisgarh	Bijapur	396	93.03	88.44	96.45	91.60	87.25	94.98
Madhya Pradesh	Sheopur	397	84.92	78.53	90.26	85.15	79.18	90.18
Madhya Pradesh	Morena	398	88.68	83.46	93.20	81.32	74.94	86.93
Madhya Pradesh	Bhind	399	81.14	73.49	87.65	85.30	78.50	90.81
Madhya Pradesh	Gwalior	400	86.52	78.55	92.71	80.20	74.49	85.31
Madhya Pradesh	Datia	401	87.80	81.47	93.02	87.98	82.39	92.60
Madhya Pradesh	Shivpuri	402	84.98	77.98	91.26	81.81	75.90	86.95
Madhya Pradesh	Tikamgarh	403	89.75	83.56	94.65	82.44	75.80	88.03
Madhya Pradesh	Chhatarpur	404	86.66	80.40	91.71	79.29	72.08	85.91
Madhya Pradesh	Panna	405	81.51	75.14	86.99	81.07	74.72	86.68
Madhya Pradesh	Sagar	406	80.28	73.34	86.28	85.87	78.51	91.70
Madhya Pradesh	Damoh	407	83.51	76.47	89.43	76.53	69.21	83.22
Madhya Pradesh	Satna	408	89.57	82.30	95.01	74.61	67.65	80.70
Madhya Pradesh	Rewa	409	87.95	80.28	93.87	85.64	80.38	90.48
Madhya Pradesh	Umaria	410	87.60	81.58	92.48	77.68	71.14	83.63
Madhya Pradesh	Neemuch	411	85.32	78.91	90.69	82.13	75.48	87.95
Madhya Pradesh	Mandsaur	412	93.94	89.66	97.14	78.94	72.67	84.50
Madhya Pradesh	Ratlam	413	78.81	72.35	84.61	84.80	78.17	90.48
Madhya Pradesh	Ujjain	414	76.14	67.84	83.93	74.68	68.14	80.60
Madhya Pradesh	Dewas	415	82.07	75.81	88.00	73.96	66.76	80.47
Madhya Pradesh	Dhar	416	84.48	77.21	90.84	83.91	78.41	88.86
Madhya Pradesh	Indore	417	76.08	65.73	84.84	83.37	74.78	90.49
Madhya Pradesh	Khargone (West Nimar)	418	83.55	77.02	89.15	82.45	76.86	87.59
Madhya Pradesh	Barwani	419	73.54	66.53	80.06	84.84	79.50	89.80
Madhya Pradesh	Rajgarh	420	70.36	62.38	77.62	78.34	70.75	85.06
Madhya Pradesh	Vidisha	421	82.38	75.88	87.99	66.86	58.79	74.58
Madhya Pradesh	Bhopal	422	80.33	65.08	91.90	69.45	55.15	82.72
Madhya Pradesh	Sehore	423	70.13	61.79	77.92	82.92	76.43	88.56
Madhya Pradesh	Raisen	424	89.87	80.76	96.28	81.18	74.53	87.19
Madhya Pradesh	Betul	425	93.31	88.85	96.63	80.46	73.19	86.90
Madhya Pradesh	Harda	426	90.07	84.80	94.16	85.15	79.70	89.83
Madhya Pradesh	Hoshangabad	427	85.41	78.63	91.19	92.30	88.31	95.40
Madhya Pradesh	Katni	428	89.94	82.82	95.04	85.03	78.79	90.26
Madhya Pradesh	Jabalpur	429	89.16	78.28	96.37	90.50	86.04	94.18
Madhya Pradesh	Narsimhapur	430	91.68	86.69	95.42	87.69	82.13	92.49

Madhya Pradesh	Dindori	431	91.42	86.68	95.00	88.21	82.77	92.64
Madhya Pradesh	Mandla	432	91.40	86.81	95.02	87.65	82.76	91.98
Madhya Pradesh	Chhindwara	433	86.58	79.47	92.15	87.93	82.15	92.55
Madhya Pradesh	Seoni	434	93.62	89.46	96.92	81.41	74.85	86.97
Madhya Pradesh	Balaghat	435	89.42	83.28	94.28	88.38	83.04	92.92
Madhya Pradesh	Guna	436	79.39	72.24	85.28	83.82	77.76	89.10
Madhya Pradesh	Ashoknagar	437	89.12	82.67	94.03	82.26	75.94	88.23
Madhya Pradesh	Shahdol	438	94.26	89.69	97.52	81.04	74.51	86.85
Madhya Pradesh	Anuppur	439	94.38	89.76	97.54	78.05	70.97	84.61
Madhya Pradesh	Singrauli	440	90.46	83.32	95.61	81.26	74.96	86.80
Madhya Pradesh	Jhabua	441	84.81	78.45	89.80	83.88	78.17	88.90
Madhya Pradesh	Alirajpur	442	86.61	81.14	91.22	83.15	77.48	87.95
Madhya Pradesh	Khandwa (East Nimar)	443	92.48	79.44	98.56	87.41	82.01	91.95
Madhya Pradesh	Burhanpur	444	84.74	77.78	90.36	83.20	77.74	88.06
Gujarat	Kachchh	445	93.63	89.03	96.94	83.21	77.03	88.62
Gujarat	Banas Kantha	446	85.90	79.82	91.23	88.50	83.76	92.35
Gujarat	Patan	447	94.22	89.83	97.24	74.77	68.93	80.17
Gujarat	Mahesana	448	92.46	87.00	96.48	90.60	86.23	94.08
Gujarat	Gandhinagar	449	88.11	81.39	93.56	86.86	81.25	91.68
Gujarat	Porbandar	450	95.04	90.30	98.00	78.83	71.12	85.23
Gujarat	Anand	451	89.52	83.36	94.42	91.80	87.34	95.27
Gujarat	Dohad	452	84.63	78.82	89.59	80.36	74.61	85.44
Gujarat	Narmada	453	94.77	90.96	97.54	91.04	86.96	94.32
Gujarat	Bharuch	454	87.84	80.60	93.65	90.47	85.42	94.65
Gujarat	The Dangs	455	92.17	86.98	96.02	93.41	89.84	96.25
Gujarat	Navsari	456	91.78	87.10	95.77	92.30	87.58	95.95
Gujarat	Valsad	457	89.68	83.81	94.44	94.44	90.58	97.19
Gujarat	Tapi	458	94.37	90.61	97.21	89.59	85.39	93.29
Dadra & Nagar Haveli And Daman & Diu	Daman	459	97.95	93.28	99.74	89.13	79.93	95.32
Dadra & Nagar Haveli And Daman & Diu	Dadra & Nagar Haveli	460	92.05	85.65	96.57	75.95	67.05	83.83
Maharashtra	Nandurbar	461	88.17	82.40	93.27	77.95	70.27	84.67
Maharashtra	Dhule	462	83.03	75.97	89.41	87.93	81.48	92.75
Maharashtra	Jalgaon	463	86.30	79.47	91.97	89.20	81.91	94.56
Maharashtra	Buldana	464	87.30	81.01	92.57	91.30	86.16	95.26
Maharashtra	Akola	465	91.14	86.04	95.17	89.64	82.63	94.81
Maharashtra	Washim	466	88.42	83.26	92.71	89.45	83.97	93.99

Maharashtra	Amravati	467	91.24	85.53	95.62	89.66	82.53	94.94
Maharashtra	Wardha	468	93.03	88.11	96.62	93.58	88.03	97.30
Maharashtra	Nagpur	469	92.79	85.48	97.44	94.00	88.24	97.60
Maharashtra	Bhandara	470	86.08	79.69	91.43	92.61	87.74	96.23
Maharashtra	Gondiya	471	92.82	88.30	96.24	89.42	83.70	93.96
Maharashtra	Gadchiroli	472	80.70	73.90	86.74	90.20	84.66	94.59
Maharashtra	Chandrapur	473	85.72	78.29	91.74	86.88	79.40	92.66
Maharashtra	Yavatmal	474	86.37	80.17	91.77	93.80	89.40	97.04
Maharashtra	Nanded	475	79.43	71.96	86.32	85.63	80.16	90.24
Maharashtra	Hingoli	476	85.09	79.61	90.01	83.37	76.96	88.97
Maharashtra	Parbhani	477	82.96	75.68	89.19	89.84	83.44	94.62
Maharashtra	Jalna	478	80.46	73.28	86.99	70.12	61.77	77.55
Maharashtra	Nashik	479	87.74	80.50	93.49	90.64	84.73	95.35
Maharashtra	Raigarh	482	94.55	89.80	97.75	95.50	90.87	98.19
Maharashtra	Pune	483	94.86	89.68	98.01	93.39	88.35	96.95
Maharashtra	Ahmadnagar	484	89.13	83.11	93.89	90.55	84.83	95.07
Maharashtra	Bid	485	81.20	73.64	87.66	89.00	82.71	93.82
Maharashtra	Latur	486	87.89	80.97	93.28	87.56	82.04	92.21
Maharashtra	Osmanabad	487	90.39	85.14	94.66	87.36	81.16	92.46
Maharashtra	Solapur	488	88.10	80.76	93.89	88.60	81.37	93.97
Maharashtra	Satara	489	94.50	90.71	97.36	91.30	86.01	95.33
Maharashtra	Ratnagiri	490	93.68	88.84	97.04	91.94	86.31	96.07
Maharashtra	Sindhudurg	491	93.09	87.85	96.89	90.65	84.26	95.61
Maharashtra	Kolhapur	492	96.17	92.43	98.42	86.72	79.09	92.60
Maharashtra	Sangli	493	83.91	78.05	88.81	85.77	78.82	91.29
Karnataka	Belgaum	494	81.88	75.21	87.90	89.67	84.05	94.36
Karnataka	Bagalkot	495	82.49	75.63	88.34	59.83	49.54	69.58
Karnataka	Bijapur	496	85.50	78.83	91.14	76.65	69.12	83.26
Karnataka	Bidar	497	80.54	73.37	86.63	84.36	77.74	90.24
Karnataka	Raichur	498	74.35	66.68	81.43	76.75	68.26	83.97
Karnataka	Koppal	499	85.66	79.30	90.83	83.36	76.14	89.12
Karnataka	Gadag	500	70.38	61.88	77.93	70.44	60.45	79.85
Karnataka	Dharwad	501	88.56	81.28	94.24	81.65	72.89	89.02
Karnataka	Uttara Kannada	502	82.55	75.00	89.01	75.57	66.88	82.85
Karnataka	Haveri	503	78.38	70.96	85.10	81.97	74.52	88.60
Karnataka	Bellary	504	69.32	60.89	77.46	52.49	42.38	62.43

Karnataka	Chitradurga	505	84.54	77.57	90.32	78.48	70.38	85.42
Karnataka	Davanagere	506	74.23	65.23	82.41	79.37	70.44	87.04
Karnataka	Shimoga	507	82.96	76.03	88.84	80.96	71.13	88.89
Karnataka	Chikmagalur	508	78.88	70.77	85.97	83.89	77.04	89.80
Karnataka	Tumkur	509	87.08	80.48	92.74	85.81	79.22	91.38
Karnataka	Bangalore	510	88.00	67.80	98.09	67.75	41.41	88.48
Karnataka	Mandya	511	86.41	80.17	91.66	78.28	70.34	85.63
Karnataka	Hassan	512	87.58	81.56	92.45	88.49	82.44	93.14
Karnataka	Dakshina Kannada	513	81.07	72.73	88.40	81.85	72.14	89.58
Karnataka	Kodagu	514	90.07	84.37	94.39	76.75	68.51	84.02
Karnataka	Mysore	515	70.83	61.36	79.15	83.11	73.98	90.10
Karnataka	Chamarajanagar	516	79.81	73.02	85.97	75.59	67.32	83.04
Karnataka	Gulbarga	517	80.37	72.42	87.28	62.48	51.96	72.57
Karnataka	Yadgir	518	74.71	67.50	81.37	85.71	79.02	91.01
Karnataka	Kolar	519	79.72	71.94	86.51	81.71	72.63	89.41
Karnataka	Chikkaballapura	520	86.81	80.45	91.94	77.73	69.89	84.61
Karnataka	Bangalore Rural	521	87.17	80.20	92.63	84.83	77.67	90.76
Karnataka	Ramanagara	522	76.12	67.04	84.03	83.53	76.85	89.59
Goa	North Goa	523	94.73	88.90	98.35	96.41	93.99	98.14
Goa	South Goa	524	96.26	91.57	98.94	85.64	80.56	90.21
Kerala	Wayanad	525	90.40	84.65	94.81	90.90	86.80	94.35
Kerala	Kozhikode	526	94.15	87.79	98.00	93.70	89.00	96.96
Kerala	Malappuram	527	88.55	81.79	93.85	88.45	82.74	92.96
Kerala	Palakkad	528	96.44	92.90	98.63	88.47	83.58	92.71
Kerala	Thrissur	529	95.79	89.85	98.86	92.61	87.18	96.49
Kerala	Ernakulam	530	95.93	90.16	98.92	92.96	87.36	96.78
Kerala	Idukki	531	97.27	94.35	99.02	90.70	85.54	94.71
Kerala	Kottayam	532	94.57	89.60	97.77	94.23	90.11	97.14
Kerala	Pathanamthitta	533	95.78	91.86	98.40	94.53	90.46	97.41
Kerala	Kollam	534	97.21	93.69	99.16	94.11	89.54	97.10
Kerala	Thiruvananthapuram	535	95.29	90.21	98.36	93.36	88.33	96.88
Tamil Nadu	Kancheepuram	537	91.56	83.34	96.78	88.52	82.02	93.60
Tamil Nadu	Vellore	538	96.71	93.08	98.77	87.56	80.83	92.67
Tamil Nadu	Tiruvannamalai	539	91.87	86.33	96.16	78.11	71.41	83.98
Tamil Nadu	Viluppuram	540	91.97	86.79	96.19	87.47	82.26	91.68
Tamil Nadu	Salem	541	91.59	84.71	96.53	83.58	76.67	89.52

Tamil Nadu	Namakkal	542	92.28	86.50	96.45	87.86	81.92	92.92
Tamil Nadu	Erode	543	95.60	90.91	98.47	88.74	82.52	93.80
Tamil Nadu	The Nilgiris	544	96.34	91.17	99.03	81.52	74.18	87.99
Tamil Nadu	Dindigul	545	91.21	85.78	95.33	82.01	74.88	88.29
Tamil Nadu	Karur	546	92.44	86.06	96.79	82.35	74.64	88.62
Tamil Nadu	Tiruchirappalli	547	92.24	85.56	96.76	85.23	78.10	91.33
Tamil Nadu	Perambalur	548	94.13	89.45	97.37	85.59	80.02	90.23
Tamil Nadu	Ariyalur	549	90.97	85.76	94.98	81.66	75.45	87.05
Tamil Nadu	Cuddalore	550	93.99	87.99	97.59	82.85	74.94	89.46
Tamil Nadu	Nagapattinam	551	93.25	87.92	96.84	85.47	79.59	90.61
Tamil Nadu	Thiruvarur	552	93.69	88.76	97.14	89.37	84.32	93.65
Tamil Nadu	Thanjavur	553	95.41	90.38	98.31	82.79	74.85	89.53
Tamil Nadu	Pudukkottai	554	84.51	76.95	90.70	82.76	76.48	88.31
Tamil Nadu	Sivaganga	555	91.66	85.64	95.79	82.58	74.31	89.61
Tamil Nadu	Madurai	556	95.25	89.83	98.44	81.63	73.86	88.32
Tamil Nadu	Theni	557	89.33	82.42	94.72	85.80	78.34	91.82
Tamil Nadu	Virudhunagar	558	91.84	84.99	96.54	80.45	71.54	88.23
Tamil Nadu	Thoothukkudi	559	93.18	86.94	97.27	72.62	61.85	81.65
Tamil Nadu	Tirunelveli	560	93.33	87.12	97.32	86.56	78.88	92.46
Tamil Nadu	Kanniyakumari	561	94.28	85.18	98.88	89.17	81.22	94.97
Tamil Nadu	Dharmapuri	562	94.34	89.95	97.49	73.67	67.21	79.63
Tamil Nadu	Krishnagiri	563	94.34	90.28	97.30	84.90	79.25	89.56
Tamil Nadu	Coimbatore	564	96.47	90.79	99.21	82.88	73.49	90.74
Tamil Nadu	Tiruppur	565	93.71	85.77	98.16	91.39	86.30	95.30
Puducherry	Puducherry	567	96.99	92.33	99.30	79.87	72.80	86.29
Arunachal Pradesh	East Siang	568	86.23	79.69	91.54	86.76	80.44	91.82
Arunachal Pradesh	Kra Daadi	569	80.41	72.07	87.69	88.75	78.56	95.66
Arunachal Pradesh	Kurung Kumey	570	83.43	75.03	90.72	88.13	80.73	93.65
Arunachal Pradesh	Lohit	571	89.08	83.30	93.87	92.19	75.05	98.85
Arunachal Pradesh	Longding	572	91.33	86.06	95.32	94.97	90.04	98.16
Arunachal Pradesh	Namsai	573	80.29	74.19	85.81	87.52	81.01	92.73
Arunachal Pradesh	Siang	574	90.39	85.13	94.60	92.30	81.74	97.87
Arunachal Pradesh	Tirap	575	79.94	71.63	87.24	92.18	85.64	96.58
Arunachal Pradesh	West Siang	576	88.04	81.78	93.17	88.62	82.02	93.79
Assam	Biswanath	577	92.64	88.31	96.20	83.71	73.76	91.54
Assam	Charaideo	578	91.08	86.52	94.77	94.48	86.37	98.65

Assam	Dhubri	579	93.60	89.41	96.71	88.46	81.28	93.89
Assam	Hojai	580	95.09	91.09	97.94	92.42	85.86	96.94
Assam	Jorhat	581	91.37	86.13	95.40	95.33	91.78	97.87
Assam	Karbi Anglong	582	91.58	86.59	95.53	92.78	88.47	96.09
Assam	Majuli	583	94.15	90.31	96.94	95.69	88.54	99.05
Assam	Nagaon	584	91.51	86.74	95.17	90.87	85.47	95.03
Assam	Sivasagar	585	94.58	90.91	97.29	91.59	86.40	95.49
Assam	Sonitpur	586	94.16	90.01	97.21	90.86	85.19	95.36
Assam	South Salmara Mancachar	587	94.66	91.16	97.32	86.57	79.21	92.44
Assam	West Karbi Anglong	588	91.24	86.62	95.12	95.09	85.26	99.30
Chhattisgarh	Balod	589	94.12	90.11	97.16	74.20	58.51	87.09
Chhattisgarh	Baloda Bazar	590	90.78	85.46	94.80	93.02	85.78	97.56
Uttar Pradesh	Balrampur	591	87.62	82.05	92.55	65.01	58.31	71.62
Chhattisgarh	Bastar	592	90.62	85.23	94.81	89.85	84.12	94.25
Chhattisgarh	Bemetara	593	93.26	88.58	96.74	90.84	83.67	95.96
Chhattisgarh	Bilaspur	594	87.60	81.92	92.67	88.31	82.03	93.27
Chhattisgarh	Dantewada	595	93.68	89.38	96.84	89.15	80.52	95.25
Chhattisgarh	Durg	596	89.24	82.18	94.69	91.99	87.06	95.91
Chhattisgarh	Gariyaband	597	95.13	91.50	97.75	83.70	70.00	93.58
Chhattisgarh	Kodagaon	598	94.94	91.24	97.63	90.92	83.97	96.01
Chhattisgarh	Mungeli	599	92.52	87.42	96.30	94.20	86.08	98.41
Chhattisgarh	Raipur	600	94.97	89.27	98.33	90.58	85.23	94.73
Chhattisgarh	Sukma	601	93.31	89.62	96.36	84.54	77.11	91.02
Chhattisgarh	Surguja	602	93.92	89.47	97.17	94.04	86.70	98.21
Nct Of Delhi	Central	603	NA	NA	NA	59.41	26.16	87.34
Nct Of Delhi	North	606	92.52	81.22	98.27	NA	NA	NA
Nct Of Delhi	North West	608	NA	NA	NA	83.50	55.14	97.24
Nct Of Delhi	South West	612	93.26	80.26	98.94	45.67	19.56	75.12
Gujarat	Ahmadabad	614	96.57	89.69	99.44	86.83	78.26	93.63
Gujarat	Aravali	615	94.04	90.16	97.03	90.78	84.63	95.31
Gujarat	Bhavnagar	616	93.38	87.98	97.31	71.16	62.72	79.27
Gujarat	Botad	617	95.30	91.09	98.00	78.15	60.57	91.44
Gujarat	Chhota Udaipur	618	91.55	87.22	95.14	83.17	73.02	91.07
Gujarat	Devbhumi Dwarka	619	81.97	75.01	88.17	89.23	81.02	95.28
Gujarat	Gir Somnath	620	97.09	94.30	98.83	83.81	73.76	91.89
Gujarat	Jamnagar	621	89.11	81.37	94.70	83.08	74.99	89.71

Gujarat	Junagadh	622	86.21	79.69	91.71	83.62	75.14	90.56
Gujarat	Kheda	623	86.18	79.90	91.40	79.14	73.21	84.22
Gujarat	Mahisagar	624	86.53	80.80	91.36	78.53	69.14	86.58
Gujarat	Morbi	625	88.71	82.79	93.47	77.03	63.74	87.68
Gujarat	Panch Mahals	626	94.68	91.27	97.36	80.21	73.77	86.13
Gujarat	Rajkot	627	96.76	92.59	99.11	85.54	78.86	91.13
Gujarat	Sabar Kantha	628	95.17	91.09	97.92	90.26	85.40	94.56
Gujarat	Surendranagar	629	85.02	78.14	90.87	81.02	74.23	87.03
Gujarat	Vadodara	630	82.57	72.45	90.56	92.86	87.09	96.65
Haryana	Bhiwani	631	89.30	83.23	93.98	71.17	62.45	79.39
Haryana	Charkhi Dadri	632	90.39	84.82	94.65	81.01	69.00	90.09
Madhya Pradesh	Agar Malwa	633	91.06	84.09	95.93	76.51	64.20	86.98
Madhya Pradesh	Shajapur	634	81.49	75.21	87.32	80.15	72.43	87.04
Maharashtra	Palghar	635	93.06	86.78	97.19	NA	NA	NA
Maharashtra	Thane	636	95.64	87.60	99.19	90.25	80.66	96.29
Meghalaya	East Garo Hills	637	87.90	82.63	92.43	88.13	75.97	95.96
Meghalaya	East Jantia Hills	638	97.01	94.42	98.72	94.91	89.17	98.19
Meghalaya	North Garo Hills	639	69.53	62.06	76.69	89.92	85.09	93.78
Meghalaya	South West Garo Hills	640	88.95	83.33	93.33	85.70	74.55	93.70
Meghalaya	South West Khasi Hills	641	96.21	92.98	98.34	95.07	87.44	99.03
Meghalaya	West Garo Hills	642	85.30	79.96	90.40	90.10	85.36	94.24
Meghalaya	West Jaintia Hills	643	94.43	90.50	97.31	93.45	88.49	97.03
Meghalaya	West Khasi Hills	644	96.85	94.19	98.74	95.95	92.09	98.35
Punjab	Fazilka	645	92.44	88.08	95.92	90.08	82.31	95.51
Punjab	Firozpur	646	92.13	86.99	96.10	94.05	88.56	97.60
Punjab	Gurdaspur	647	95.14	91.12	97.96	92.75	87.30	96.68
Punjab	Pathankot	648	91.86	85.32	96.33	79.79	63.44	91.74
Telangana	Adilabad	649	84.18	77.52	89.85	89.48	76.15	96.83
Telangana	Bhadradri Kothagudem	650	85.57	78.42	91.52	67.83	55.23	79.54
Telangana	Jagitial	652	87.35	81.43	92.24	87.39	76.45	94.95
Telangana	Jangoan	653	86.48	79.48	92.00	NA	NA	NA
Telangana	Jayashankar Bhupalapally	654	95.53	91.95	98.00	85.36	69.34	95.16
Telangana	Jogulamba Gadwal	655	81.58	74.49	87.71	75.71	51.12	92.86
Telangana	Kamareddy	656	75.53	67.44	82.71	83.02	73.65	90.44
Telangana	Karimnagar	657	84.50	76.79	90.77	86.47	75.48	94.30
Telangana	Khammam	658	90.24	84.58	94.70	67.01	50.49	81.48

Telangana	Komaram Bheem Asifabad	659	90.00	84.55	94.31	64.97	47.03	81.41
Telangana	Mahabubabad	660	85.86	79.16	91.42	81.52	71.12	90.20
Telangana	Mahabubnagar	661	91.30	85.32	95.64	84.39	70.08	93.93
Telangana	Mancherial	662	91.50	85.57	95.91	87.48	64.66	97.96
Telangana	Medak	663	80.50	73.58	86.41	NA	NA	NA
Telangana	Medchal-Malkajgiri	664	94.90	82.88	99.46	88.92	69.45	98.25
Telangana	Nagarkurnool	665	89.16	83.52	93.58	90.83	81.40	96.68
Telangana	Nalgonda	666	91.94	86.28	96.05	77.81	66.69	87.02
Telangana	Nirmal	667	86.74	79.86	92.42	67.41	55.38	78.47
Telangana	Nizamabad	668	78.16	69.03	86.28	79.35	65.49	90.34
Telangana	Peddapalli	669	90.83	84.69	95.39	87.63	76.52	95.13
Telangana	Rajanna Sircilla	670	88.62	82.88	93.53	82.30	56.37	96.20
Telangana	Ranga Reddy	671	87.04	77.54	94.08	82.14	72.76	90.04
Telangana	Sangareddy	672	77.61	67.10	86.32	79.38	68.85	88.03
Telangana	Siddipet	673	77.58	69.14	84.76	80.10	65.45	90.79
Telangana	Suryapet	674	83.01	76.11	88.64	76.90	65.17	86.86
Telangana	Vikarabad	675	87.18	80.68	92.52	NA	NA	NA
Telangana	Wanaparthy	676	87.46	81.08	92.53	90.40	77.49	97.31
Telangana	Warangal Rural	677	80.93	73.74	87.32	89.82	81.44	95.69
Telangana	Warangal Urban	678	85.93	74.67	94.22	76.55	53.06	92.99
Telangana	Yadadri Bhuvanagiri	679	91.85	86.81	95.79	66.69	48.89	83.29
Tripura	Gomati	680	86.28	79.42	91.76	91.35	84.43	96.11
Tripura	Khowai	681	86.59	80.60	91.64	90.85	83.15	96.11
Tripura	North Tripura	682	93.77	89.07	97.11	89.28	82.06	94.52
Tripura	South Tripura	683	93.10	88.23	96.57	90.87	85.36	94.98
Tripura	Unakoti	684	94.32	90.26	97.09	88.60	82.39	93.57
Tripura	West Tripura	685	95.15	89.59	98.37	94.05	88.88	97.55
Uttar Pradesh	Amethi	686	91.48	86.97	95.02	78.59	70.47	85.70
Uttar Pradesh	Budaun	687	82.38	75.80	88.32	85.39	79.44	90.18
Uttar Pradesh	Ghaziabad	688	94.25	83.83	98.88	77.06	68.30	84.50
Uttar Pradesh	Hapur	689	91.81	87.26	95.37	76.66	69.09	83.64
Uttar Pradesh	Moradabad	690	90.73	84.51	95.41	88.93	83.36	93.33
Uttar Pradesh	Muzaffarnagar	691	90.26	84.81	94.67	77.75	68.46	85.62
Uttar Pradesh	Rae Bareli	692	79.43	72.83	85.42	76.68	69.83	82.75
Uttar Pradesh	Sambhal	693	80.28	73.72	86.12	85.84	78.04	92.13
Uttar Pradesh	Shamli	694	91.10	86.13	95.05	73.35	56.21	86.81

Uttar Pradesh	Sultanpur	695	88.25	82.68	92.82	79.46	65.45	89.91
West Bengal	Paschim Barddhaman	696	94.80	85.98	98.83	94.08	85.18	98.53
West Bengal	Purba Barddhaman	697	86.70	81.01	91.56	87.91	78.95	94.22
Tamil Nadu	Thiruvallur	698	95.00	89.55	98.21	80.69	73.36	86.89
Gujarat	Surat	699	96.58	91.60	99.20	77.07	66.38	86.50
Gujarat	Amreli	700	95.84	91.86	98.38	78.27	71.54	83.99
Chhattisgarh	Surajpur	701	93.16	88.73	96.48	89.41	83.17	94.14
Madhya Pradesh	Sidhi	702	81.25	74.66	86.85	66.05	58.79	73.08
Uttar Pradesh	Kheri	703	86.51	80.85	91.30	87.97	82.39	92.63
Tripura	Sepahijala	704	87.56	81.98	92.16	90.29	85.13	94.35
Karnataka	Udupi	705	82.57	75.23	88.96	86.54	80.07	91.87
Kerala	Kasaragod	706	85.82	79.48	91.12	87.23	80.70	92.41
Lakshadweep	Lakshadweep	707	94.04	86.21	98.41	86.94	73.61	95.38
Kerala	Kannur	708	95.84	90.55	98.69	95.11	90.23	98.14
Kerala	Alappuzha	709	97.20	93.36	99.20	94.40	89.65	97.50
Mizoram	Saiha	710	96.72	92.59	98.98	98.19	96.36	99.30
Andaman & Nicobar Islands	South Andaman	711	92.59	85.15	97.18	88.09	80.42	93.81
Tamil Nadu	Ramanathapuram	712	96.21	92.49	98.51	82.94	75.04	89.75
Andaman & Nicobar Islands	Nicobars	713	96.39	92.71	98.63	84.59	78.37	90.18
Puducherry	Karaikal	714	92.31	86.30	96.73	71.40	61.88	80.12
West Bengal	Haora	715	87.94	79.84	94.04	88.93	82.27	94.24
Odisha	Jagatsinghapur	716	84.51	77.80	90.20	86.00	80.10	90.97
Andaman & Nicobar Islands	North & Middle Andaman	717	96.88	93.49	98.92	96.08	92.66	98.31
Ladakh	Kargil	718	81.14	73.79	87.46	87.48	82.99	91.33
Bihar	Aurangabad	720	82.64	76.57	88.02	69.35	62.01	76.32
Maharashtra	Aurangabad	721	86.76	80.26	92.32	88.89	81.65	94.63
Dadra & Nagar Haveli And Daman & Diu	Diu	722	97.62	94.04	99.41	93.74	88.82	97.04

Note. Districts missing data for both years were excluded from this table.
